# TOR-autophagy branch signaling via Imp1 dictates plant-microbe biotrophic interface longevity

**DOI:** 10.1371/journal.pgen.1007814

**Published:** 2018-11-21

**Authors:** Guangchao Sun, Christian Elowsky, Gang Li, Richard A. Wilson

**Affiliations:** 1 Department of Plant Pathology, University of Nebraska-Lincoln, Lincoln, Nebraska, United States of America; 2 Department of Agronomy and Horticulture, University of Nebraska-Lincoln, Lincoln, Nebraska, United States of America; Max-Planck-Institut fur Evolutionsbiologie, GERMANY

## Abstract

Like other intracellular eukaryotic phytopathogens, the devastating rice blast fungus *Magnaporthe* (*Pyricularia*) *oryzae* first infects living host cells by elaborating invasive hyphae (IH) surrounded by a plant-derived membrane. This forms an extended biotrophic interface enclosing an apoplastic compartment into which fungal effectors can be deployed to evade host detection. *M*. *oryzae* also forms a focal, plant membrane-rich structure, the biotrophic interfacial complex (BIC), that accumulates cytoplasmic effectors for translocation into host cells. Molecular decision-making processes integrating fungal growth and metabolism in host cells with interface function and dynamics are unknown. Here, we report unanticipated roles for the *M*. *oryzae* Target-of-Rapamycin (TOR) nutrient-signaling pathway in mediating plant-fungal biotrophic interface membrane integrity. Through a forward genetics screen for *M*. *oryzae* mutant strains resistant to the specific TOR kinase inhibitor rapamycin, we discovered *IMP1* encoding a novel vacuolar protein required for membrane trafficking, V-ATPase assembly, organelle acidification and autophagy induction. During infection, Δ*imp1* deletants developed intracellular IH in the first infected rice cell following cuticle penetration. However, fluorescently labeled effector probes revealed that interface membrane integrity became compromised as biotrophy progressed, abolishing the BIC and releasing apoplastic effectors into host cytoplasm. Growth between rice cells was restricted. TOR-independent autophagy activation in Δ*imp1* deletants (following infection) remediated interface function and cell-to-cell growth. Autophagy inhibition in wild type (following infection) recapitulated Δ*imp1*. In addition to vacuoles, Imp1^GFP^ localized to IH membranes in an autophagy-dependent manner. Collectively, our results suggest TOR-Imp1-autophagy branch signaling mediates membrane homeostasis to prevent catastrophic erosion of the biotrophic interface, thus facilitating fungal growth in living rice cells. The significance of this work lays in elaborating a novel molecular mechanism of infection stressing the dominance of fungal metabolism and metabolic control in sustaining long-term plant-microbe interactions. This work also has implications for understanding the enigmatic biotrophy to necrotrophy transition.

## Author summary

Plant-associated fungi can form intimate connections with living host cells. Clarifying the molecular drivers of these interactions, and which partner is dominant, might be important in understanding how beneficial plant-fungal relationships can be enhanced to improve crop yields while pathogenic interactions that threaten crop health are disrupted. In common with other symbionts and phytopathogens, the devastating rice blast fungus *Magnaporthe oryzae* elaborates invasive hyphae in living host cells surrounded by plant-derived membranes. Nothing is known at the molecular signaling level about how such plant-microbe biotrophic interfacial zones are maintained as the fungus grows in and between host cells. Here, we report that fungal membrane trafficking processes controlled by nutrient signaling pathways are critical for maintaining biotrophic interface integrity during *M*. *oryzae* growth in rice cells. Impairing these processes resulted in erosion of the plant-microbe interface and failure of the fungus to thrive. To our knowledge, this work presents the first evidence indicating that the fungal partner is dominant in propagating the plant-microbe boundary. This suggests that the biotrophic interface is a fungal construct and provides clues on how such interfaces might be modulated to benefit the host plant.

## Introduction

An intriguing feature of both beneficial and pathogenic plant-fungal interactions is the formation of biotrophic interfaces that facilitate nutrient acquisition and microbial growth in living host cells. Such interfaces comprise of a hyphal plasma membrane and cell wall, an interfacial matrix, and a plant-derived membrane [1, 2]. Although intrinsic to many important crop diseases, nothing is known at the molecular level about how such plant-microbe interfacial zones are regulated and maintained as the fungus elaborates hyphae in and between host cells. The blast fungus *Magnaporthe oryzae*, cause of devastating rice and wheat losses [3, 4], initially colonizes living rice cytoplasm as a symptomless biotroph [3, 5, 6]. Once penetration pegs emerging from specialized appressorial infection cells on the leaf surface have breached the rice cuticle into underlying epidermal cells, they expand into thin primary hyphae that elaborate branched, bulbous intracellular invasive hyphae (IH) enclosed in plant-derived extra-invasive hyphal membranes (EIHM). A neckband forms an apoplastic interfacial compartment where apoplastic effectors like Bas4 [7] and Slp1 [8] are deployed by the conventional fungal ER-Golgi secretion pathway [2, 7]. *M*. *oryzae* also forms a focal plant-membrane rich structure outside IH called the biotrophic interfacial complex (BIC) which forms in each newly infected rice cell until the lifestyle switch to necrotrophy. The BIC accumulates cytoplasmic effectors like Pwl2, destined for translocation into host cells, via an unconventional secretory pathway involving exocyst and SNARE proteins [2, 7]. Biotrophic interfaces thus facilitate effector deployment for the avoidance or suppression of plant innate immunity, and the intimate association between fungal hyphae and host plant cell-derived membranes is critical to the success of the infection process.

In addition to effector secretion, the suppression of plant innate immunity requires robust fungal antioxidation systems to neutralize host reactive oxygen species (ROS) that otherwise trigger growth-restricting plant defense responses [9, 10]. Recently, correct BIC formation in *M*. *oryzae* was found to be dependent on neutralizing the plant oxidative burst in a carbon- and nitrogen signaling-dependent manner via the fungal nitrooxidative stress response [11]. These results indicated both that antioxidation is a cardinal event during infection, and that plant defense suppression and fungal development are linked via the regulation of fungal metabolism. This previous study also illustrated how plant and fungal physiology are intimately connected and carefully balanced during *in planta* growth such that a mutation in the fungus (loss of the nitronate monooxygenase-encoding gene *NMO2* required for the *M*. *oryzae* nitrooxidative stress response) resulted in a response from the plant (ROS accumulation and the elicitation of host innate immunity) which affected the development of the fungus (multi-BIC formation and impaired growth).

We seek detailed insights on the metabolic regulation of fungal physiology during host infection with a long-term goal of understanding how fungal metabolism is connected to plant defense suppression. Recently, the *M*. *oryzae* Target-of-Rapamycin (TOR) signaling pathway has emerged as a key component of the rice infection process [12–14]. TOR kinase function is conserved in eukaryotes and integrates nutritional cues with cell growth and development by controlling central metabolism, ribosome biosynthesis and protein translation in response to amino acids, glucose and energy [15]. In yeast, active TOR signaling directly represses autophagy by phosphorylation of the autophagy-related (Atg) protein Atg13. Under nutrient-limiting conditions, TOR signaling is inactivated, anabolic processes are repressed and autophagy is induced by the dephosphorylation of Atg13 resulting in the assembly of the Atg1 protein kinase complex and the induction of macroautophagy (autophagy) [15]. Atg13 is a direct target of the yeast TORC1 complex, although the Tap42-PPase branch of the TOR signaling pathway is also involved in autophagy induction [15]. In *M*. *oryzae* (which unlike yeast only carries one *TOR* gene and it is not known if TOR signaling in *M*. *oryzae* involves complexes equivalent to yeast TORC1 and TORC2), the inactivation of TOR signaling is required during spore germination on the nutrient-free leaf surface in order to elaborate a functional appressorium. TOR inactivation is mediated by the novel TOR regulator Abl1 and maintained by low levels of intracellular glucose [14] and glutamine [13]. Inactive TOR engages two metabolic checkpoints at G2 and G1/G0 during spore germination in order to limit mitosis and induce autophagy and appressorium morphogenesis [14]. Conversely, once a mature appressorium has successfully penetrated into host cells, ATP production–stimulated following a metabolic switch to glucose metabolism in response to glucose-6-phosphate/ NADPH sensing by Tps1 [16–18]—activates TOR signaling, resulting in mitosis that facilitates early biotrophic growth [12]. The activity status of *M*. *oryzae* TOR signaling is critical to rice infection because activating TOR during spore germination results in multiple rounds of mitosis [14] and the loss of autophagy and appressorial development [13, 14], while preventing TOR activation following host penetration attenuates mitosis in IH and curtails biotrophic growth [12].

By considering the importance of TOR signaling to both pre- and post-penetration infection stages, the motivation for this study was to identify and characterize additional TOR pathway components in *M*. *oryzae*. Using forward and reverse genetics, pharmacological treatments and confocal microscopy, we discovered and characterized *IMP1* encoding a vacuolar-localized protein that is required for vacuole function and membrane trafficking and also outlines IH during growth *in planta*. Imp1 acts downstream of TOR kinase and is required for autophagy induction in response to inactivated TOR signaling. Loss of the TOR-Imp1-autophagy signaling axis attenuated fungal growth in rice cells, abolished BIC formation and, as biotrophy progressed, resulted in the inappropriate release of apoplastic effectors into host cytoplasm, indicating that the ability to maintain biotrophic interface membrane integrity during sustained growth was compromised over time. We show this membrane defect was not due to the loss of vacuole function *per se*, nor due to early entry into necrotrophy, and propose it more likely results from impaired membrane trafficking and recycling through the fungal vesicular network. Imp1 thus unexpectedly connects metabolic signaling by TOR to autophagy-dependent membrane homeostasis, biotrophic interface maintenance and fungal growth in plant cells. Given that *M*. *oryzae*—rice biotrophic interfaces are constructed from both fungal and plant membranes, the results presented here are surprising and significant in highlighting the importance of the metabolic status of the fungal cell to the longevity of the plant-microbe interaction.

## Results

### Random insertional mutagenesis using ATMT uncovers *IMP1* as a novel mediator of TOR-autophagy branch signaling

#### Loss of *IMP1* function confers rapamycin resistance

The rationale for this study was that new information on drivers of fungal pathogenicity—likely applicable to a range of important pathosystems—might come from a better understanding of TOR signaling in *M*. *oryzae*. Employing a forward genetics approach, we uncovered *IMP1* as a previously unknown mediator of TOR-autophagy branch signaling essential for rice infection. *IMP1* was discovered in a genome-wide, unbiased manner by screening *M*. *oryzae* mutant strains—generated by random insertional mutagenesis employing *Agrobacterium tumefaciens*-meditated transformation (ATMT)–for resistance or insensitivity to the specific TOR kinase inhibitor rapamycin. Rapamycin inhibits TOR signaling and, like for yeast [19], arrests *M*. *oryzae* growth [12] on plate media (**S1A Fig**). Following ATMT, only six rapamycin resistant mutant strains (AT1 to AT6) were recovered to purity (**S1A Fig**). In order to determine which mutant strain(s) to focus on, our initial assessments found that only the ATMT transformant designated AT2 produced spores (**S1B Fig**). This suggested that mutants carrying the genetic lesion resulting in AT2 would be amenable to downstream analyses of infection-related development and pathogenicity on rice hosts. AT1 and AT3-AT6 were thus not studied further.

**Fig 1:**
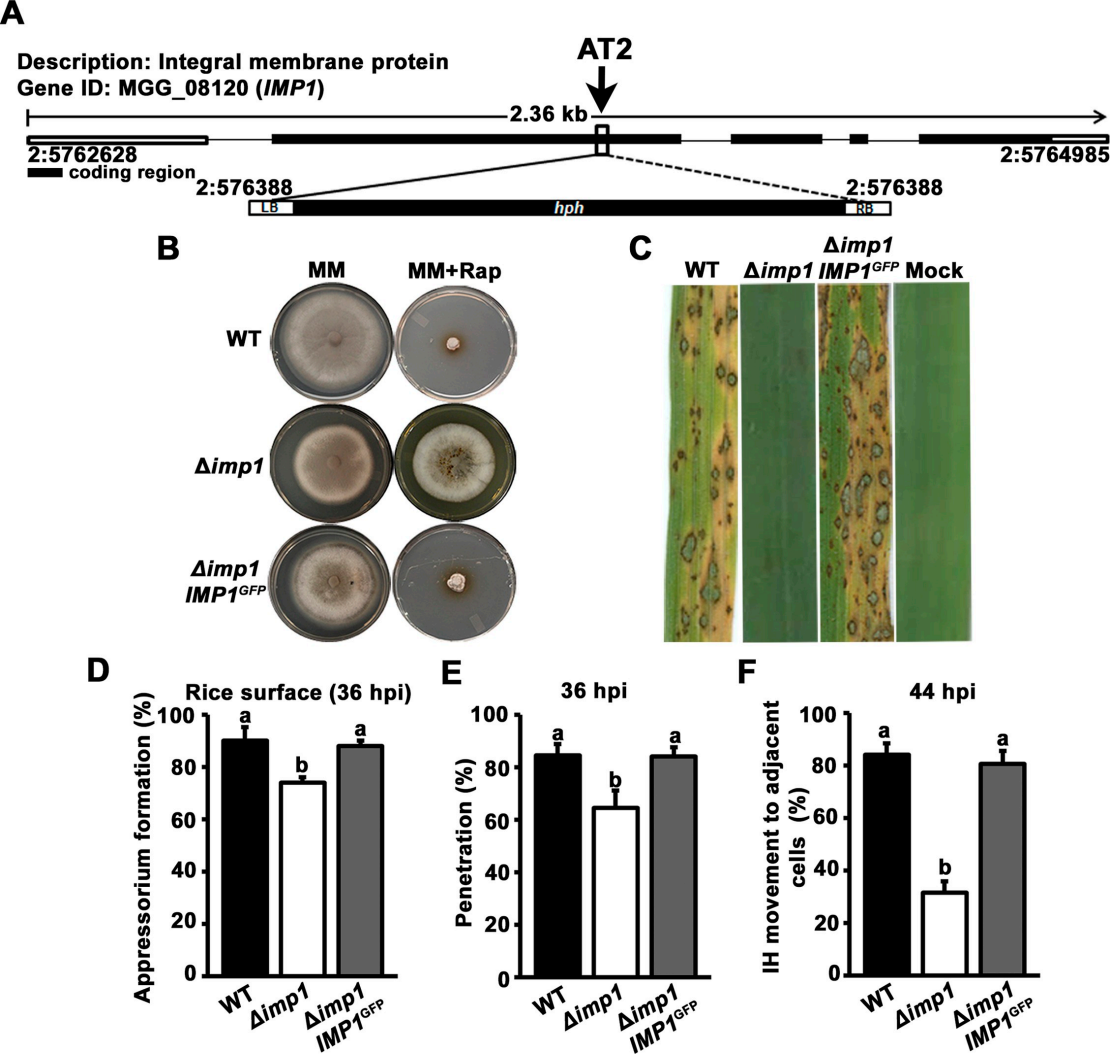
*IMP1* confers rapamycin sensitivity and is required for rice blast disease. (A) *Agrobacterium tumefaciens*-mediated transformation generated a rapamycin resistant strain, AT2, resulting from a T-DNA insertion event at MGG_08120 encoding an integral membrane protein (Imp1). LB and RB are the known left flank and right flank T-DNA sequences, respectively. (B) Targeted deletion of *IMP1* in the wild type (WT) strain Guy11 recapitulated AT2 by conferring rapamycin resistance to Δ*imp1* strains. Δ*imp1 IMP1*^*GFP*^ is the Δ*imp1* mutant strain complemented with the *IMP1* gene fused to the GFP-encoding cassette. Strains were grown on minimal media (MM) containing 1% (w/v) glucose and 10 mM NH_4_^+^ as the sole carbon and nitrogen source, respectively, and on MM supplemented with 10 μM rapamycin (Rap), for 12 days. (C) The Δ*imp1* mutant strain was non-pathogenic on seedlings of the susceptible rice cultivar CO-39 compared to WT and the Δ*imp1 IMP1*^*GFP*^ complementation strain. (D) Loss of *IMP1* marginally reduced appressorium formation rates on rice leaf surfaces compared to WT by 36 hpi. Values are the average percentage of appressoria formed by 50 germinating spores of each strain per rice cuticle, repeated in triplicate. (E) The penetration of Δ*imp1* appressoria through the rice cuticle and into underlying epidermal cells was reduced but not abolished compared to WT and the Δ*imp1 IMP1*^*GFP*^ complementation strain. Bars are the average percentage of penetration pegs developed at 36 hpi by 50 appressoria of each strain per rice cuticle, repeated in triplicate. (F) At 44 hpi, the Δ*imp1* mutant strain was impaired in cell-to-cell movement compared to WT and the Δ*imp1 IMP1*^*GFP*^ complementation strain. Bars are the average of 50 primary infected rice cells from which invasive hyphae (IH) were shown emerging into adjacent rice cells. Experiments were repeated in triplicate. (D-F) Error bars are s.d. Bars with different letters are significantly different (α ≤ 0.05, Least significant difference (LSD)).

To determine the nature of the genetic lesion in AT2 caused by ATMT and resulting in rapamycin resistance, we used TAIL PCR and the known *hph* and T-DNA sequences [9] to liberate T-DNA flanks and adjacent *M*. *oryzae* genome sequences. For reasons we were unable to ascertain, only PCR products from the left T-DNA flank were generated and subcloned. Nonetheless, the resulting sequences, when BLASTed at NCBI and Ensembl Fungi, revealed that T-DNA had integrated into the *M*. *oryzae* genome downstream of nucleotide 5763887, Supercontig 2, in the allele MGG_08120. This allele is annotated as encoding an uncharacterized 433 amino acid, 59 kDa integral membrane protein with five transmembrane domains which we name Imp1 (**Fig 1A**).

NCBI BLAST analysis showed that the Imp1 protein putatively carries a heme-binding cellobiose dehydrogenase (CDH) -like cytochrome domain (CDH-cyt), part of the DOMON domain superfamily, at the N-terminus. The C-terminus carries a cytochrome b561/ferric reductase transmembrane domain. The cellobiose dehydrogenase enzyme from *Aspergillus nidulans*, An7230, and the five cellobiose dehydrogenases annotated in *M*. *oryzae*, carry a GMC oxidoreductase domain instead of the cytochrome b561/ferric reductase transmembrane domain, suggesting Imp1 is not a cellobiose dehydrogenase enzyme. Indeed, the Imp1 amino acid sequence and *IMP1* nucleotide sequence do not align with the five cellobiose dehydrogenase gene sequences annotated in the *M*. *oryzae* genome. Moreover, whereas cellobiose dehydrogenases are secreted, Imp1 is predicted (and shown below) to be organelle localized. BLAST analysis of the Imp1 protein sequence at the *Saccharomyces* genome database shows that the top hit, albeit with low identity (31% over 157 amino acids), is to the 889 amino acid subunit a of the vacuolar- H^+^ ATPase (V-ATPase) V_0_ domain encoded at YMR054W (see below).

To confirm AT2 resulted from the disruption of MGG_08120, we used split-marker homologous recombination [17] to replace the entire *IMP1* coding region in our wild type (WT) Guy11 strain with the *ILV1* selectable marker conferring resistance to sulphonyl urea. **Fig 1B** shows that a clean deletion of *IMP1* in the WT background recapitulated the AT2 phenotype by conferring rapamycin resistance to the Δ*imp1* mutant strain. Introducing a copy of *IMP1*—under its native promoter and fused to the gene encoding green fluorescent protein (GFP)—into the Δ*imp1* deletant restored rapamycin sensitivity in the resulting Δ*imp1 IMP1*^*GFP*^ complementation strain (**Fig 1B**), thus confirming that rapamycin resistance was solely due to the loss of *IMP1* function.

#### *IMP1* is required for biotrophic growth in rice cells

Sporulation rates of the Δ*imp1* deletant were only marginally reduced on complete media (CM) compared to WT and the Δ*imp1 IMP1*^*GFP*^ complementation strain (**S2A Fig**), and Δ*imp1* generated quantities of spores sufficient for downstream applications. Equal numbers of spores of WT, Δ*imp1* and the Δ*imp1 IMP1*^*GFP*^ complementation strain were applied to the leaves of rice seedlings of the susceptible cultivar CO-39. *IMP1* was found to be essential for rice infection (**Fig 1C**) and is therefore a new determinant of fungal pathogenicity warranting further characterization.

**S2B Fig** shows that, by 24 hpi on artificial hydrophobic surfaces, Imp1^GFP^ localized to clustered compartments in mature appressoria. In Δ*imp1* mutant strains, appressorium formation on artificial hydrophobic surfaces was stochastic, with 69% of germinating Δ*imp1* spores producing no appressoria by 24 hpi (**S2C Fig**). By contrast, on rice leaf surfaces, about 70% of germinating Δ*imp1* spores formed appressoria when assessed at 36 hpi (**Fig 1D**). This is reduced but comparable to the 90% appressorium formation rates observed for WT and the Δ*imp1 IMP1*^*GFP*^ complementation strain. **Fig 1E** shows that the rate of Δ*imp1* appressorial penetration into host cells was also marginally reduced but not abolished compared to WT and the Δ*imp1 IMP1*^*GFP*^ complementation strain. However, the biggest difference between Δ*imp1* and WT was observed in the rates at which IH from the first infected rice cell had spread to adjacent cells by 44 hpi: about 35% of Δ*imp1* IH in primary infected rice cells had developed IH in adjacent cells by 44 hpi, compared to about 85% for WT and the complementation strain (**Fig 1F and S2D Fig**). Taken together, while acknowledging that *IMP1* is required for robust, non-stochastic appressorial development on artificial hydrophobic surfaces, we conclude that the major role of *IMP1* during rice infection is in promoting biotrophic growth. We thus next focused on understanding the biotrophic growth aspect of the Δ*imp1* phenotype.

#### *IMP1* is required for TOR signaling through the autophagy pathway branch

**Fig 2:**
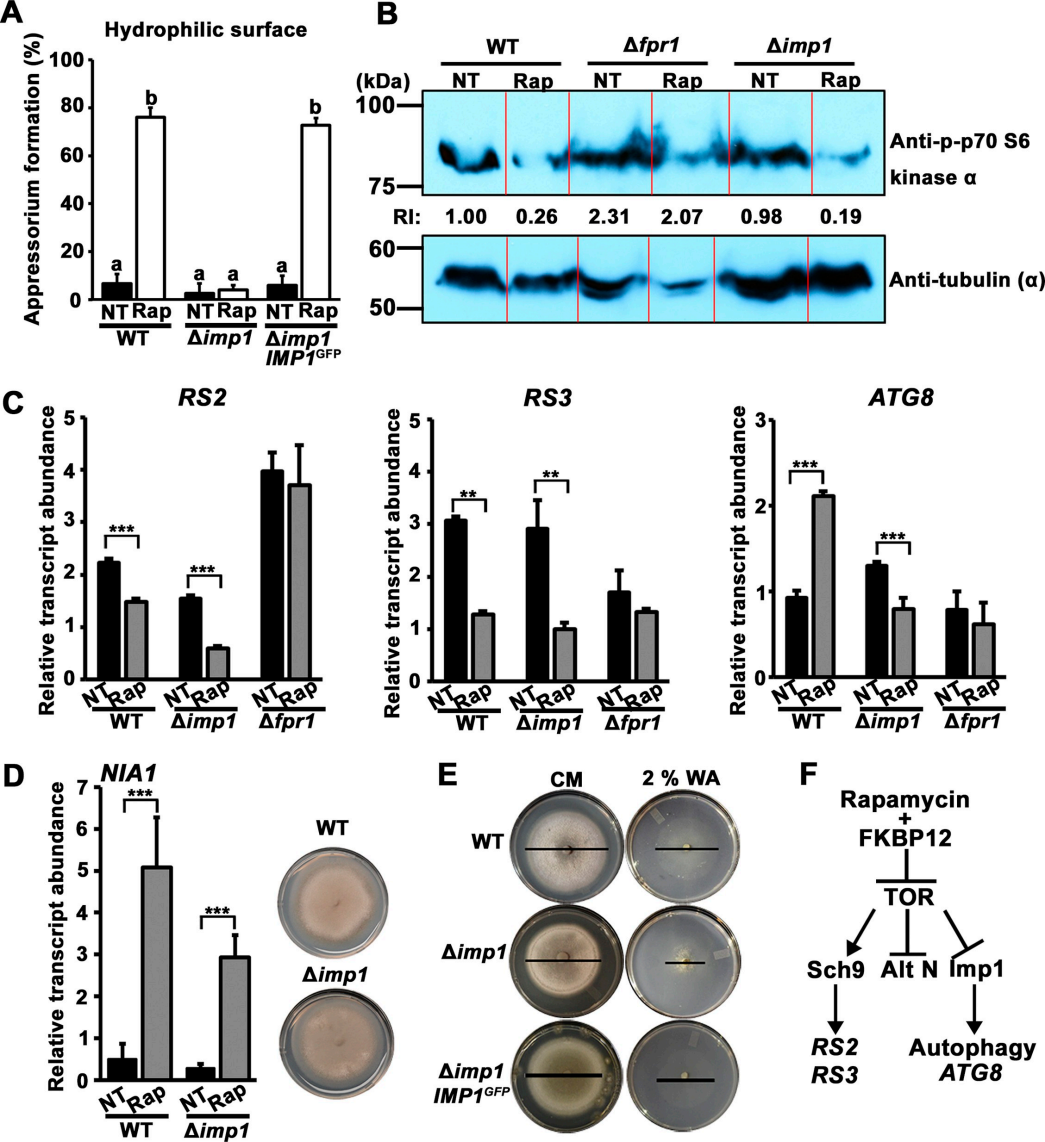
*IMP1* acts downstream of TOR kinase in the TOR-autophagy signaling branch. (A) Spore suspensions of WT, Δ*imp1* and Δ*imp1 IMP1*^*GFP*^ strains were either not treated (NT) or treated with rapamycin to a final concentration of 200 nM and applied to non-inductive hydrophilic glass slides. Bars are the mean percentage of the number of appressoria formed from 50 germinating spores, replicated on three different slides, by 24 hpi. Bars with different letters indicate significant difference (α ≤ 0.05, LSD). Error bars are s.d. (B) Western blot showing the phosphorylation status of the direct TOR kinase target Sch9 in the indicated strains following treatment with 1 μM rapamycin (Rap) for 8h. Strains were grown in complete media (CM). NT = no treatment. RI = relative intensity calculated by normalizing Sch9 phosphorylation levels determined using anti-p-p70 S6 kinase antibody against tubulin α levels determined by anti-tubulin α antibody. Red lines mark lane boundaries used for densitometry. When the ends of neighbouring bands fused during gel running and made lane demarcation difficult, the boundary was placed between adjacent band tails. (C) Quantitative real-time PCR (qPCR) analysis of TOR-regulated genes in the indicated strains following 16 h growth in CM. NT = no treatment; Rap = 1 μM rapamycin treatment. Data represent mean values ± s.d. from two biological replicates with three technical replicates each (*Student’s t test* **p ≤ 0.001, ***p ≤ 0.0001), normalized against the expression of *TUB2* encoding β-tubulin. (D) *IMP1* is not required for the utilization of alternative nitrogen sources. *Left*, qPCR analysis shows *IMP1* is not required for the rapamycin-induced derepression of the nitrate reductase structural gene (*NIA1*). NT = no treatment; Rap = 1 μM rapamycin treatment. Data represent mean values ± s.d. from two biological replicates each with three technical replicates (*Student’s t test* **p ≤ 0.001, ***p ≤ 0.0001), normalized against the expression of *TUB2* encoding β-tubulin. *Right*, *IMP1* is not required for utilizing nitrate as an alternative nitrogen source. Strains were grown for 12 days on MM with 1% (w/v) glucose (Glc) and 10 mM nitrate (NO_3_^-^) as the sole carbon and nitrogen source, respectively. (E) Growth of WT, Δ*imp1* and the Δ*imp1*::*IMP1*^*GFP*^ complementation strain on complete media (CM) and 2% water agar (WA) after 10 days. For clarity, black lines indicate colony diameters. (D,E) Representative images from three experiments are shown. (F) Model showing the proposed relationship between rapamycin, TOR signaling and Imp1. Alt N is alternative nitrogen source utilization.

We reasoned that in order to understand how *IMP1* functions in biotrophy, we must first resolve the relationship between *IMP1*, rapamycin and TOR signaling. Four scenarios could account for rapamycin resistance in Δ*imp1* strains: 1) *IMP1* plays no role in TOR signaling, but the loss of *IMP1* relieves growth arrest resulting from rapamycin toxicity via an unrelated suppressing mechanism; 2) *IMP1* is required for the inhibition of TOR kinase by rapamycin; 3) The loss of *IMP1* constitutively activates TOR kinase or downstream TOR signaling, indirectly resulting in rapamycin resistance; or 4) *IMP1* is required for propagating all or part of the inactive TOR signal. To distinguish which scenario was most likely correct, we first determined the response of the Δ*imp1* mutant strain to rapamycin in a growth-independent manner by studying the effects of rapamycin treatment on appressorium development. We have previously demonstrated that treating germinating spores with rapamycin induces appressoria formation on otherwise non-inductive hydrophilic surfaces [13]. **Fig 2A** shows that in contrast to spores of WT and the Δ*imp1 IMP1*^*GFP*^ complementation strain, germinating Δ*imp1* spores did not develop appressoria on hydrophilic surfaces in response to rapamycin treatment. By divorcing the effect(s) of rapamycin on hyphal growth from the developmental response of germinating spores to rapamycin, this result implies that rapamycin resistance in Δ*imp1* most likely arises from the inability of rapamycin to inactivate TOR signaling, rather than by the suppression of growth defects *per se*. Thus we can rule out scenario 1 above.

We next asked if the loss of *IMP1* prevented rapamycin inhibiting TOR kinase. To address this, we assayed for the activity of TOR kinase in WT and Δ*imp1*, in the presence and absence of rapamycin, using a commercial antibody that detects the phosphorylated form of the AGC family kinase ribosomal protein S6 kinase beta-1 (S6K1). S6K1, also known as p70-S6 kinase, is a functional orthologue of yeast Sch9 [20] and a direct target of activated TOR kinase [21]. S6K1/Sch9 phosphorylation is a marker of TOR activation [15, 22], and rapamycin inhibits S6K1 [23] and Sch9 [24] phosphorylation. Our hypothesis was that following rapamycin treatment, S6K1/Sch9 phosphorylation would decrease in WT but not in Δ*imp1* if *IMP1* was required for TOR kinase inactivation by rapamycin, or if the loss of *IMP1* constitutively activated TOR signaling. As the positive control for our immuno-analysis of phosphorylated Sch9 in *M*. *oryzae*, we included the *M*. *oryzae* mutant strain Δ*fpr1*. *FPR1* encodes the FK506/rapamycin-binding protein FKBP12 that is required for TOR inhibition by rapamycin. Consequently, TOR signaling is not inactivated by rapamycin in the Δ*fpr1* mutant strain [13, 14]. Our western analysis showed, as expected, that Sch9 phosphorylation levels in WT, when normalized against α-tubulin, responded to rapamycin treatment and were reduced four-fold compared to the untreated sample, while Sch9 phosphorylation levels in Δ*fpr1* samples were unaffected by rapamycin (**Fig 2B**), indicating that TOR kinase activity was inhibited by rapamycin in WT but not in Δ*fpr1*. In Δ*imp1*, Sch9 phosphorylation levels were diminished about five-fold following rapamycin treatment compared to the untreated control, indicating TOR kinase activity in Δ*imp1* was, like WT, inhibited by rapamycin. These results reject our hypothesis that *IMP1* is required for TOR inhibition by rapamycin, and also show that the loss of *IMP1* does not constitutively activate TOR, thus ruling out scenarios 2 and 3 above and suggesting *IMP1* acts downstream of TOR kinase.

We next asked where *IMP1* was involved in downstream TOR signaling. To address this question, we used quantitative real-time PCR (qPCR) to study the expression of previously determined TOR readout genes [12] following the growth of WT, Δ*imp1* and Δ*fpr1* in CM with or without 1 μM rapamycin. As shown previously [12], *RS2* and *RS3* genes encoding ribosomal proteins were downregulated in WT following rapamycin treatment, while the autophagy gene *ATG8* was upregulated (**Fig 2C**). As predicted, gene expression in Δ*fr1* was not affected by rapamycin treatment, but in Δ*imp1*, *RS2* and *RS3* gene expression responded to rapamycin treatment like WT, supporting our notion that *IMP1* was not required for TOR inhibition by rapamycin. However, *ATG8* gene expression was not induced in Δ*imp1* in response to rapamycin treatment (**Fig 2C**), suggesting that the autophagy branch of TOR signaling, but not all downstream TOR pathway branches, might require *IMP1* for induction in response to TOR inactivation.

Additional evidence that *IMP1* is not required for all cellular responses to TOR inactivation is shown in **Fig 2D**. Fungi preferentially use certain nitrogen sources such as ammonium (NH_4_^+^) over less-preferred alternative or secondary nitrogen sources such as nitrate (NO_3_^-^) [25]. In yeast, the genes for utilizing poor nitrogen sources are derepressed following TOR inactivation under nitrogen-limiting conditions, or after rapamycin treatment [15]. It is not known if TOR similarly controlled nitrogen metabolism in *M*. *oryzae*, but **Fig 2D** shows that this is likely because the expression of *NIA1* encoding nitrate reductase is induced almost 10-fold in complete media after rapamycin treatment in both WT and Δ*imp1* strains. In addition, Δ*imp1* also grows like WT on NO_3_^-^ media. Thus, the loss of *IMP1* does not prevent the derepression of genes for alternative nitrogen source utilization following the inactivation of TOR signaling, indicating *IMP1* does not act in this branch of the TOR signaling pathway.

Additional evidence that *IMP1* might act in the autophagy branch of TOR signaling is shown in **Fig 2E**. Here, Δ*imp1* growth was more restricted on starvation media than WT or the Δ*imp1 IMP1*^*GFP*^ complementation strain.

We propose that these preliminary results, taken together, fit the model in **Fig 2F**. This model provides a framework for the elucidation of *IMP1* function by illustrating how *IMP1* is not required for inactivating TOR signaling in response to rapamycin, or for preventing constitutive TOR activation, but is instead a positive-acting downstream TOR signaling component mediating autophagy in response to inactivated TOR signaling. Because the relationship between Sch9 and autophagy in *M*. *oryzae* is unknown, and because autophagy in yeast can be regulated by TORC1 independently of Sch9 [26, 27], Sch9 and Imp1 are depicted in **Fig 2F** in separate branches of the TOR signaling pathway. *RS2* and *RS3* gene expression is depicted under Sch9 control based on studies in human and yeast, but this is not known in *M*. *oryzae*. We also do not know which TOR signaling branch controls nitrogen gene expression in *M*. *oryzae*, and nitrogen regulation might not be separated from the Sch9 branch as depicted here.

### Imp1^GFP^ localizes to the vacuole

**Fig 3:**
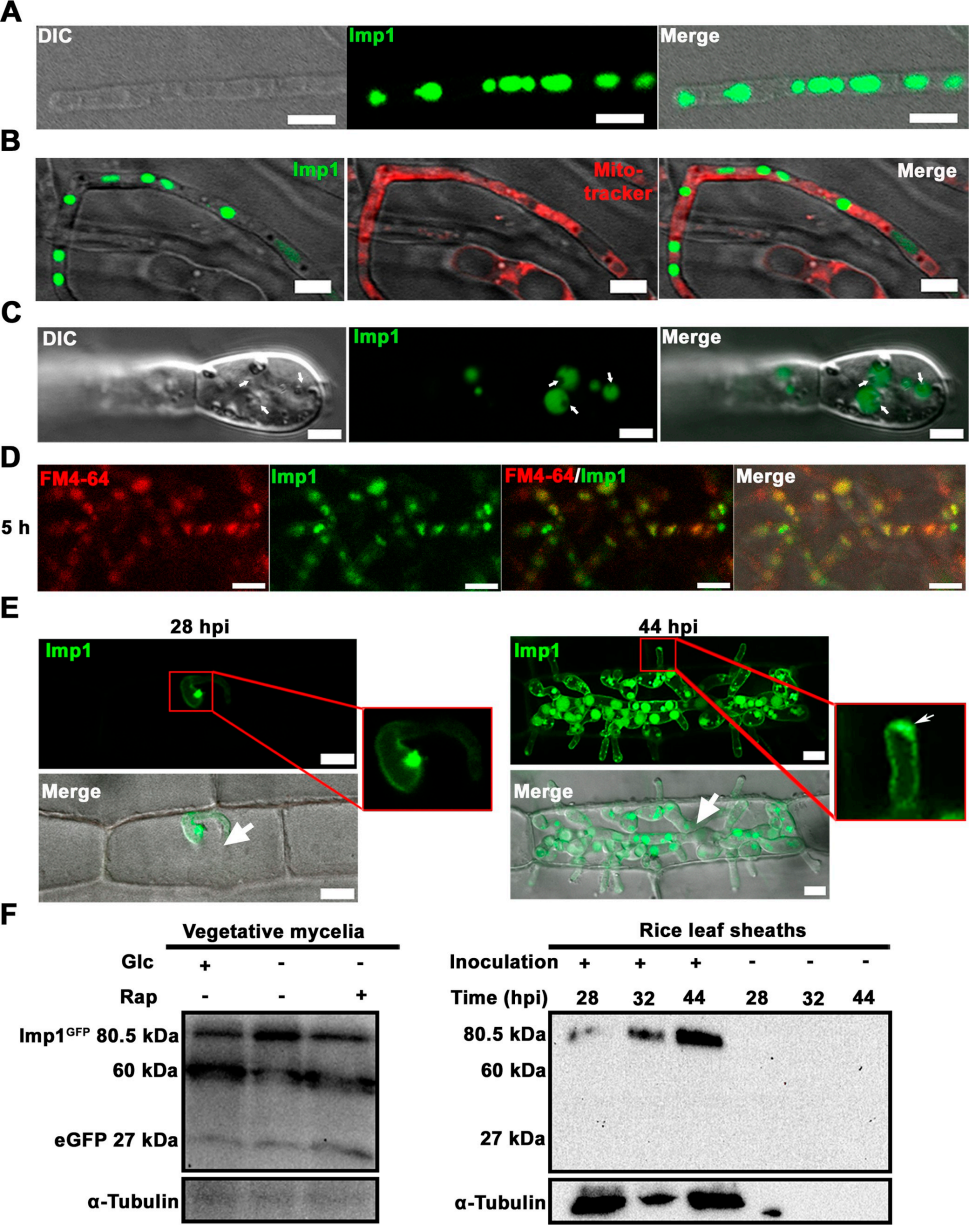
Imp1^GFP^ localizes to the vacuole. (A-D) Imp1^GFP^ localizes to vacuoles in vegetative mycelia after axenic growth for 16 h in liquid MM with glucose as the sole carbon source (GMM). (B) Mycelia were stained with mitotracker for 30 min before subjected to scanning laser confocal microscopy. (A-B) Scale bar = 10 μm. (C) Imp1^GFP^ localized to vacuoles associated with vesicles (arrows). Scale bar = 2.5 μm. (D) Imp1^GFP^ co-localized with the specific vacuolar stain FM4-64. Scale bar = 10 μm. (E) Leaf sheath infection assays showed that after penetration into epidermal cells, Imp1^GFP^ localized to a single vacuole and the IH plasma membrane at 28 hpi (*left*). By 44 hpi (*right*) Imp1^GFP^ localized to many internal compartments, the IH plasma membrane, and vacuoles at the tips of IH emerging into cells adjacent to primary infected cells (thin arrow in zoom box). Large arrows indicate appressoria on the leaf sheath surfaces. Scale bar = 10 μm. (F) Western blot analysis of Imp1^GFP^ using anti-GFP monoclonal antibodies to probe proteins extracted from vegetative mycelia (*left*), and from inoculated (+) and uninoculated (-) rice leaf sheaths (*right*). α-tubulin was used as the loading control. To explore whether growth conditions affect Imp1^GFP^ processing or modification, vegetative mycelia were grown in MM under normal 1% w/v glucose (Glc) sufficient conditions (+), or under glucose restrictive (0.025% w/v) conditions (-), with or without 1 μM rapamycin (Rap).

To shed light on how *IMP1* might function in autophagy, and thus progress towards an understanding of the role of *IMP1* in fungal pathogenicity, we examined where Imp1, a putative transmembrane protein, was localized during vegetative hyphal growth and *in planta* colonization. During axenic growth, the Imp1^GFP^ protein localized to internal compartments that were spaced throughout vegetative hyphae, and also clustered at the growing tip (**Fig 3A–3C**), where they were observed associating with small vesicles (indicated by arrows in **Fig 3C**). Furthermore, FM4-64, a lipophilic dye that selectively stains vacuolar membranes [28, 29], co-localized with compartments carrying Imp1^GFP^ (**Fig 3D**). Together, these results suggested that Imp1 localizes to vacuoles.

Live-cell imaging of rice leaf sheaths infected with the Δ*imp1 IMP1*^*GFP*^ complementation strain revealed that at 28 hours post inoculation (hpi), Imp1^GFP^ localized to a single compartment located between primary hyphae and early IH (**Fig 3E**, *left*). By 44 hpi, Imp1^GFP^ associated with many internal compartments in branching IH and, following IH movement to adjacent cells, was found localized at the emerging hyphal tip (**Fig 3E**, *right*). Interestingly, Imp1^GFP^ outlined IH (**Fig 3E**), suggesting a plasma membrane association that was not observed in vegetative hyphae (**Fig 3A–3C**).

To distinguish if Imp1 was likely a native vacuolar resident, or targeted to the vacuole for degradation, we performed western blot analyses, using monoclonal GFP antibodies, on total proteins isolated from vegetative hyphae (**Fig 3F** *left*) and infected and non-infected rice leaves (**Fig 3F** *right*). Under axenic growth conditions, an intact Imp1^GFP^ band was detected at 80.5 kDa under all growth conditions tested, although free GFP and some processing of Imp1^GFP^ also occurred. After leaf sheath infections, a single band was detected at the size corresponding to Imp1^GFP^, and no processing or free GFP was detected. Note that our commercial anti-tubulin α antibody obtained from yeast does not cross-react with plant tubulin. We conclude that Imp1 is likely a vacuolar protein, although some degradation, processing or turnover of Imp1^GFP^ in the vacuole is also occurring.

### *IMP1* is required for autophagy-associated organelle acidification in response to TOR signaling

**Fig 4:**
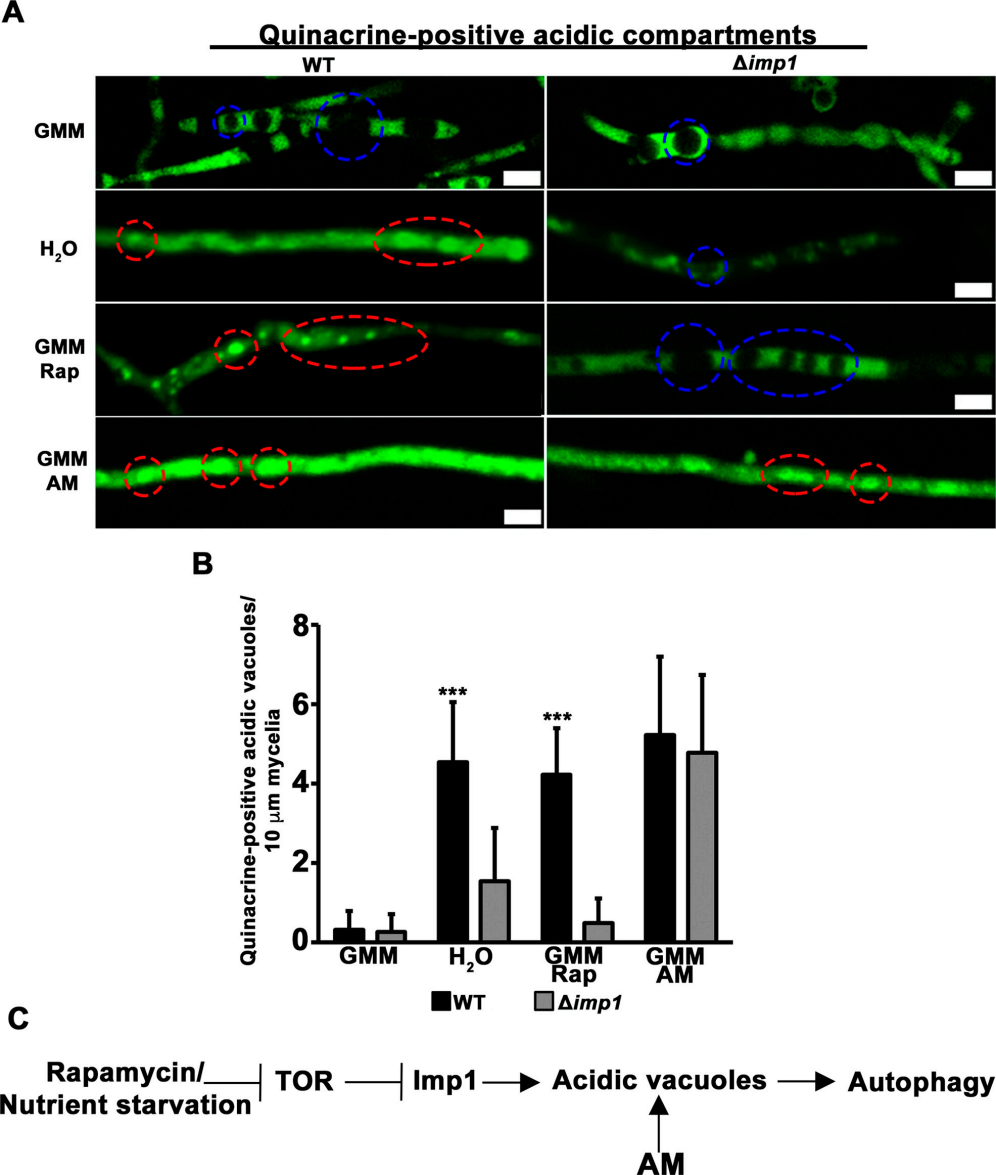
*IMP1* is required for organelle acidification in response to TOR signaling. (A) Staining of acidified compartments (including vacuoles) in vegetative mycelia with 1 μg/mL quinacrine after growth in the indicated treatments for 3 h. Rap = 1 μM rapamycin. AM = 1 μM amiodarone hydrochloride, a TOR-independent autophagy stimulator. GMM = glucose minimal media. Scale bar = 5 μm. Red dashed circles indicate examples of quinacrine-stained acidified vacuoles. Blue dashed circles indicate examples of unstained vacuoles. (B) Bars are the average number of quinacrine-positive vacuoles per 10 μm length of mycelia that were counted in 30 mycelia fragments longer than 100 μm, repeated in triplicate. *** indicates p value < 0.0001, *Students t-test*. Error bars are s.d. Mycelial length was measured by ImageJ. Contrast was adjusted to best distinguish vacuole boundaries for counting. (C) Imp1 acts downstream of TOR in the autophagy signaling branch and is required for vacuole acidification after autophagy induction by rapamycin treatment or nutrient starvation.

Autophagy induction increases the acidification of vacuoles in yeast [30, 31] and lysosomes in metazoan cells [32, 33]. Organelle acidification is mediated by the vacuolar (H^+^)-ATPase (V-ATPase) complex and is required for the hydrolytic enzyme activities that facilitate the terminal steps of autophagy [30]. Vacuole acidity is accompanied by vesicle docking and fusion [34–36], which together ensure functional autophagic flux [31, 32]. Mutants unable to form acidic vacuoles are subsequently defective in late-stage autophagy [30]. The vacuolar localization of Imp1^GFP^ suggested *IMP1* might play a role in vacuole function. To determine whether Imp1 was involved in vacuolar response(s) to autophagy induction, we first ascertained if differences in cell compartment acidity could be discerned between WT and Δ*imp1* under different nutrient and treatment regimes. Strains were grown in complete media (CM) for 48 h then transferred to fresh MM (with or without treatments) containing 1% (w/v) glucose (GMM), or into water, for 3 h before staining with 1 μg/ mL quinacrine for 15 min. Quinacrine is widely used as a reliable stain for acidified cellular compartments and targets the acidic vacuolar lumen [31, 37, 38]. **Fig 4A** shows that in WT, a switch into water substantially increased organelle acidification by 3 h when compared to growth in nutrient-rich GMM, indicating robust autophagy induction in response to starvation conditions. In contrast, although Δ*imp1* mycelia demonstrated a similar degree of compartment acidification as WT on GMM, the number of acidic vacuoles was not increased when transferred to water, indicating the loss of autophagy induction in Δ*imp1* strains under starvation conditions. Furthermore, vacuolar acidification was induced in WT but not Δ*imp1* mycelia when rapamycin was added to GMM, confirming Δ*imp1* is rapamycin insensitive. However, growth in GMM treated with amiodarone hydrochloride (AM) resulted in increased compartment acidification and acidic vacuoles in both WT and Δ*imp1* hyphae relative to growth in GMM alone. This is striking because AM is a TOR-independent autophagy inducer that acts via a mechanism involving Ca^2+^ [39–41] to increase autophagosome formation and degradation [41]. In fungi, AM treatment results in Ca^2+^ and H^+^ surges, and produces starvation responses similar to those observed with rapamycin treatment [42, 43]. **Fig 4B** quantifies the number of acidified vacuoles in WT and Δ*imp1* following the indicated treatments. When considered together, our results suggest that *IMP1* is required for organelle acidification and increasing acidic vacuoles during autophagy induction under starvation or rapamycin treatment conditions (**Fig 4C**). This model is consistent with our findings in **Fig 2** that indicated *IMP1* mediates autophagy downstream of TOR. Furthermore, because the degree of organelle acidification in GMM was similar between WT and Δ*imp1*, we conclude that *IMP1* encodes a previously unknown TOR-autophagy signaling branch component required for inductive autophagy in response to nutrient starvation or rapamycin treatment but not required for basal autophagy or AM-dependent autophagy induction.

### Imp1^GFP^ localization is unchanged during autophagy induction

We asked whether changes occurred to Imp1^GFP^ localization and/or vacuole morphology in response to autophagy induction. **S3 Fig** shows that Imp1^GFP^ localizes to vacuoles under the four conditions tested, but vacuoles became enlarged following transfer into H_2_O and AM treated GMM compared to GMM alone. Rapamycin treatment also resulted in Imp1^GFP^ localizing to larger compartments but with altered morphology. In this case, Imp1^GFP^ also outlined the plasma membrane. Therefore, vacuole morphology is responsive to autophagy induction, and rapamycin might also promote Imp1 localization to the plasma membrane, such as was only previously observed in IH.

### *IMP1* is required for vesicle trafficking

**Fig 5:**
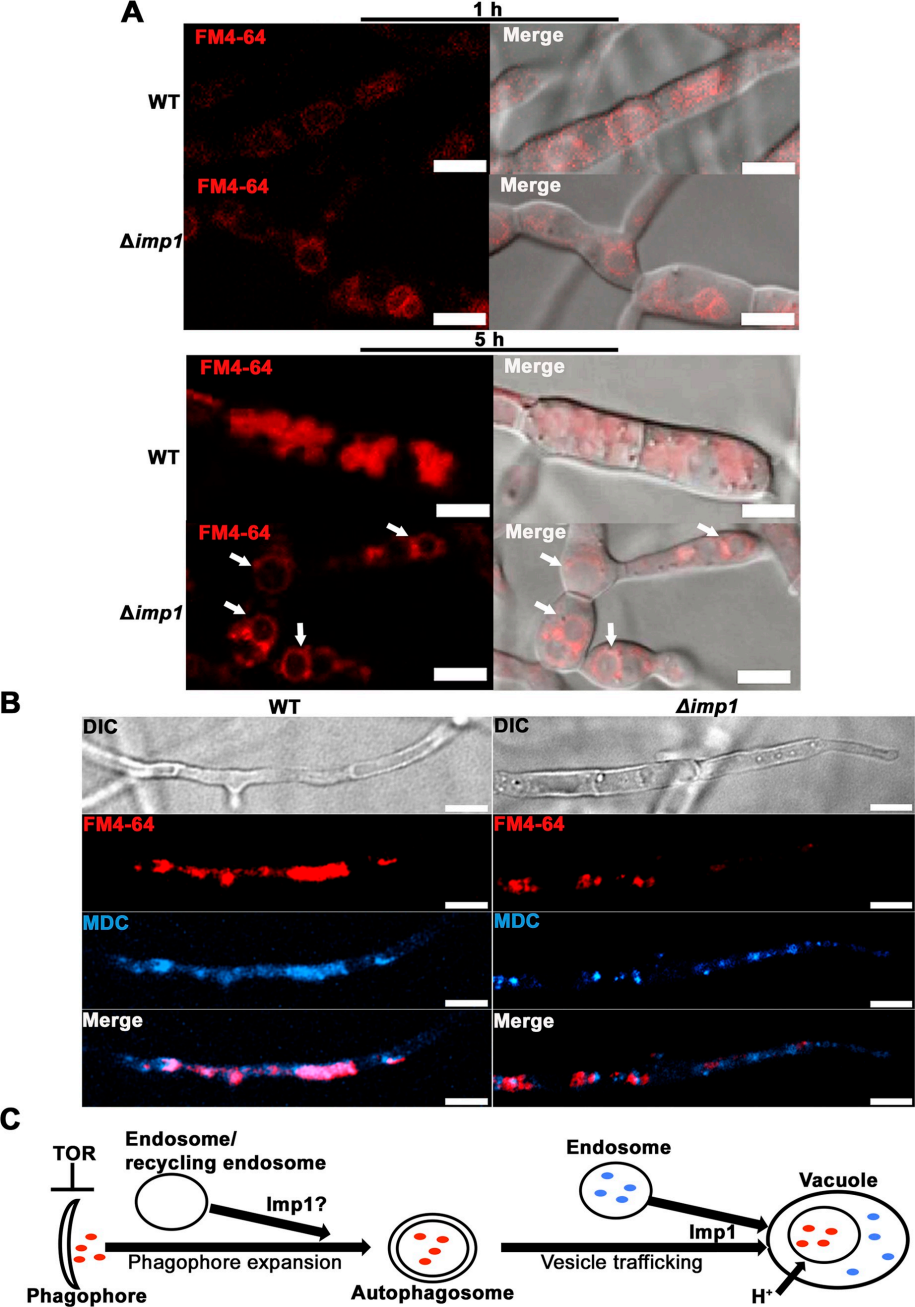
*IMP1* mediates endocytic and autophagic vesicle dynamics. (A) FM4-64 staining shows endocytic vesicles targeting vacuoles in Δ*imp1* and WT. After 5h, FM4-64 is not distributed throughout the full vesicular network in Δ*imp1* mycelia. Scale bar = 2 μm. (B) Monodansylcadaverine (MDC) staining revealed both reduced numbers of autophagic vacuoles in Δ*imp1* compared to WT, and reduced overlap with FM4-64 stained compartments. Mycelia from WT and *Δimp1* were incubated in GMM for 16 h then stained with 1 μg per ml FM4-64 and 40 μM MDC for 5 h in water. Scale bar = 10 μm. (C) *IMP1* is required for endosomal and autophagosomal membrane vesicle trafficking to acidified autophagic vacuoles, and might also be required for supplying endosomal membranes for phagophore expansion.

Vacuoles/lysosomes are the destination for vesicles from the endocytic and autophagic pathways, and vacuole/lysosome—vesicle fusion and vesicular trafficking is linked to organelle acidification [33, 44, 45]. To determine if vesicle trafficking was affected in Δ*imp1*, we first studied endocytosis using FM4-64, a fluorescent endocytic marker that stains vacuolar membranes in yeast and plants before becoming distributed throughout the full vesicular network, including secretory pathways [28, 29]. Strains were grown in GMM for 16 h prior to treatment. After 1 hour of treatment, FM4-64 had been internalized and stained vacuolar membranes in both WT and Δ*imp1* strains, as evidenced by the observed ring staining pattern, indicating endocytosis and endosome delivery to the vacuole was not impaired in the Δ*imp1* mutant (**Fig 5A**). After 5 h, FM4-64 staining retained the ring pattern in Δ*imp1* mycelia, while in WT, FM4-64 accumulated in vacuoles and was extensively distributed across the vesicular network (**Fig 5A**). This observation suggests that while endocytosis and the docking of endosomes to vacuoles is not impaired in Δ*imp1*, the fusion of endosomes to vacuoles is blocked or delayed.

We next sought to understand autophagic pathway dynamics in WT versus Δ*imp1*. Monodansylcadaverine (MDC) is a widely used stain for acidified autophagic vacuoles (which include autophagosomes, amphisomes and autolysosomes [46]). MDC has been used to stain autophagic bodies in *M*. *oryzae* [47]. MDC-labeled autophagic vacuoles are spatially separated from endosomal compartments, and acidic vacuoles/ lysosomes that have recently fused with autophagosomes can also be stained [48]. In mammalian cells, autophagosomes can fuse directly with endosomes to become amphisomes before fusion with vacuoles/lysosomes [46], but yeast do not form amphisomes, and autophagosomes and endosomes fuse directly to vacuoles in fungi [49]. Vesicular membrane trafficking from different subcellular compartments is regulated by stress and metabolism and contributes to autophagosome formation and fusion with the vacuole [50]. To assess whether *IMP1* might play a role in vesicular membrane trafficking and/ or autophagosome formation, we stained mycelia of WT and Δ*imp1* from 16 h cultures with both FM4-64 and MDC. **Fig 5B** shows that the overlap between FM4-64 labeled compartments and MDC labeled autophagic vacuoles was much less extensive in the Δ*imp1* mutant compared to WT, consistent with altered vesicular membrane trafficking in Δ*imp1*. Moreover, the number of MDC-labeled compartments was also reduced in Δ*imp1* compared to WT. This might be in line with observations in mammalian cells where the blocking of endocytosis and the delivery of plasma membrane to phagophores reduced autophagosomes by 30% [51]. Our results thus suggest that *IMP1* is required for membrane trafficking between endosomes and acidified autophagic vacuoles, and that *IMP1* might also be required for supplying endosomal membranes for phagophore expansion, which would be consistent with *IMP1* acting downstream of the TOR-mediated initiation and nucleation of phagophore assembly [49] during autophagy (**Fig 5C**).

### *IMP1* is required for V-ATPase assembly

We hypothesized that *IMP1* might be required for organelle acidification because it had a role in V-ATPase complex function. V-ATPases maintain organelle pH homeostasis by a rotary mechanism involving the V_1_ ATPase peripheral domain and the V_0_ proton translocating membrane domain [45, 52, 53]. Loss of function V-ATPase mutants in yeast display phenotypes similar to Δ*imp1*—including blocked autophagic flux—due to the loss of acidic vacuoles and other organelles [33, 35, 36, 44, 45]. The 493 amino acid Imp1 protein sequence shares some similarity (31% over 157 amino acids) with the protein expressed from the yeast locus YMR054W, also known as *STV1*, the isoform of *VPH1* encoding the subunit a of the V-ATPase V_0_ domain [54]. However, it should be noted that Stv1p shares 42% identity over 785 amino acids with MGG_03947, suggesting this allele is more likely to encode the *M*. *oryzae* subunit a of the V-ATPase V_0_ domain. Moreover, Imp1 does not align with annotated *M*. *oryzae* V-ATPase subunit sequences when BLASTed at Ensembl Fungi, including MGG_03947. Perhaps as a consequence, V-ATPase ATP hydrolysis activities, determined spectrophotometrically [55], were indistinguishable in vesicular membranes extracted from protoplasts derived from either Δ*imp1* or WT mycelia (**S4A Fig**) following growth in glucose-rich media [45, 53]. This indicates Imp1 is not likely a V_1_ subunit. However, differences between WT and Δ*imp1* were observed when we assayed V-ATPase proton pumping activity using the ΔpH probe acridine orange. Acridine orange (AO) quenching due to binding to the H+ charged inner vacuolar membranes is an indication of proton pump activity and vesicle acidification [55]. **S4B Fig** shows similar AO quenching in both WT and Δ*imp1* samples at the beginning of the assay, indicating V-ATPase was able to build a H+ gradient in Δ*imp1* like WT. However, the rates of absorbance quenching in Δ*imp1* diverged from WT at later time points, indicating Δ*imp1* was unable to maintain the pH gradient. Note that differences in AO absorbance quenching within Δ*imp1* samples when the V-ATPase inhibitor concanamycin A (ConA) was added suggests V-ATPase proton pumping activity is misregulated rather than abolished in this strain. Thus *IMP1* is not required for V-ATPase-dependent ATP hydrolysis, or proton pumping, but is required for maintaining the membrane charge gradient and H+ homeostasis.

**Fig 6:**
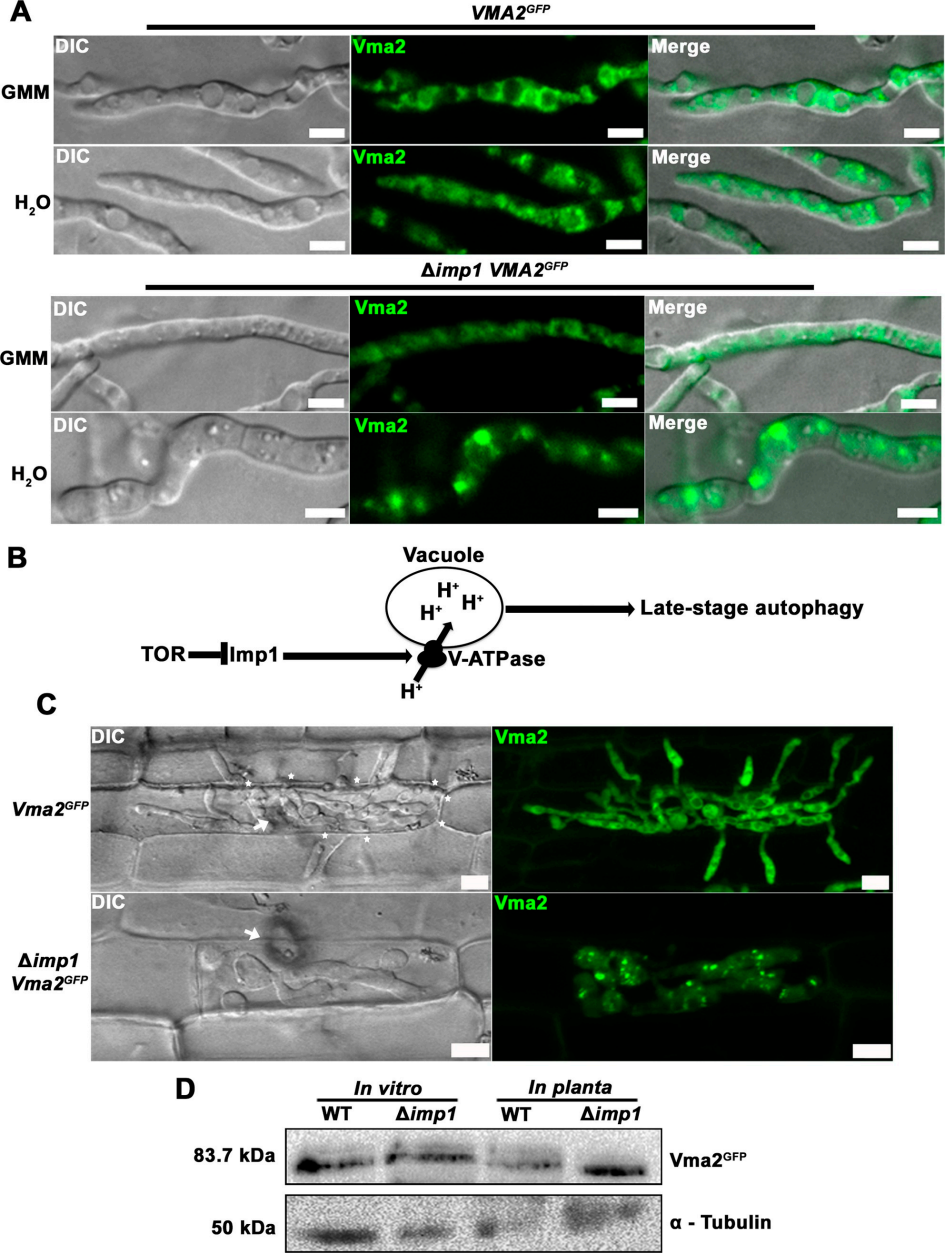
*IMP1* is required for V-ATPase assembly. (A) Subcellular localization of Vma2^GFP^, the fluorescently labeled V_1_ domain subunit B, was misregulated in Δ*imp1* vegetative mycelium compared to WT. GMM = glucose minimal media. Scale bars = 10 μm. (B) Model of the relationship between TOR, Imp1 and V-ATPase assembly and activity. (C) Vma2^GFP^ subcellular localization was examined in WT and Δ*imp1* strains during *in planta* growth at 44 hpi. Scale bars = 10 μm. White arrows indicate appressoria on the leaf sheath surface. (D) Immunoblot assessment of Vma2^GFP^ integrity using anti-GFP monoclonal antibodies. Proteins were extracted from vegetative mycelia (*left*) and infected rice leaf sheaths (*right*). α-tubulin was used as the loading control. Vegetative mycelia of the *VMA2*^*GFP*^ strain were grown in GMM for 16 hr. Infected leaf sheath were sampled at 44 hpi.

We next asked whether V-ATPase assembly (rather than enzymatic function) was perturbed in Δ*imp1* strains. Reversible assembly of V_0_ and V_1_ controls V-ATPase function [45, 53]. Multiple stresses induce V-ATPase assembly changes; the best characterized being the response to glucose [56]. In yeast, glucose starvation results in V_1_ disassembling from V_0_ (which remains membrane-bound), while adding glucose to carbon starved yeast cells promotes V_1_-V_0_ assembly [45, 57]. Although the signaling mechanism(s) involved are not well understood [56], the TOR pathway has been recently shown to control V-ATPase assembly in yeast via the downstream AGC kinase Sch9, which might act on Vph1 [45], the Stv1 isoform that shares some identity with Imp1. Like yeast, the *M*. *oryzae* genome [58] encodes homologues of the six V_0_ subunits a, c, c’, c”, d and e, and the eight V_1_ subunits A-H, suggesting V-ATPase function and dynamics might be conserved. To assess if *IMP1* influences V-ATPase assembly/ disassembly, we generated WT and Δ*imp1* strains expressing *VMA2* (encoding the V_1_ domain subunit B) fused with GFP. Our rationale was that unlike V_0_ subunits, which do not dissociate from the membrane, a V_1_ subunit would provide information on assembly dynamics. Strains were grown for 48 h in CM before switching to GMM or water for 3 h before visualizing Vma2^GFP^ localization. **Fig 6A** shows that in GMM (which might promote V_1_-V_0_ assembly), Vma2^GFP^ in WT hyphae localized around large vacuoles, whereas it was more uniformly dispersed throughout Δ*imp1* hyphae and did not outline vacuoles. Under starvation conditions, Vma2^GFP^ became more dispersed in the cytoplasm of WT and localized to punctate and tubular structures around vacuoles, but was internalized into large intracellular compartments in Δ*imp1* hyphae. These results suggest that the correct localization of Vma2^GFP^ requires *IMP1* under glucose starvation or TOR inactivation conditions, and *IMP1* might thus contribute, directly or indirectly, to glucose-dependent V-ATPase assembly/disassembly at vacuoles (**Fig 6B**).

When the results in **Fig 6** are considered alongside the data in **Figs 4** and **5**, we conclude that Imp1 integrates nutrient signaling via TOR with vacuole morphology, V-ATPase assembly and organelle acidification/ pH homeostasis, membrane vesicle trafficking and autophagy induction.

### *IMP1* is required for canonical V-ATPase-dependent vacuole functions

Additional evidence that *IMP1* is required for vacuole function is shown in **S5 Fig**. Vacuoles regulate cytosolic ion concentrations (including K^+^), and play major roles in metal homeostasis and metal detoxification [59]. The loss of vacuole function associated with yeast V-ATPase mutants resulted in sensitivity to high concentrations of Ca^2+^ and heavy metal cations including Zn^2+^ [35–37, 44]. To test whether ion homeostasis and/ or metal detoxification was altered in Δ*imp1* strains compared to WT, we grew both strains on GMM with or without elevated levels of K^+^, Ca^2+^ and Zn^2+^ (**S5A Fig**). Δ*imp1* radial growth was slightly reduced on all ion-treated media compared to WT and untreated GMM, with the largest reduction in growth observed on Zn^2+^ media, suggesting partially impaired ion and metal homeostatic and detoxification vacuolar functions in Δ*imp1* strains.

Functioning vacuoles are essential for yeast growth under elevated temperatures [60, 61]. We grew WT and Δ*imp1* strains on media incubated above and below the optimum temperature for *M*. *oryzae* growth (26 ^o^C). As predicted, Δ*imp1* responded differently to elevated incubation temperatures than WT but strikingly, the Δ*imp1* mutant was more tolerant, not sensitive, to growth at high temperatures compared to WT (**S5B Fig**). This is consistent with the notion that vacuole functions can be tailored to species or lifestyle-specific needs [38].

Unlike yeast V-ATPase mutants [35], Δ*imp1* was not more sensitive to oxidants compared to WT. Rather, when grown under hypoxic conditions, Δ*imp1*—compared to Δ*imp1* under normoxic conditions and WT under both conditions—elaborated thick aerial hyphae (**S5C Fig**), indicating altered responses to low oxygen that could result from altered vacuole function.

A canonical outcome of yeast non-acidic vacuole mutants resulting from V-ATPase disruption and the loss of H^+^ pumping is the inability to grow at high pH [44]. In *M*. *oryzae*, we did not observe differences in radial growth between Δ*imp1* and WT when grown on high pH media (**S5D Fig**), but Δ*imp1* sporulation rates were greatly reduced compared to WT at high pH (**S5E Fig**). Unexpectedly, Δ*imp1* was more tolerant than WT on low pH media with regards to radial growth (**S5D Fig**).

We conclude that canonical vacuolar processes that rely on V-ATPase activity—such as metal and ion homeostasis, temperature, oxygen and pH responses—are affected by the loss of *IMP1*. However, some outcomes of Δ*imp1* are less severe than those observed for yeast V-ATPase mutants while other outcomes are altered, perhaps reflecting changes in lifestyle. These outcomes also differ from those previously described for the *M*. *oryzae* Δ*vma11* mutant disrupted for the gene encoding the V-ATPase subunit c’ of V_0_ [62]. The Δ*vma11* mutant displayed reduced organelle acidification like Δ*imp1* but was drastically impaired for growth on untreated media, almost entirely abolished for sporulation (and hence appressorium formation), and was more sensitive to Zn^2+^ than we report here for Δ*imp1* [62]. The affect of Δ*vma11* on autophagy was not assessed. Compared to Δ*vma11*, Δ*imp1* thus has a partial loss of V-ATPase phenotype. This partial *vma*^*-*^ phenotype was similar to that recently reported in yeast for the *sch9*Δ mutant, which led to the suggestion that the Sch9 branch of the TOR signaling pathway might regulate V-ATPase activity [45].

### Inhibiting vacuole function does not confer rapamycin resistance in *M*. *oryzae*

A recent study in yeast showed that the reactivation of TOR signaling following starvation and in response to amino acid uptake required the influx of protons rather than direct stimulation by amino acids themselves [22]. A cell membrane proton pump, Pma1, was shown to maintain the cytosolic proton gradient and was required for activating TOR signaling. Inhibiting V-ATPase activity with ConA perturbed cytosolic pH and activated TOR signaling during growth under nitrogen-poor conditions [22]. Although the results in **Fig 2** suggested TOR kinase was not constitutively activated by the loss of Δ*imp1*, we asked if inhibiting V-ATPase function might nonetheless affect TOR signaling. To assess this, we grew WT and Δ*imp1* strains on media with and without rapamycin, with and without a sub-lethal concentration of ConA, and with both rapamycin and ConA. **S6 Fig** shows that ConA treatment does not confer rapamycin resistance to WT. Thus, while we acknowledge that V-ATPase inhibition might, like in yeast, affect TOR activity under certain nitrogen growth regimes, this is not likely the case under our test conditions. These results reinforce our conclusion that the loss of *IMP1* does not result in TOR activation, rule out the loss of vacuole function as the source of rapamycin resistance, and are consistent with *IMP1* functioning downstream of TOR. Conversely, these results indicate that in WT, rapamycin inhibition of TOR during growth on glucose-rich media does not require a functioning vacuole (**S6 Fig**), placing V-ATPase (and thus *IMP1*) downstream of TOR in the TOR-autophagy signaling axis. Finally, ConA treatment alone restricted Δ*imp1* growth (**S6 Fig**), consistent with our conclusion that the loss of *IMP1* confers a partial *vma*^*-*^ phenotype.

### *IMP1* controls Vma2^GFP^ subcellular localization *in planta*

Armed with the knowledge that *IMP1* encodes a downstream TOR signaling component required for vacuole function, membrane trafficking and the induction of autophagy, we turned our attention to understanding the role of *IMP1* in rice infection. We hypothesized that *IMP1* likely plays similar biological roles during growth *in planta* compared to axenic growth. This was first suggested by the observation that Imp1^GFP^ localizes to vacuoles during both axenic and *in planta* growth (**Fig 3E**). To determine if the loss of *IMP1* affected vacuole function *in planta*, we attempted to stain acidified compartments with quinacrine during rice leaf sheath infections by WT and Δ*imp1* strains, but this was unsuccessful, likely due to the difficulty of some exogenous treatments (but not all, see below) in crossing the plant cell wall and plasma membrane, the EIHM, the fungal cell wall and plasma membrane, and into the *M*. *oryzae* cell. We turned instead to confocal microscopy, which revealed how by 44 hpi, Vma2^GFP^ localized around large vacuoles in WT IH, but was mislocalized into punctate structures in Δ*imp1* IH (**Fig 6C**), which also lacked obvious vacuoles. This indicated *IMP1* maintains its roles during plant infection in ensuring correct Vma2^GFP^ cellular localization, V-ATPase assembly dynamics and vacuole morphology. **Fig 6D** shows that Vma2^GFP^ is detected at the expected size in immunoblots using anti-GFP antibodies against proteins extracted from both mycelia and infected rice leaf sheaths, indicating that the Vma2^GFP^ protein is intact and not processed in Δ*imp1* strains.

In contrast to the *M*. *oryzae* Δ*vma11* mutant that was unable to establish any IH in host cells [62], Δ*imp1* was able to elaborate IH in the first infected rice cells (**S2D Fig**), and Vma2^GFP^ accumulation indicated that although Δ*imp1* IH were growth-inhibited at 44 hpi (**Fig 6C**), Δ*imp1* strains were not dead. This again illustrates how the loss of *IMP1* results in only a partial *vma*^*-*^ phenotype.

### *IMP1* is required for maintaining biotrophic interface membrane integrity

**Fig 7:**
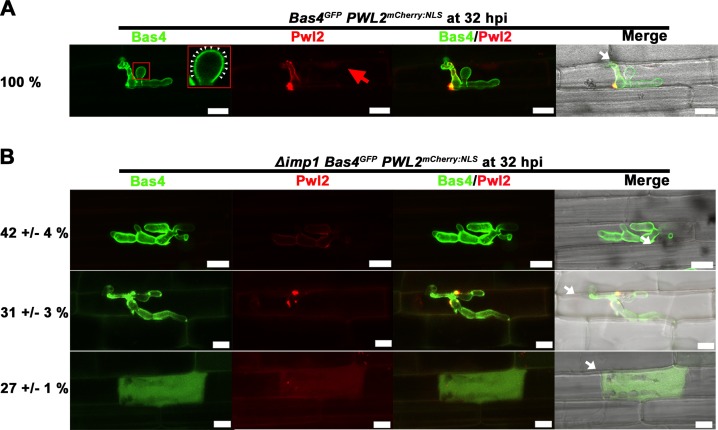
*IMP1* maintains biotrophic interface integrity. WT (A) and Δ*imp1* (B) strains expressing the fluorescently labeled apoplastic effector Bas4^GFP^ and the fluorescent BIC-accumulating cytoplasmic effector Pwl2^mCherry:NLS^ at 32 hpi. White arrows indicate appressoria on the leaf sheath surface and red arrow indicates the faint enrichment of Pwl2^mCherry:NLS^ in an adjacent rice nucleus. Arrowheads highlight Bas4^GFP^ in the apoplast. Scale bars = 10 μm. Percentages are mean values +/- s.d. of each representative image obtained from observing 100 infected rice cells per strain, repeated in triplicate.

**Fig 8:**
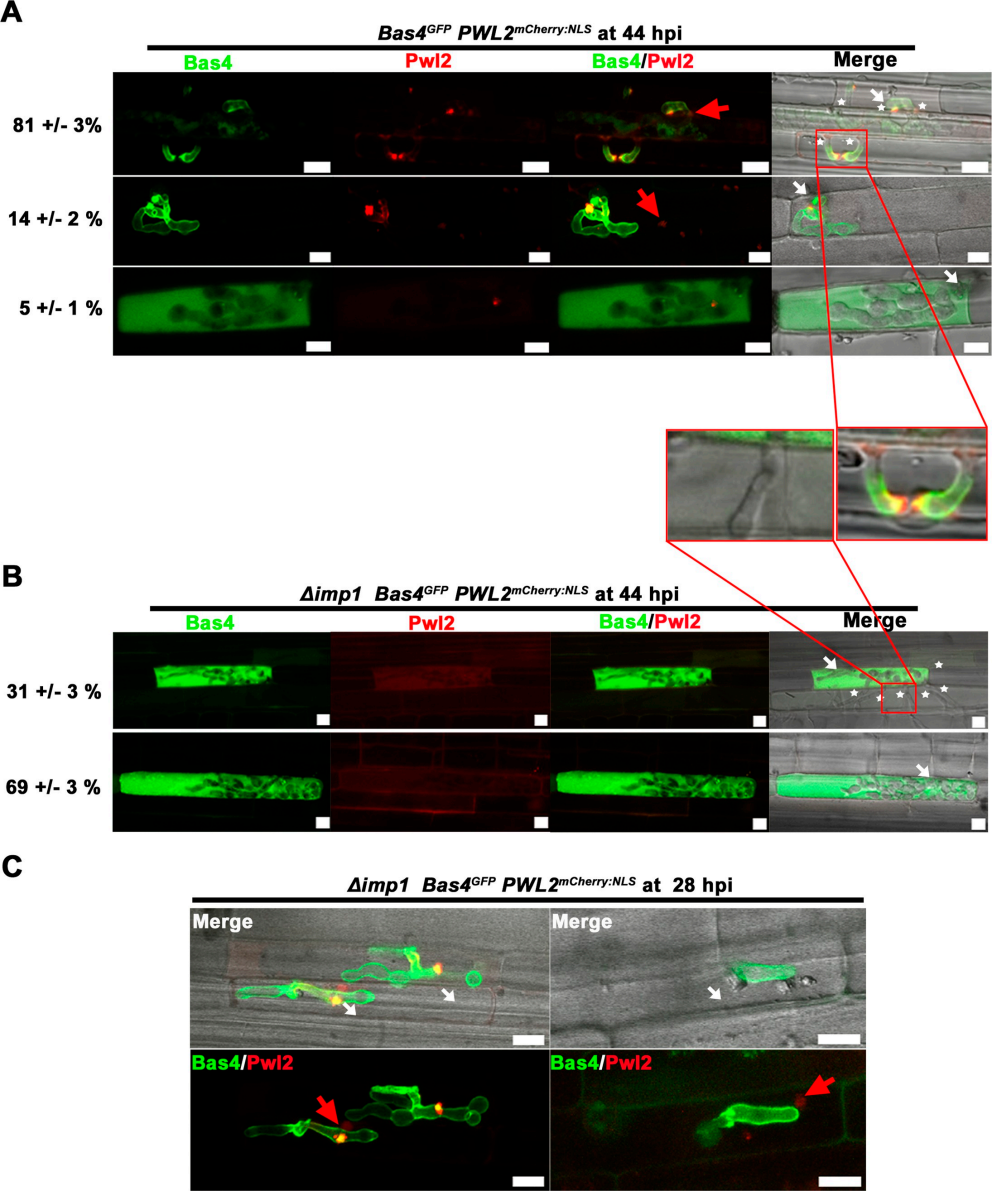
*IMP1* is required for biotrophic interface longevity. WT (A) and Δ*imp1* (B) strains expressing the fluorescently labeled apoplastic effector Bas4^GFP^ and the fluorescent BIC-accumulating cytoplasmic effector Pwl2^mCherry:NLS^ at 44 hpi. Stars indicate emerging IH in adjacent cells. Arrows indicate appressoria on the leaf sheath surface. Scale bars = 10 μm. Percentages are mean values +/- s.d. of each representative image obtained from observing 100 infected rice cells per strain, repeated in triplicate. (C) Representative images of Δ*imp1* expressing Bas4^GFP^ and Pwl2^mCherry:NLS^ and infecting rice cells at 28 hpi. Small white arrows indicate appressoria on the leaf sheath surface. Large red arrow indicates accumulation of Pwl2^mCherry:NLS^ in an adjacent rice nucleus. Scale bars = 10 μm.

Using fluorescent effectors as molecular probes, we next deduced that the loss of *IMP1* led to stochastic erosion of *M*. *oryzae*-rice biotrophic interfaces (both BIC and EIHM) during fungal growth in rice cells. We generated Δ*imp1* strains expressing fluorescently labeled effectors by introducing the vector pBV591 into Δ*imp1*. pBV591 carries genes encoding the apoplastic effector Bas4 fused to GFP, and the cytoplasmic effector Pwl2 fused to mCherry and a rice nuclear localization signal (NLS) [7]. In an otherwise WT strain, Bas4^GFP^ outlines IH while Pwl2^mCherry:NLS^ accumulates in the BIC before translocating into rice cells where it artificially concentrates in the rice nucleus [7, 11]. Two independent transformants of Δ*imp1* expressing Bas4^GFP^ and Pwl2^mCherry:NLS^ were characterized, and *IMP1* was also deleted from our pBV591-carrying strain derived from WT [11]. All Δ*imp1* strains expressing Bas4^GFP^ and Pwl2^mCherry:NLS^ produced similar results, with representative images shown in **Figs 7** and **8**.

**Fig 7A** shows how, by 36 hpi, all WT infected rice cells carried IH outlined by Bas4^GFP^ in the apoplast and accumulating Pwl2^mCherry:NLS^ in a single focal BIC, as previously described [7, 11]. Faint accumulation in an adjacent rice nucleus is observed. In contrast, **Fig 7B** shows representative images demonstrating how, by 36 hpi, 42% of Δ*imp1-*infected rice cells carried Δ*imp1* IH outlined by Bas4^GFP^ like WT but with no observable BICs; 31% carried Δ*imp1* IH outlined with Bas4^GFP^ but with multiple Pwl2^mCherry:NLS^-accumulating BIC foci; 27% carried Δ*imp1* IH with no observable BICs and with Bas4^GFP^ expelled into the rice cytoplasm.

By 44 hpi, > 80% of primary WT infected rice cells carried IH that had spread into neighbouring cells, where they had produced additional BICs at emerging IH tips, and retained Bas4^GFP^ in the apoplast around IH (**Fig 8A**), as previously described [7]. In contrast, in 100% of cases, BICs were not detected in Δ*imp1* IH by 44 hpi (**Fig 8B**) despite Δ*imp1* expressing *PWL2* (and *BAS4*) *in planta* at similar levels to WT (**S7 Fig**). Bas4^GFP^ was expelled into the cytoplasm of the first infected cell in 100% of cases (**Fig 8B**). In 31% of primary infected cells, Bas4^GFP^ expulsion occurred despite Δ*imp1* IH moving to adjacent cells, although newly emerging Δ*imp1* IH were not outlined with Bas4^GFP^ and BICs were not observed (**Fig 8B**).

Leakage of Bas4^GFP^ into the cytoplasm of WT infected rice cells has been observed in rare cases (1%) and attributed to the loss of EIHM integrity [63]. In our hands and at the time points we used, we observed this phenomenon in 5% of cells infected with WT by 44 hpi (**Fig 8A**), and we attribute most of it to mishandling and damage to the leaf sheaths prior to microscopy. However, the loss of EIHM integrity occurred in only a minority of those primary infected host cells where WT IH had failed to thrive and grow to adjacent cells, and an additional 14% of infected rice cells carried WT IH that had failed to spread to adjacent cells but retained apoplastic Bas4^GFP^ and a BIC. Because the incidences of BIC loss and Bas4^GFP^ release into rice cytoplasm was thus considerably higher in rice cells infected with Δ*imp1* strains than WT—and increased with time—we conclude that *IMP1* is required to prevent the erosion of biotrophic interfacial membrane integrity (both BIC and EIHM) as biotrophy progresses. To our knowledge, this is the first time a fungal gene required for biotrophic interface function and longevity has been described in any system.

To support our conclusion that *IMP1* contributes to maintaining biotrophic interfacial membrane integrity during fungal growth in rice cells, we hypothesized that early infection time points would capture Δ*imp1* IH with an intact BIC (in addition to Bas4^GFP^ outlining IH). Due to the asynchronous nature of the infection process, time points before 32 hpi (in our hands) are not suitable for the statistical analyses of IH development. Nonetheless, at 28 hpi, we discerned several instances of BICs in Δ*imp1* IH (**Fig 8C** *left*) as well as examples where the BIC was absent, but Pwl2^mCherry:NLS^ had accumulated in adjacent rice nuclei (**Fig 8C** *right*). However, we did not detect Pwl2^mCherry:NLS^ in nuclei of neighboring cells ahead of IH invasion, suggesting either that Pwl2^mCherry:NLS^ deployment by Δ*imp1* was decreasing by 28 hpi compared to WT, or that Pwl2^mCherry:NLS^ never accumulated to levels sufficient to be observed in neighboring nuclei. Taken together, these results suggest Δ*imp1* IH produce a Pwl2-secreting BIC, and retain a Bas4-accumulating apoplast, during very early infection, but first the BIC, and then the EIHM, are lost as biotrophy progresses. Because Vma2^GFP^ was visualized in Δ*imp1* IH at 44 hpi (**Fig 6C**), this observed membrane senescence is specific to biotrophic interfaces and is not accompanied by general cellular senescence, despite biotrophic growth being attenuated.

### The TOR-*IMP1*-autophagy signaling axis modulates biotrophic interface longevity

We next asked whether poor biotrophic growth of Δ*imp1* resulted from the stochastic loss of biotrophic membranes over time, indicating *IMP1* was a direct regulator of interface integrity during biotrophic growth, or whether the loss of biotrophic growth by Δ*imp1* compromised biotrophic interfacial membrane integrity, perhaps due to an early transition to necrotrophy. In other words, was the loss of virulence in Δ*imp1* the cause rather than the effect of the loss of BIC and EIHM membrane integrity? To address this question, we sought more understanding on the nature of the biotrophic interface by determining its persistence in WT, we assessed whether TOR-autophagy signaling *in planta* controlled biotrophic interface longevity, and we explored whether the loss of membrane integrity could be reversed. In this manner, we discovered that Imp1-dependent autophagy induction controls both the longevity of biotrophic interfaces over time, and biotrophic cell-to-cell movement.

#### The biotrophic interface persists through the onset of necrotrophy in WT

In order to establish how long the biotrophic interface persisted in WT, we examined infected rice cells at 72 hpi at the onset of necrosis when, in our hands, WT infected rice cells begin accumulating visible compounds likely due to cell death. **S8 Fig** shows that at 72 hpi, WT BICs were still visible and Bas4^GFP^ outlined WT IH- although some Bas4^GFP^ was also accumulating in the fungal cytoplasm. However, no Bas4^GFP^ was observed accumulating in the rice cell. Thus, in WT the biotrophic interface can persist through the onset of the transition to necrotrophy.

#### Rice defenses are not elicited by Δ*imp1* at early infection stages

By 72 hpi, rice cells infected with Δ*imp1* elicited a much stronger, visible reaction compared to WT, and Δ*imp1* IH was not observed spreading beyond the second infected cell (**S8 Fig**). Some Bas4^GFP^ accumulated within IH, which might be indicative of perturbed Bas4^GFP^ secretion at this late time point. We asked whether Δ*imp1* might be impaired in suppressing plant defenses during early infection, which would result in restricted biotrophic growth and might lead to the observed loss of biotrophic membrane integrity. In previous studies [9, 11], mutant strains including Δ*sir2* that are unable to suppress plant defenses elicit strong responses including visible occlusions, elevated plant defense gene expression and H_2_O_2_ accumulation. No visible occlusions were observed in Δ*imp1* infected rice cells at 44 hpi when Δ*imp1* IH had lost BICs and Bas4^GFP^ was expelled into the rice cytoplasm in 100% of infected cells (**Fig 8**). Furthermore, pathogenesis-related (*PR*) plant defense gene expression was not elevated in Δ*imp1* infected rice cells compared to WT at 44 hpi (**S9A Fig**), and compared to Δ*sir2* infected cells, H_2_O_2_ was not detected by 3,3′-diaminobenzidine (DAB) staining at 32 hpi (**S9B Fig**). Thus, plant defenses are not elevated in rice cells infected with the Δ*imp1* mutant during early infection and are not likely the cause of the loss of biotrophic membrane integrity. Therefore, Δ*imp1* successfully suppresses host defenses prior to the loss of biotrophic interface membrane integrity and the subsequent misdeployment of effectors.

#### Loss of *IMP1* confers rapamycin resistance *in planta*

To determine if Imp1 contributed to biotrophic interface longevity as part of the TOR-autophagy signaling pathway, by using effector probes, we first confirmed that *IMP1* was involved in TOR signaling and sensitivity to rapamycin during growth *in planta*. To achieve this, we developed a method to apply exogenous treatments to rice leaf sheaths after rice cell infection had commenced. An untreated spore suspension was added to the hollows of rice leaf sheaths at 0 hpi, as per our normal protocol. At 24 hpi (in the case of rapamycin, 36 hpi for other treatments, see below), the spore suspension was removed from leaf sheath hollows and replaced with a solution containing 10 μM rapamycin dissolved in water. The leaf sheaths were returned to the incubator until 44 hpi, when they were visualized by laser scanning confocal microscopy. **S10 Fig** shows that by 44 hpi, untreated WT had filled the first cell and moved to adjacent cells, as expected, but treatment with rapamycin at 24 hpi severely inhibited WT growth in the first infected cell. This result is consistent with our previous work suggesting TOR signaling is active during early infection in order to promote biotrophy and mitosis [12]. Following rapamycin treatment, a BIC and apoplastic Bas4^GFP^ were evident in WT, although fluorescence was weak compared to the untreated WT control. This implies that inactivating TOR signaling and attenuating biotrophic growth did not impair interface membrane integrity, however prolonged rapamycin exposure is likely toxic to WT and might affect protein accumulation or production. In contrast to WT, rapamycin treatment had no effect on Δ*imp1* physiology compared to the Δ*imp1* untreated control, and Bas4^GFP^ was expelled into infected cells in high amounts under both conditions (**S10 Fig**). Furthermore, Δ*imp1* growth, although reduced compared to untreated WT IH, was more extensive than rapamycin treated WT IH, suggesting Δ*imp*, like under axenic growth conditions, was insensitive to rapamycin exposure *in planta*. Our conclusions were two-fold, firstly that *IMP1* acts downstream of TOR *in planta*; and secondly that, because rice TOR does not respond to rapamycin [64, 65], at least some exogenous treatments are capable of affecting fungal physiology in host cells without eliciting confounding affects from plant targets.

#### Autophagy is required for maintaining biotrophic interface integrity

**Fig 9:**
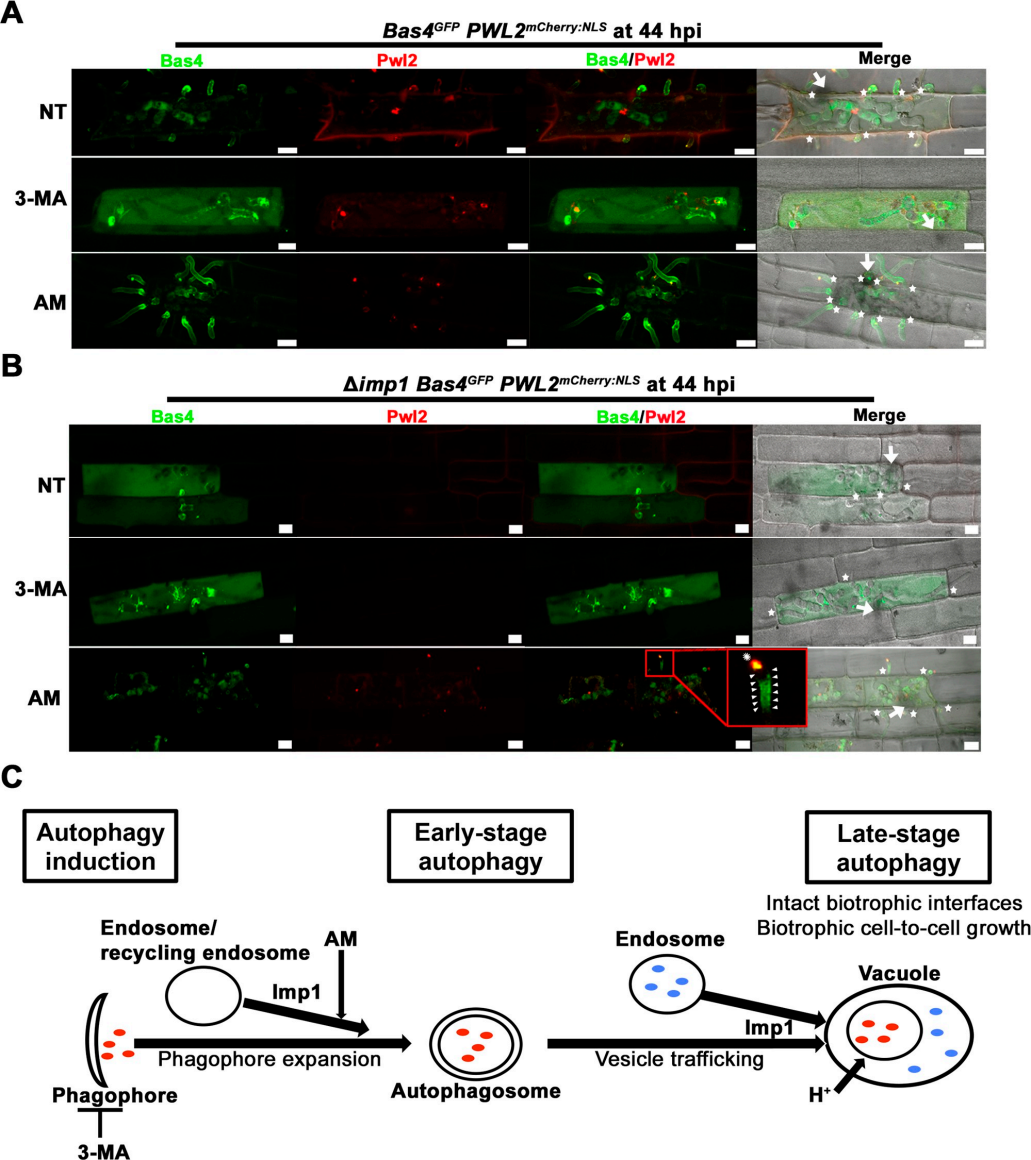
Autophagy is required for biotrophic interface membrane integrity and cell-to-cell movement. Leaf sheaths infected with WT (A) or Δ*imp1* (B) strains expressing the fluorescently labeled apoplastic effector Bas4^GFP^ and the fluorescent BIC-accumulating cytoplasmic effector Pwl2^mCherry:NLS^ were treated with the autophagy induction inhibitor 5 mM 3-methyladenine (3-MA) or the autophagy stimulator 2 μM Amiodarone Hydrochloride (AM) at 36 hpi and viewed at 44 hpi. (A,B) Stars indicate emerging IH in adjacent cells. Arrows indicate appressoria on the leaf surface. Scale bars = 10 μm. NT = no treatment. Proportion of infected rice cells represented by these images are shown in **S1 Table.** *Zoom box*, arrowheads highlight how Bas4^GFP^ outlines IH, asterisk highlights the reconstituted BIC. (C) Model based on 3-MA and AM treatments showing relationship of Imp1 to autophagy, biotrophic interface membrane integrity and cell-to-cell growth. Because AM induces autophagy by increasing autophagosome formation, it must act downstream of the proposed role of Imp1 in facilitating phagophore expansion.

We next tested if autophagy induction (impaired in Δ*imp1*) was important for maintaining biotrophic interface integrity. We treated WT infected leaf sheaths at 36 hpi with the phosphatidylinositol 3-kinase inhibitor 3-methyladenine (3-MA), which inhibits autophagy induction and autophagosome formation by blocking phagophore initiation [66]. Importantly, 3-MA treatment recapitulated the Δ*imp1* phenotype in WT when viewed at 44 hpi (**Fig 9A**). This included releasing Bas4^GFP^ into rice cells, the generation of multiple, small BIC foci, and abolishing biotrophic cell-to-cell growth. For Δ*imp1*, 3-MA had no additional effects on physiology compared to the untreated control (**Fig 9B**).

Strikingly, treating Δ*imp1* infected leaf sheaths at 36 hpi with the TOR-independent autophagy activator AM remediated membrane integrity and resulted, by 44 hpi, in emerging IH in adjacent cells carrying reconstituted tip BICs like WT and retaining Bas4^GFP^ in the apoplast (**Fig 9B**). These results highlight the previously unknown importance of fungal autophagy in maintaining the plant-fungus biotrophic interface. Because AM was added at 36 hpi when Δ*imp1* is losing biotrophic interface integrity (**Fig 7B**), BIC and EIHM reconstitution by 44 hpi in the majority of AM-treated Δ*imp1* infected cells (**S1 Table**) provides substantial evidence that the impaired growth of untreated Δ*imp1* does not result from an early transition to necrotrophy, which would be irreversible. Remediation by AM suggests instead that in Δ*imp1*, the loss of biotrophic membrane integrity results from perturbed TOR-Imp1-autophagy signaling and is not associated with the loss of biotrophy *per se*, demonstrating that impaired biotrophic growth by Δ*imp1* is a consequence not a cause of the loss biotrophic interface integrity. Remediation of Δ*imp1* by AM also indicates that the major, if not only, role of *IMP1* during biotrophy is in the TOR-autophagy signaling branch.

#### Stimulating autophagy increases biotrophic cell-to-cell movement rates in WT and Δ*imp1*

In addition to remediating biotrophic interface integrity, the number of individual Δ*imp1* IH (with tip BICs) emerging into neighbouring cells from the primary infected cell was significantly increased following AM treatment compared to untreated cells (**S11 Fig**). Adding AM to WT infected rice leaf sheaths at 36 hpi did not affect fungal development or effector secretion compared to untreated controls when viewed at 44 hpi (**Fig 9A**), but the number of individual hyphae moving into cells adjacent to the first infected cell was also significantly increased compared to untreated controls (**Fig 9A** and **S11 Fig**). Considering that active TOR signaling is required for very early biotrophy [12], these results suggest that in WT, TOR signaling and autophagy is dynamic during growth in the first infected rice cell. Together, our results suggest that *IMP1*-dependent autophagy induction in response to TOR signaling during infection is required for maintaining EIHM and BIC integrity and for promoting biotrophic cell-to-cell growth (**Fig 9C**).

### Imp1-dependent membrane trafficking following autophagy induction maintains biotrophic interface integrity

#### Vacuole acidity and late-stage autophagy is not required for maintaining biotrophic interface integrity

**Fig 10:**
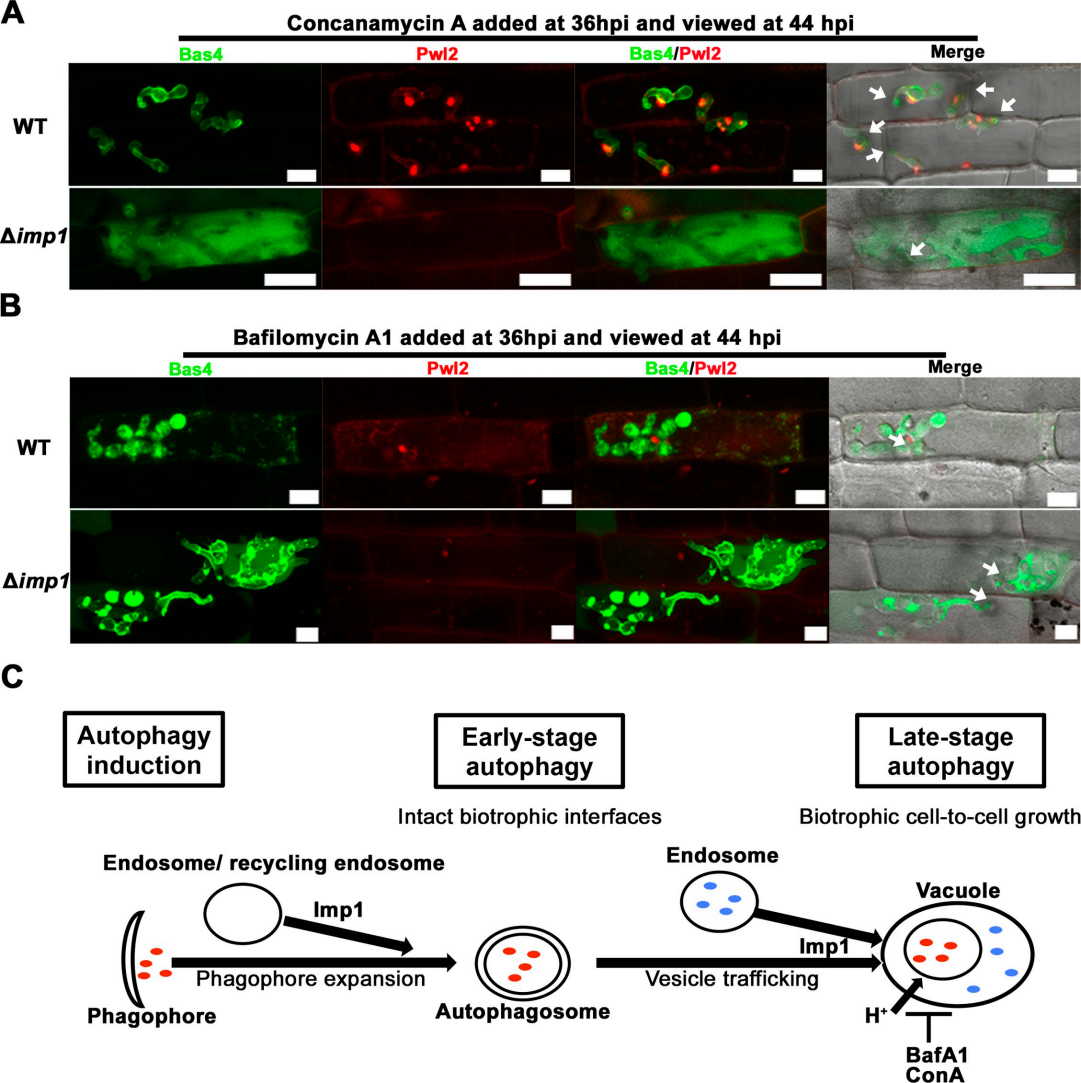
V-ATPase function is not required for biotrophic interfacial membrane integrity. Leaf sheaths infected with the indicated strains were treated with (A) 10μM concanamycin A (ConA) or (B) 1 μM bafilomycin A1 (BafA1) at 36 hpi and viewed at 44 hpi. (A,B) Arrows indicate appressoria on the leaf surface. Scale bars = 10 μm. NT = no treatment. Proportion of infected rice cells represented by these images are shown in **S1 Table.** (C) BafA1 and ConA treatments suggest V-ATPase and vacuole function is required for cell-to-cell movement but not for maintaining biotrophic interfaces, which therefore must be dependent on an earlier stage of autophagy.

We next asked which *IMP1*-dependent processes might be required for maintaining biotrophic interface membrane integrity. First, we considered whether vacuole acidification and late-stage autophagy was responsible for maintaining biotrophic interface integrity and/ or effector secretion. The macrolides concanamycin A (ConA) and bafilomycin A1 (BafA1) are specific V-ATPase inhibitors that target subunit c of the V_0_ domain and block the swiveling action of the H^+^ pump, thereby dissipating the lysosome/ vacuole pH gradient and preventing late-stage autophagy by inhibiting proteolytic degradation of autophagic bodies [33, 53, 66, 67]. Both treatments can induce apoptosis in some systems. In the *Drosophila* fat body, BafA1 additionally disrupts lysosome fusion to autophagosomes and endosomes by a pH-independent mechanism involving the Ca^2+^ pump SERCA [33]. **Fig 10A** shows that ConA treatment of WT-infected rice leaf sheaths, when added at 36 hpi and viewed at 44 hpi, severely restricted WT biotrophic growth and eliminated IH branching in the first infected cell. However, BICs were evident and Bas4^GFP^ outlined IH indicating interface integrity was not disrupted by V-ATPase inhibition in WT. In Δ*imp1*, ConA treatment did not further affect the loss of interface integrity (**Fig 10A**), nor did it remediate it, although Δ*imp1* IH growth was not as restricted as ConA treated WT IH, suggesting a modicum of tolerance to ConA.

**Fig 10B** shows that BafA1 treatment also inhibited WT growth in rice cells when applied at 36 hpi and viewed at 44 hpi, and again biotrophic membrane integrity was not lost: Pwl2^mCherry:NLS^ accumulation in the WT BIC was not affected by BafA1 treatment, and Bas4^GFP^ accumulated on IH and was not evident in the infected rice cell. Some Bas4^GFP^ accumulated in internal compartments in WT IH following BafA1 treatment, suggesting BafA1 treatment (but not ConA) might inhibit the conventional ER-to-Golgi secretion of apoplastic effectors. This might be consistent with BafA1 (but not ConA) affecting vesicle fusion independently of V-ATPase-dependent vacuole acidity [33], although differences between the two treatments might also reflect different efficacies in penetrating infected rice leaf sheaths and acting on fungal targets, or some other effect of BafA1 on effector deployment.

Like ConA, BafA1 treatment did not remediate biotrophic interface integrity in Δ*imp1* IH (**Fig 10B**). BICs were not evident and Bas4^GFP^ was released into rice cells. However, Bas4^GFP^ also accumulated in internal compartments and the expulsion of Bas4^GFP^ from Δ*imp1* IH was reduced after BafA1 treatment compared to untreated Δ*imp1*.

Taken together, ConA and BafA1 treatments show that inhibiting V-ATPase activity in WT (and thus impairing V-ATPase assembly, vacuole acidification and late-stage autophagy) attenuates biotrophic growth but does not affect biotrophic interfacial membrane integrity. We conclude that the poor growth of Δ*imp1* in rice cells might result from misregulated V-ATPase assembly and the loss of vacuole acidification and late-stage autophagy, but this does not account for the observed stochastic loss of biotrophic interface integrity, indicating that the role of Imp1 in biotrophic membrane integrity lies upstream of its role in vacuole function (**Fig 10C**).

#### Imp1 membrane localization depends on autophagy induction but not V-ATPase activity

From the *in planta* study of Imp1^GFP^ localization following V-ATPase and autophagy inhibition, we next garnered evidence that Imp1 functions downstream of autophagy induction and upstream of vacuole acidification by acting in membrane trafficking. **S12 Fig** and **S2 Table** shows that BafA1 and ConA treatments, which inhibit V-ATPase activity and impair vacuole function, did not affect Imp1^GFP^ localizing to the vacuole or to IH, even though biotrophic growth was severely attenuated. These results indicate that Imp1^GFP^ localization to both the vacuole and IH occurs upstream or independently of V-ATPase activity, vacuole acidity and late-stage autophagy.

**Fig 11:**
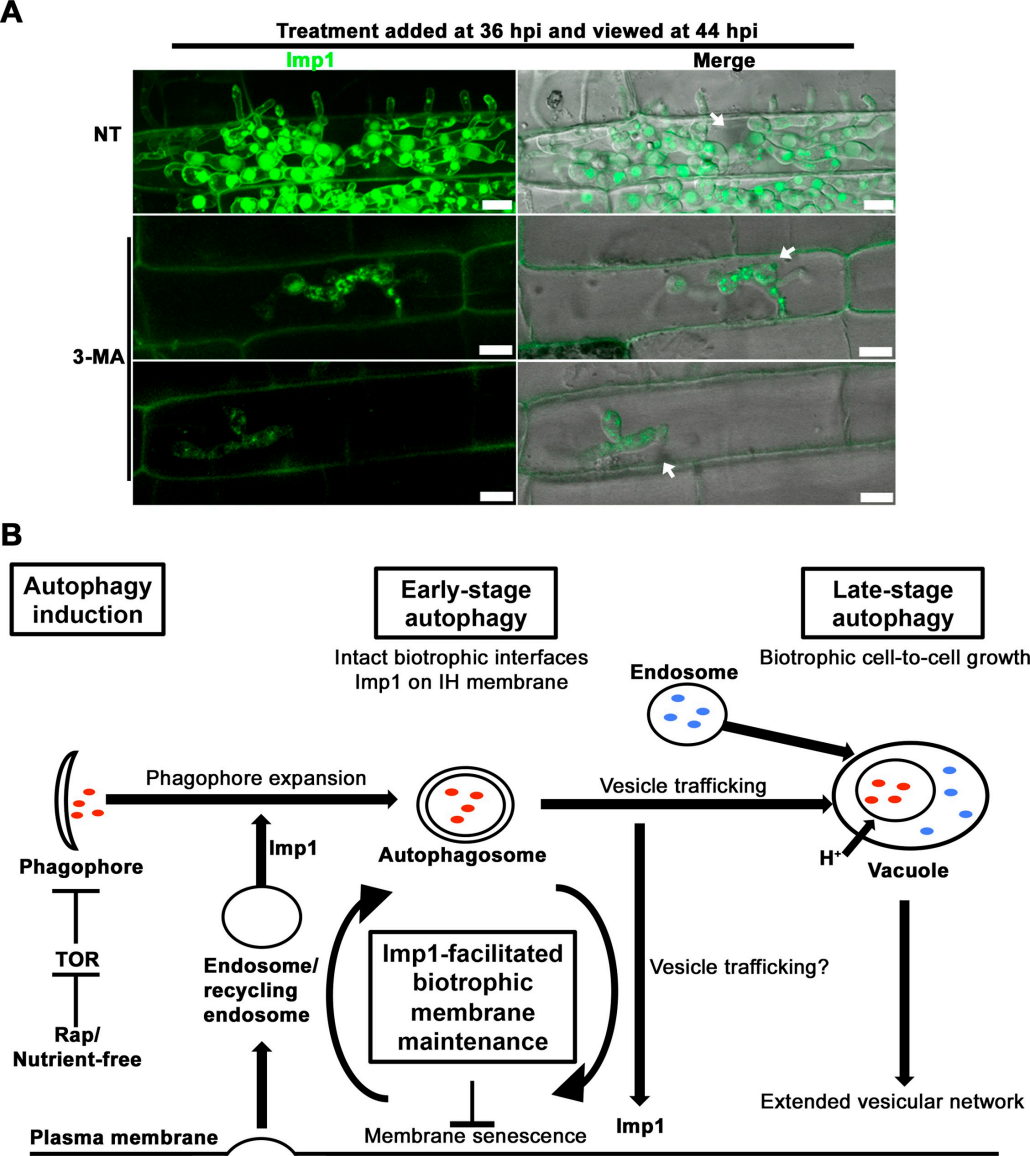
Imp1^GFP^ membrane localization is dependent on autophagy induction during growth in rice cells. (A) Imp1^GFP^ localization on the IH membrane and vacuole morphology during growth in rice cells is dependent on autophagy induction. Leaf sheaths infected with the Δ*imp1 IMP1*^*GFP*^ complementation strain expressing Imp1^GFP^ were treated with 5 mM 3-methyladenine (3-MA) at 36 hpi and viewed at 44 hpi. Scale bars = 10 μm, arrows indicate appressoria on the leaf sheath surface. NT = no treatment. Proportion of infected rice cells represented by these images are shown in **S2 Table.** (B) Model showing the TOR-dependent role of Imp1 and autophagy in facilitating the lifespan of the BIC and EIHM, collectively referred to as the biotrophic interface. Although Imp1 is required for autophagy induction and is involved in V-ATPase function and the fusion of autophagosomes and endosomes to vacuoles, pharmacological evidence situates the role of Imp1 in biotrophic interface maintenance and effector deployment upstream of V-ATPase activity and downstream of phagophore initiation. Also, because 1) the number of autophagosomes are decreased in Δ*imp1*; 2) endosomes can contribute membranes to phagosomes, and 3) because AM elevates early autophagy by increasing autophagosome number; we suggest that Imp1 acts by facilitating the contribution of endosomal membranes, originating at the plasma membrane, to phagophore expansion. Furthermore, 3-MA inhibition of autophagy induction prevented Imp1 localization on IH membranes, suggesting Imp1 might facilitate membrane recycling through the wider vesicular network or—because ConA and BafA1 treatment did not prevent Imp1 localizing on IH membranes—via a vacuole-independent route. Loss of Imp1 would reduce the efficiency of this membrane recycling process, leading to stochastic senescence of the biotrophic membrane over time. We thus propose that in response to autophagy initiation, Imp1 has two distinct roles: in vacuole function and late stage autophagy to optimize biotrophic growth, and in facilitating biotrophic interface longevity by mediating membrane sourcing during phagophore expansion and autophagosome formation.

3-MA treatment led to the fragmentation of Imp1^GFP^-carrying vacuoles, indicating Imp1 functions downstream of autophagy induction (**Fig 11A**). Inhibiting autophagy induction with 3-MA also led to the loss of Imp1^GFP^ from IH membranes (**Fig 11A**). Imp1^GFP^ localization on IH is thus dependent on autophagy induction (**Fig 11A**) but independent of V-ATPase activity and late stage autophagy (**S12 Fig**). Because autophagy induction but not vacuole function is also required for biotrophic membrane integrity, we propose that Imp1 prevents early biotrophic interface senescence by mediating membrane sourcing via endosomal membrane trafficking and plasma membrane recycling in a mechanism involving early autophagy induction and which is governed by TOR. All our findings considered together fit the model in **Fig 11B**.

## Discussion

TOR signaling status has recently emerged as an important factor governing rice infection by the blast fungus *M*. *oryzae*: inactive TOR signaling on the host surface is required for morphogenesis of the specialized appressorium infection cell [13, 14]; active TOR signaling following penetration into rice epidermal cells drives early biotrophic growth and the elaboration of IH [12]. Here, we identified a new TOR signaling component, encoded by *IMP1*, involved in autophagy induction. Basal autophagy was not abolished by the loss of *IMP1*, and Δ*imp1* strains could form mostly functional appressoria on host rice leaves, allowing us to investigate the role of *IMP1 in planta*. We subsequently discovered that the novel TOR-Imp1-autophagy signaling axis integrates hyphal growth with biotrophic interface integrity in rice cells. This provides fresh insight on the fundamental molecular processes underlying plant-fungal interactions.

### Imp1 roles in vacuole function

We discovered *IMP1* in a forward genetic screen for mutations conferring rapamycin resistance. The original suppressor strain, AT2, was the only one of six rapamycin resistant mutants that sporulated. The other rapamycin-resistant suppressor strains were not examined but might be expected to have some direct role in TOR signaling, potentially via nutrient sensing or the control of central metabolism because sporulation is an energy intensive process. We can assume, however, that these other mutants do not likely result from lesions in the *FPR1* gene, which would also confer rapamycin resistance, because Δ*fpr1* sporulates like WT [13]. *IMP1* encodes a vacuolar protein required for membrane trafficking, V-ATPase assembly, organelle acidification and autophagy induction. Autophagy induction (but not basal autophagy) was blocked in Δ*imp1* strains when TOR was inactivated by rapamycin or starvation conditions, indicating, along with studies of TOR kinase function and other TOR readouts, that *IMP1* functions downstream of TOR in the autophagy-signaling branch. The loss of *IMP1* also misregulated V-ATPase assembly both *in planta*, and during axenic growth in response to glucose. Other processes associated with vacuole function were perturbed in Δ*imp1*, including pH and metal homeostasis, temperature and oxygen sensitivity. Therefore, *IMP1* functions to relay nutrient signals from TOR to the vacuole.

Although impaired for V-ATPase assembly and organelle acidification, Δ*imp1* displayed only a partial *vma*^*-*^ phenotype. This is in line with a recent study in yeast where the Sch9 kinase, localized at the vacuolar membrane in exponentially growing yeast cells, was also shown to connect TOR signaling to V-ATPase assembly and activity, thereby regulating vacuolar acidity and cellular longevity [45]. Like Δ*imp1*, loss of *SCH9* conferred a partial *vma*^*-*^ phenotype on yeast cells. Also, rapamycin-induced V-ATPase assembly in yeast required *SCH9*, and *sch9Δ* mutants were altered in their response to rapamycin in growth media [45]. Thus, yeast *sch9Δ* and *M*. *oryzae* Δ*imp1* mutant phenotypes are similar enough to bolster support for our argument that *IMP1* connects TOR signaling to vacuole acidification and function. Our results thus contribute toward answering the important but largely unresolved question of how vacuole acidification is controlled [45].

### Imp1 roles in maintaining interface membrane integrity

Where does Imp1 act in the autophagy pathway, and how might this role relate to biotrophic interface integrity maintenance? We propose that Imp1 facilitates phagophore expansion and autophagosome formation during autophagy induction by sourcing membranes from endosomes (**Fig 11B**). The absence of this activity in Δ*imp1* affects membrane homeostasis and triggers biotrophic membrane integrity failure. Our reasoning is thus: Nonselective macroautophagy (autophagy) involves dynamic rearrangements of subcellular membranes [49]. Following autophagy induction and phagophore nucleation, the phagophore membrane expands to sequester cargo and generate autophagosomes [49, 50]. Membrane vesicles from numerous endomembrane compartments including the plasma membrane and recycling endosomes contribute lipids for the expansion of phagophores [50, 51, 68, 69]. Compared to the proteins involved in autophagy, little is known about the source of membranes involved in phagophore initiation and expansion [68], or the mechanisms involved in the transport of membrane vesicles from endomembrane compartments to the phagophore [50]. The plasma membrane, in addition to providing membranes for phagophore expansion, is also delivered by endocytic machinery to contribute to the pre-autophagosome structure/ phagophore assembly site (PAS), the immediate precursor of the phagophore [51]. Once formed from phagophores, autophagosomes fuse directly to vacuoles, releasing the inner autophagosome vesicle and cargo for degradation into the vacuole lumen, which becomes an autophagic body [49, 50]. Four lines of evidence, when considered together, support our hypothesis that Imp1 is involved in the delivery of the plasma membrane, via endocytosis, for phagophore expansion during autophagy induction. First, blocking phagophore initiation with 3-MA in WT phenocopied Δ*imp1*, resulting in the loss of membrane integrity, while inhibiting V-ATPase assembly, organelle acidification and vacuole function, which were also Imp1-dependent processes, affected IH growth but did not affect membrane integrity (**Fig 11B**). This indicates that in order to maintain biotrophic membrane integrity during fungal growth in rice cells, Imp1 must act between phagophore initiation on one hand, and V-ATPase activity, vacuole function and late-stage autophagy on the other. Second, Imp1^GFP^ is localized to both vacuoles and IH membranes, and components of early autophagosome precursors can also be found at the plasma membrane [51]. Imp1 is lost from IH following 3-MA treatment. Third, Imp1 is not required for endocytosis. Fourth, the overlap between FM4-64 labeled vesicles and MDC-labeled compartments was almost abolished in Δ*imp1* hyphae compared to WT. Because MDC will stain vacuoles that have fused with autophagosomes, this could indicate that endosomes but not autophagosomes are defective in fusing to Δ*imp1* vacuoles. However, in Δ*imp1*, not only was there less overlap between endosomes and autophagosomes, but the number of MDC-stained autophagic structures was also reduced compared to WT. How can we account for a mechanism that would both determine the degree of endosome and autophagosome overlap, and affect the number of autophagic vacuoles in hyphae? We hypothesize that endosomes might be contributing membranes to autophagosome formation in an Imp1-dependent manner. This would not likely occur by direct autophagosome-endosome fusion, because amphisomes are not formed by yeast [49], but rather by the trafficking of plasma membrane vesicles to the expanding phagophore (**Fig 11B**). Reduced vesicle membrane trafficking in Δ*imp1* would result in both reduced overlap between labeled compartments, and an overall reduction in the number of autophagic structures.

In support of our claim that Imp1 mediates membrane vesicle trafficking to facilitate autophagosome production, we note that clathrin-mediated endocytosis regulates autophagosome formation in mammalian cells [70]. Plasma membrane delivered in this manner are important for the massive increase in autophagosome biosynthesis required under inductive but not basal levels of autophagy [70]. Consequently, inhibition of endocytosis decreased autophagosome formation in this system by 30% [70]. If Imp1 connects the plasma membrane with autophagosome formation in *M*. *oryzae*, this might similarly occur during increased autophagosome biosynthesis and could account for why Imp1 is required for inductive but not basal autophagy. We also note that different membrane sources contribute to autophagosome formation in response to different stimuli, for example mammalian mitochondrial membranes only contribute to autophagosome formation following starvation [51]. This might explain why Imp1 only localizes to plasma membranes in IH during *in planta* biotrophic growth, or in vegetative hyphae following rapamycin treatment.

When all evidence is considered together, it is conceivable that, during plant infection, as WT IH fill the first infected cell, nutrient becomes exhausted and TOR is inactivated, inducing autophagy and increasing demand for autophagosome production over basal levels. Plasma membranes and/ or recycling endosomes might then be recruited in an Imp1-dependent manner to contribute more membranes for phagophore expansion and autophagosome formation. Furthermore, in yeast, recycling endosomes can traffic internalized integral membrane proteins from the plasma membrane into different cellular pathways, including the vacuole for degradation, or direct their return to the plasma membrane [71]. If Imp1 directs similar membrane traffic in *M*. *oryzae* IH in order to balance plasma membrane recycling with the delivery of membranes to phagophores when autophagosome demand is high, then impaired delivery of membrane vesicles in Δ*imp1* would mean multiple cellular demands for membranes are not met. This would affect membrane homeostasis and might increasingly impact BIC and EIHM integrity as biotrophy progresses, resulting in the rapid and complete loss of BICs, and the erosion of the EIHM over time. In addition, plasma membrane recycling might bypass the vacuole [71], indicating why ConA and BafA1 treatments do not erode the interfacial membrane. Taken together, Imp1 might mediate endomembrane trafficking and membrane homeostasis to balance interfacial longevity with nutrient availability under the challenging growth conditions of the living rice cell (**Fig 11B**).

Although other processes could also be directly or indirectly affected by the loss of *IMP1* and account for the observed phenotypes, and while we acknowledge 1) that compared to yeast we are limited in our tools for analyzing membrane trafficking events and autophagic processes in filamentous fungi, especially during growth *in planta*, and 2) that the membrane origins of autophagy are still poorly resolved in any system [50, 70], our hypothesis regarding the role of Imp1 in membrane trafficking and interface longevity provides a framework for interpreting the role of TOR signaling and autophagy in maintaining membrane integrity in intracellular symbionts.

### TOR signaling and Imp1 activity

*M*. *oryzae* TOR signaling is dynamic during host cell growth, being active during the very early stages of biotrophy but inactive during later growth in the first infected cell in order to maintain membrane integrity and promote cell-to-cell movement. How and whether TOR activity status controls Imp1 function in response to changing stimuli in order to propagate the autophagy induction signal and mediate autophagosome production when necessary is not known. However, we could find no evidence from immunoblot analyses of changes in Imp1 processing following growth in TOR activating (glucose) versus inactivating (rapamycin) conditions, or as biotrophy progressed. Furthermore, Imp1 posttranslational modifications such as phosphorylation were not evident across conditions. Thus, Imp1 might not be a direct or indirect target of TOR signaling. Also, it is not yet clear how Imp1 functions to mediate the autophagy response. Clues to answering these questions about Imp1 regulation and function might come from the knowledge that AM treatment, which affects Ca^2+^ levels, remediates Δ*imp1*, suggesting Imp1 might play a role in modulating cytosolic Ca^2+^ in order to facilitate vesicle trafficking and membrane fusion in response to TOR-dependent autophagy induction. If so, Imp1 might be a structural—rather than regulatory—component of the TOR-autophagy signaling pathway that is recruited to facilitate early autophagy but is not rendered active by TOR. Thus, our current favored hypothesis is that Imp1 is a structural component of the TOR-autophagy signaling branch and is not subjected to direct TOR regulation. To test this hypothesis, future work might explore the connection between Imp1 and Ca^2+^ metabolism, attempt to confirm whether and how TOR (directly or indirectly) regulates Imp1 function, establish if the predicted N- and C-terminal domains of Imp1 are involved in responses to TOR, and ascertain whether Imp1 senses nutrient cues independently of TOR.

Stimulating autophagy with AM promoted cell-to-cell movement of WT and Δ*imp1* by increasing the number of individual hyphae that moved to adjacent cells, indicating autophagy promotes sustained biotrophic colonization. Recently, the MAP kinase Pmk1 was shown to control IH movement into adjacent cells through plasmodesmata [72]. Because TOR can engage the cAMP/PKA/Pmk1 signaling pathway upstream of Pmk1 during appressorium formation [13], it will be interesting to determine the nature of the relationship between TOR, autophagy and Pmk1 during growth in rice cells.

### Insights on the biotrophy to necrotrophy transition

*M*. *oryzae* is a hemibiotroph characterized by the transition into necrotrophy after 3–5 days of biotrophic growth in living rice cells [6]. Initially, it seemed plausible that the poor biotrophic growth of Δ*imp1* resulted in an early transition to necrotrophy and the subsequent loss of biotrophic membrane integrity. If so, the loss of virulence would be the proximal cause of interface erosion. However, three lines of evidence suggested this is not the case and instead supported the fact that impaired biotrophic growth does not implicitly lead to early necrotrophy and the loss of biotrophic interface integrity. Firstly, AM treatment of Δ*imp1* reconstituted the EIHM and BIC, along with stimulating biotrophic growth. This would not be possible if Δ*imp1* had transitioned into necrotrophy, which would likely be irreversible. Secondly, WT treatment with ConA and BafA1 inhibited biotrophic growth but did not result in interface loss. Thirdly, a previous, unrelated study showed how biotrophic growth of the Δ*nmo2* mutant was attenuated in the first infected cell, but Bas4^GFP^ outlined Δ*nmo2* IH and the BIC was visible, albeit fragmented, and still secreting Pwl2^mCherry:NLS^ into rice cells [11]. We conclude that the loss of biotrophic interfacial membrane integrity is not necessarily a function of the loss of biotrophic growth and, moreover, attenuated biotrophic growth does not lead to early entry into necrotrophy. Rather, biotrophic membrane integrity is dependent upon TOR-autophagy signaling status, and biotrophic growth is attenuated in a reversible manner when the biotrophic interface erodes. Because biotrophic interface erosion must eventually occur during necrotrophy, we propose that autophagy-related processes regulated by TOR are likely involved in the biotrophy-necrotrophy lifestyle transition.

### The control of biotrophic membrane integrity and effector secretion

Fungal phytopathogens suppress host innate immunity by deploying cytoplasmic and apoplastic effectors, resulting in colonization and devastating crop losses. Preventing effector secretion into host cells would impair colonization, yet molecular pathways controlling effector secretion are unknown. Here, we showed how the TOR-Imp1-autophagy signaling axis ensures correct effector deployment by coordinating biotrophic interface maintenance with fungal growth in rice cells. Elaborating this relationship improves our understanding of the requirements for effector secretion and, because Pwl2^mCherry:NLS^ is not observed in Δ*imp1* IH or the nuclei of Δ*imp1*-infected rice cells after initial infection, our results hint at how effector secretion might be regulated *in planta*. Such knowledge might be leveraged in the future towards uncovering novel sources of plant disease resistance.

### Conclusions and significance

Our work uncovered two new insights with broad applicability to other systems: we discovered a new TOR signaling component, Imp1, and we revealed how fungal TOR signaling, via autophagy, dictates the longevity of the biotrophic interface between fungus and plant. In addition to vacuole functions, we conclude that Imp1 has a structural role in TOR-dependent autophagy induction by facilitating phagophore expansion.

Molecular mechanisms that regulate or maintain biotrophic interfaces and plant-fungal interfacial zones as fungi grow in plant cells are unknown and in general, genes known to be required for biotrophy are sparse. By providing the first evidence to suggest that TOR control of fungal autophagy is required for maintaining BIC and EIHM integrity during biotrophy, we reveal new molecular targets in the quest to manipulate plant-microbe interactions and improve crop productivity. We also highlight how the metabolic status of the fungal cell drives and dominates this interkingdom interface. Our results might also guide studies to understand the molecular regulators of the biotrophic to necrotrophic transition, a process likely widespread even amongst those fungal pathogens that are predominantly necrotrophic.

During a eukaryote-prokaryote interaction between *Dictyostelium* and *Mycobacterium*, autophagy-derived membranes maintain host cell plasma membrane integrity and promote cell-to-cell transmission of the pathogen [73]. This suggests, when considered along with the results presented here, that autophagy and its regulation are fundamental principles of intracellular host-symbiont interactions across kingdoms.

## Materials and methods

### Strain maintenance

The wild type (WT) rice-infecting strain of *Magnaporthe oryzae* used in this study was Guy11 [3]. Mutant strains used in this study were derived from Guy11 and are listed in S3 Table. Strains were grown on complete media (CM) for routine maintenance, conidia harvesting and growth testing. CM contains 1% (W/V) glucose, 0.2% (W/V) peptone, 0.1% (W/V) yeast extract, 0.1% (W/V) casamino acids and pH adjusted to 7.5 with NaOH. Strains were also grown on Cove's glucose minimal medium (GMM) with 1% (w/v) glucose as the sole carbon source and 10 mM nitrate as the sole nitrogen source, unless other wise specified. Plates were incubated at 26°C under 12 hr light / dark cycles for 10–15 days. 85 mm petri dishes were used throughout. Plate images were taken with a Sony Cyber-shot digital camera, 14.1 megapixels.

### Random mutagenesis using *Agrobacterium tumefaciens-*mediated transformation (ATMT)

ATMT was conducted as described previously [9, 18]. Briefly, *M*. *oryzae* mycelia from the edge of 5-day old colonies were excised, blended and grown in liquid CM for two days. *Agrobacterium* strain *AGL1* carrying the pKHt plasmid [74] containing a hygromycin resistance marker (*hph*) flanked by T-DNA for random insertion was cultured in AIM liquid media for one day. *M*. *oryzae* mycelia were then co-incubated with the *Agrobacterium AGL1* liquid AIM culture supplemented with 200 μM acetosyringone (AS) at 100 rpm for 1 hour at 28°C. This combined culture was spread onto cellulose nitrate (CN) membranes placed on 50 mm petri dishes containing solid AIM with 200 μM AS. The plates were incubated for 48 hours at 22°C in dark after which time, CN membranes were buried beneath a metabolic selection media consisting of minimal media with 1% glucose as the sole carbon resource and 10 mM NH_4_^+^ as the sole nitrogen resource [18] and containing 55 μM rapamycin as well as 100 μg/mL carbenicillin, 400 μg/mL cefotaxime, 100 μg/mL chloramphenicol, 100 μg/mL hygromycin, 50 μg/mL kanamycin, and 60 μg/mL streptomycin, then incubated at 28°C for 5–10 days or until colonies emerged. Emerging rapamycin resistant colonies were inoculated onto two rounds of purification media containing the same metabolic selection media and antibiotics to eliminate *Agrobacterium*.

### Identification of the T-DNA insertion site

As previously noted [9, 18], dual ATMT selection yields only a small number of mutant strains. Here, we recovered 6 stable mutant strains resistant to both hygromycin and rapamycin. From an initial assessment, only the rapamycin resistant ATMT transformant designated AT2 produced spores, suggesting this mutant might be amenable to downstream analyses. DNA extracted from AT2 was used as a template for Thermal Asymmetric Interlaced Polymerase Chain Reaction (TAIL-PCR) to identify the T-DNA insertion site [9]. Three rounds of PCR using T-DNA border specific primers and random primers (S4 Table) were employed to attempt to amplify the left right T-DNA borders and adjacent genomic DNA. Subsequent rounds of amplifications used *hph* specific primers walking outwards from previous T-DNA primers to maximize specificity. The amplified sequences were then separated on a 1.2% agrose gel and purified by a Wizard SV Gel and PCR Clean-Up System (Promega, A9281). The purified PCR fragments were ligated into the pGEM-T vector following the protocol of pGEM-T Easy Vector Systems (Promega, A1360). Afterward, 10 μL of the ligation products were transformed into the competent *E*. *coli* (*JM-109*; Promega, L2001) for plasmid amplification. Plasmids were extracted from a 3 mL culture of the transformed *JM-109* strain by a Wizard Plus SV Minipreps DNA Purification System (Promega, A1330) and sequenced by Eurofins Genomics.

### Targeted gene replacement

WT protoplasts were generated and transformed using previously described methods [17]. The Δ*imp1* single mutant was generated by replacing the entire coding region of *IMP1* (MGG_08120) with *ILV1* conferring sulphonyl urea resistance [17]. Briefly, 1 Kb of the left flank (LF) and right flank (RF) of the *IMP1* coding region were amplified using the primers *IMP1-1* (LF5′) & *IMP1-2* (LF3′) and *IMP1-3* (RF5′) & *IMP1-4* (RF3′), respectively (S4 Table). The 5′ region of the *ILV1* gene was amplified using the primer pair M13F:SU and SuSplit, and the 3′ region of the *ILV1* gene was amplified using the primer pair M13R:UR and UrSplit. The *IMP1* left flank amplicon, and the 5′ region of the *ILV1* gene, were fused by amplifying with NesF and SuSplit. The *IMP1* right flank amplicon, and the 3′ region of the *ILV1* gene, were fused by amplifying with NesR and UrSplit. The two resulting fragments, which overlap in the *ILV1* gene by approximately 300 bps, were transformed into protoplasts and transformants were initially selected using BDCM-TOP media containing 50 μg/ mL (final concentration) sulphonyl urea. Targeted gene deletion was confirmed by amplification of the entire coding region of *IMP1* with primer *IMP1-1* (LF5′) and *IMP1-4* (RF3′). The primers used are shown in S4 Table.

### Generation of *IMP1*^*GFP*^ fusion constructs and Δ*imp1* complementation

The full-length *IMP1* gene, along with its 1.5-kb native promoter region, was cloned into pDL2 –which carries the green fluorescent protein (GFP) -encoding gene and the *hph* cassette conferring hygromycin B resistance—by yeast gap repair as previously described [75]. The resulting *IMP*^*GFP*^ fusion construct was confirmed by sequencing analysis and, along with the *OEIMP1*^*GFP*^ fusion construct that was similarly made but carried the RP27 constitutive promoter instead of the native promoter, was transformed into protoplasts of the Δ*imp1* mutant strain. Transformants resistant to both suphonyl urea and hygromycin were screened by PCR, GFP fluorescence and restoration of rapamycin sensitivity in order to confirm complementation by Imp1^GFP^. Primers used are listed in S4 Table.

### Gene transcript analysis

For gene transcription analysis by quantitative real-time PCR (qPCR) during vegetative growth, young mycelia at the colony edge were separated from 5 day old colonies of Δ*imp1*, Δ*fpr1* and WT and then cultured in liquid CM for 16 hours followed by 3 times wash with ddiH_2_O before switching to liquid CM with or without 1 μM rapamycin for 16 hours, or to liquid MM with nitrate as the sole nitrogen source for 8 hours. Mycelia were then frozen, lyophilized for 72 hours and ground in liquid nitrogen. For *in planta* gene transcript analysis, rice leaf sheaths were dissected for RNA extraction at the indicated time points. RNA was extracted from each sample using the RNeasy mini kit from Qiagen. After treatment with DNase I (Invitrogen), RNA was converted to cDNA using qScript (Quantas). qPCR was performed on an Eppendorf Mastercycler Realplex using the recommended reagents with primers listed in S4 Table. qPCR data was analyzed using the Realplex software package. Thermocycler conditions were: 5 min at 95°C, followed by 40 cycles of 95°C for 30 sec, 63°C for 30 sec and 72°C for 30 sec. Fold changes were calculated using the ΔΔ^Ct^ method [14].

### Pathogenicity assays

For appressorial formation analysis on artificial surfaces, spores were harvested from 10 day old colonies of WT, the Δ*imp1* mutant and the Δ*imp1 IMP1*^*GFP*^ complementation strain following growth on CM media. The harvested spores were suspended in ddiH_2_O to 1×10^4^ spores per mL. 200 μL of each spore suspension was inoculated in triplicate onto inducible hydrophobic plastic coverslip or noninducible hydrophilic glass slides and placed in a humid chamber in the dark at 22°C for 24 hours. Indicated treatments were directly added to the spore suspensions on the surfaces.

For whole plant inoculations, spores were harvested from 10 day old colonies of each indicated strain grown on CM media. The harvested spores were suspended in 0.02% gelatin at a rate of 1×10^5^ spores per mL. 10 mL spore suspension was then evenly sprayed onto 3-week old CO-39 rice seedlings. The inoculated rice seedlings were incubated in a humidity chamber for four days. The infected leaves were detached from the rice seedlings and dehydrated for five days before being scanned using an Epson Perfection V550 scanner at a resolution of 600 dpi.

For the rice leaf sheath assays, spores of the indicated strains were harvested from 10 day old colonies grown on CM and then suspended in 0.2% gelatin solution at the rate of 1 × 10^5^ spores per mL. Spore suspensions were inoculated into the hollow of the leaf sheaths detached from 4–5-week-old rice seedlings and incubated in the dark for 24 hours at 25°C. Rates of appressorium formation were determined by counting how many of 50 germinating spores per rice cuticle formed appressoria by the indicated time points, replicated in triplicate for each treatment. Rates of penetration by appressoria at the indicated time points were determined from observing 50 appressoria per rice cuticle, replicated in triplicate for each treatment. Rates of IH conducting cell-to-cell movement was determined from observing how many of 50 IH in primary infected cells had emerged into adjacent rice cells by the indicated time points, replicated in triplicate for each treatment.

### Confocal microscopy

For Imp1^GFP^ localization in vegetative mycelia, Δ*imp1 IMP1*^*GFP*^ mycelia from 5-day old colonies grown on CM media were cultured in CM liquid media for 48 hours followed by growth in GMM liquid media for 3 hours (to maintain glucose-rich conditions) or for 16 hours (to generate glucose-depleted conditions). For Imp1^GFP^ localization during appressorium formation, spores harvested from 10 day old colonies of Δ*imp1 IMP1*^*GFP*^ were suspended in water at a rate 1 × 10^4^ spores per ml. 200 μL spore suspension were inoculated onto a hydrophobic plastic coverslip and incubated in the dark for 24 hours at 22°C. For Imp1^GFP^, Vma2^GFP^, Pwl2^mCherry:NLS^ and Bas4^GFP^ localization *in planta*, spores harvested from 10 day old colonies of each strain were suspended in 0.02% gelatin at 1×10^5^ spores per mL. Spore suspensions were injected into the hollow of healthy rice leaf sheaths detached from 4-week CO-39 seedlings and incubated in a humidity chamber in the dark for 44 hours. Epidermal layers of inoculated leaf sheaths were separated with a double-edge razor blade. Images were taken using a Nikon A1 laser scanning confocal mounted on a Nikon 90i compound microscope (software version: NIS Elements 4.13 Build914) at the University of Nebraska-Lincoln Microscopy Center. Excitation/emission was 488 nm/505–550 nm for GFP and 543 nm/560–615 nm for mCherry. The *VMA2*^*GFP*^ construct was assembled as described above for *IMP1*^*GFP*^, using the primers listed in S4 Table, and transformed into WT and Δ*imp1* strains.

For acidic compartment observations, vegetative mycelia of the indicated strains were incubated in glucose minimal media (GMM), water or GMM supplemented with 1 μM rapamycin or 1 μM amiodarone hydrochloride (AM) for 3 hours and then incubated with1 μg/mL quinacrine for 10 mins on a hydrophobic plastic coverslip. After mounting on a glass coverslip, a drop of water was added to one side of the coverslip and absorbed from the opposite side, three times, to rinse off the unbound quinacrine. For endocytosis tracking, vegetative mycelia of the indicated strains were harvested following growth in CM for 48 hours and transferred to liquid GMM for 16 hours to generate glucose-depleted conditions. Mycelia were then incubated with one μg/mL FM4-64 (Sigma-Aldrich, USA) for 1 and 5 hours to observe the internalization of FM4-64. For autophagosome and autophagic vacuole staining, mycelia grown in GMM for 16h were stained with 40 μM Monodansylcadaverine (MDC) for 5 h. General differential interference contrast (DIC) microscopy was performed with a Carl Zeiss Axioskop 50 microscope. Images were acquired using a Zeiss AxioCam HRc camera and analyzed with Axiovision 3.1 software. Confocal imaging was performed with a Nikon A1 laser scanning confocal mounted on a Nikon 90i compound microscope (software version: NIS Elements 4.13). Excitation/emission was 488 nm/505–550 nm for MDC and quinacrine, and 543 nm/560–615 nm for FM4-64. For dual color GFP and mCherry, and MDC and FM4-64, each channel was acquired sequentially to avoid emission crosstalk. Images were acquired and processed using NIS-Elements or ImageJ.

### *In planta* treatments

Leaf sheaths were inoculated as described above and incubated in humid chamber for indicated hours at 25°C in the dark. Inner epidermal cell layers were trimmed by a double-edged razor blade and subjected to laser scanning confocal microscopy. Treatments were applied at the indicated time points after the primary spore suspension was gently removed by tapping one end of the sheath with sterilized paper towels. The treatments used for this study were 10 μM rapamycin (Rap; LC Laboratories, USA), 2 μM amiodarone hydrochloride (AM; Fisher Scientific, USA), 5 mM 3-methyladenine (3-MA; Fisher Scientific, USA), 10 μM concanamycin A (ConA; Fisher Scientific, USA), 1 μM bafilomycin A1 (BafA1; Fisher Scientific, USA). ANOVA analysis and *student’s t-test* were performed in software Infostat version 2014e.

For 3,3′-Diaminobenzidine (DAB) staining, epidermal cell layers of the infected leaf sheathes were trimmed by sterilized double blades, soaked in 1 mg/ml DAB for 2 hours, and washed in ethanol: acetic acid solution (94:1 v/v) for 2 hours. The samples were examined by an EVOS digital microscope.

### Western blot

To detect Imp1^GFP^ in *M*. *oryzae* mycelia, the Δ*imp1 IMP1*^*GFP*^ strain was grown in shaking CM media for 48 hours and then washed with distilled water three times. Washed mycelia were transferred to the appropriate media and incubated at 26°C for 3 hours. To detect Vma2^GFP^ in *M*. *oryzae* mycelia, the WT *VMA2*^*GFP*^ and Δ*imp1 VMA2*^*GFP*^ strains were grown in CM as above and the mycelia was transferred after washing to GMM for 3 hours. To detect S6K1/Sch9 phospho-status, WT, Δ*imp1* and Δ*fpr1* were grown in CM as above and the mycelia transferred to fresh CM with and without 1 μM rapamycin (Rap) for 8h. Mycelia harvested from the second growth regime were washed with distilled water three times and finely ground in liquid nitrogen. For detecting Imp1^GFP^ accumulation during *M*. *oryzae* growth in leaf cells, 48 leaf sheathes were detached from 4-week-old rice seedlings and cut to approximate 70 mm long. 24 leaf sheaths were inoculated with spores of Δ*imp1 IMP1*^GFP^ and 24 leaf sheaths were incubated with deionized distilled water. For Vma2^*GFP*^ *in planta* detection, 8 leaf sheathes were inoculated with spore suspensions of WT *VMA2*^*GFP*^ *or* Δ*imp1 VMA2*^*GFP*^ strains. All inoculated leaf sheathes were incubated in the dark humid chambers. 8 leaf sheaths inoculated with Δ*imp1 IMP1*^GFP^ or the water control were collected at 28 hpi, 36 hpi, and 44 hpi. WT *VMA2*^*GFP*^ *or* Δ*imp1 VMA2*^*GFP*^ infected leaf sheathes were collected at 44 hpi. Green epidermal cell layers of the leaf sheathes were trimmed off and then immediately ground in liquid nitrogen.

For Imp1^GFP^ and Vma2^GFP^, 200 mg of ground material were immediately suspended in 400 μL of 2X sample buffer (100 mM Tris-HCl, pH 6.8, 4% (w/v) SDS, 0.2% (w/v) bromophenol blue, 20% (v/v) glycerol, 200 mM DTT, 5% (v/v) β-mercaptoethanol) and incubated at 95°C for five minutes after which the samples were centrifuged at 4,700 rpm for five min to extract the proteins. The protein samples were boiled at 95°C again and 30 μL from each protein sample were loaded to SDS-PAGE and run for 40 minutes at 120 V for fractionation. The fractionated protein samples were then transferred to Immun-Blot PVDF membrane (Bio-Rad, USA) by sandwiching SDS-PAGE and Immun-Blot PVDF membrane and subject to 30 V overnight. GFP and α-tubulin was immunoblotted with monoclonal anti-α- GFP (1:1000 dilution; Sigma-Aldrich, USA) and anti-α-tubulin (1:1000 dilution; Santa Cruz Biotechnology, USA) antibodies, respectively. According to the validated antibody database at Labome, the α-tubulin antibodies from Santa Cruz Biotechnology have reactivity against α-tubulin from fungi and human but not plant. Secondary antibodies were used at 1:10,000 dilutions. The Clarity Western ECL substrate (Bio-Rad, USA) was used to develop the blots. Images were taken with the ChemiDoc XRS+ (Bio-Rad, USA), using the Chemi Hi Resolution application. The bands were analyzed using Image Lab (software version 5.2.1, Bio-Rad). Relative GFP signal intensity was obtained by normalizing against α-tubulin and correcting for the background determined from a WT control strain.

For phospho-status analysis, equal amounts of mycelia powder were used for total protein extraction in freshly prepared cell lysis buffer (60 mM Tris-HCl, pH 6.8, 2% SDS, 10% (w/v) glycerol, 5% β-mercaptoethanol) supplemented with protease inhibitors (200 mM AEBSF, 20 mM Bestatin, 5 mM E-64, 10 mM Leupeptin, 10 mM Pepstatin A, 500 mM 1,10-Phenanthroline, 5 mM EDTA, 1 mM PMSF) and phosphatase inhibitors (20 mM NaF, 0.2 M okadaic acid, 20 mM b-glycerophosphate, 5 mM Na3VO4), followed by denaturation at 95°C for 3 min. The cell lysates were cleared by centrifugation at 16,000 g for 15 min at 4°C, and equal volumes of total proteins in lysates were resolved by 12% SDS-PAGE and then transferred to a PVDF membrane. Phosphorylation status of S6K1/Sch9 was monitored using anti-p-p70 S6 kinase α mouse monoclonal antibody (Santa Cruz Biotechnology) and normalized to α-tubulin. Western blots were visualized using horseradish peroxidase-conjugated secondary antibodies (goat anti-Mouse IgG) (Sigma) for p-p70 S6 kinase α and goat anti-rat IgG (Santa Cruz Biotechnology) for tubulin α. Low temperatures (4°C), protease inhibitors and phosphatase inhibitors were applied throughout the western blot analysis, including protein transfer and antibody binding. The blots were imaged using Clarity Western ECL chemiluminescent system (Bio-Rad) and quantitated by densitometry using ImageJ analysis software (imagej.net/Welcome).

### V-ATPase activity assays

Vesicle membranes were extracted from the protoplasts following the protocol of Chanda and colleagues [76]. Protoplasts of *M*. *oryzae* were generated and suspended in STC buffer. 500 μL of the protoplast suspension were then mixed with 1.5 mL protoplast lysis solution (0.6 M sorbitol, 10 mM Tris-Cl, 0.025% Triton-X100 pH 7.5) for 15 min. Following lysis, 1000 μL lysis mixture was overlayed onto 1 mL sucrose cushion (3 M sucrose, 1.2 M sorbitol, 10 mM Tris-HCl pH = 7.5) and centrifuged at 3000×g at room temperature for 45 minutes. 100 μL of liquid containing vacuolar vesicles was harvested from the interface, and 50 μL was added to a cuvette containing 1 mL of the ATP hydrolysis assay solution [55] containing 25 mM HEPES pH 7.0, 25 mM KCl, 5 mM MgCl_2_, 2 mM phosphoenolpyruvate (Rabbit Muscle, Sigma, USA), 2 mM ATP and 0.5 mM NADH. pH was adjusted to 7.0 with KOH before 30 units of L-lactate dehydrogenase (Rabbit Muscle, Sigma, USA) and 30 units of pyruvate kinase (Rabbit Muscle, Sigma, USA) were added. Absorbance change at 340 nm was immediately observed by a spectrophotometer. V-ATPase-independent ATPase activity was determined by the decrease in absorbance due to the addition of 100–300 nM of the specific V-ATPase inhibitor concanamycin A (ConA; Santa Cruz Biotechnology). Absorbance readings were linear up to an A340 value of 3.0. The molar extinction coefficient for NADH (e) is 6.22 mM^-1^cm^-1^ and depletion of NADH was directly correlated to ATP hydrolysis. Specific activity corresponds to micromoles ATP hydrolyzed per minute per milligram protein.

For the proton pumping assay, approx. 200 μg of separated vacuolar vesicle proteins and 20 μM acridine orange (AO) were added to the assay solution. The pH was adjusted to 7.0 with KOH before 30 units of L-lactate dehydrogenase (Rabbit Muscle, Sigma, USA) and 30 units of pyruvate kinase (Rabbit Muscle, Sigma, USA) were added [55]. Absorbance change at 495 nm was immediately observed by spectrophotometry. Absorbance quenching of the ΔpH probe acridine orange at λ495 nm was directly correlated with proton uptake by vesicles in the assay media.

## Supporting information

S1 Fig Six rapamycin resistant mutant strains were generated by *Agrobacterium tumefaciens*-mediated transformation (ATMT). (A) Strains are shown after growing for 10 days on minimal media (MM) with 1% (w/v) glucose as the sole carbon source and 10 mM NH_4_^+^ as the sole nitrogen resource. Rap is 10 μM rapamycin. NT is no treatment. (B) Of the six Rap resistant mutant strains generated by ATMT, only AT2 sporulated at similar rates to WT. Bars are the mean number of spores harvested from three 10-day-old plates. Error bars are s.d. Bars with different letters indicate significant difference (α ≤ 0.05, Least significant difference (LSD)).
(TIF)

S2 Fig *IMP1* is required for appressorium morphogenesis on artificial hydrophobic surfaces and cell-to-cell biotrophic growth in rice cells. (A) The clean knockout strain of *IMP1*, Δ*imp1*, sporulated at marginally reduced rates compared to WT and the Δ*imp1 IMP1*^*GFP*^ complementation strain. Bars are the mean number of spores harvested from three 10-day-old plates. Error bars are s.d. Bars with different letters indicate significant difference (α ≤ 0.05, LSD). (B) Following spore germination on artificial hydrophobic surfaces, Imp1^GFP^ had, by 24 hpi, localized to compartments in the appressorium. (C) Appressorium formation on artificial hydrophobic surfaces in Δ*imp1* compared to WT. Images are representative of the observed phenotypes. % is the proportion of germinating spores displaying the indicated morphology by 24 hpi. (D) Biotrophic growth was impaired in Δ*imp1*. Stars indicate emerging invasive hyphae (IH) in adjacent cells. Arrows indicate appressoria on the leaf sheath surface. Scale bars = 10 μm.
(TIF)

S3 Fig Imp1^GFP^ localizates to the vacuole in vegetative mycelia. Vegetative mycelia were grown in the indicated treatments for 3 h. GMM = glucose minimal media. Rap = 1 μM rapamycin. AM = 1 μM amiodarone hydrochloride, a TOR-independent autophagy stimulator. Scale bar = 5 μm.
(TIF)

S4 Fig *IMP1* is required for maintaining the V-ATPase-dependent proton gradient. (A) V-ATPase-dependent ATP hydrolysis activity was not different in protoplast vesicles of Δ*imp1* and WT liberated from vegetative mycelia grown in glucose-rich complete media (CM). V-ATPase activity was determined as the reduction in the amount of ATP hydrolysed following treatment with 200 nM of the V-ATPase inhibitor concanamycin A (ConA) compared to the amount of ATP hydrolyzed by untreated samples (NT). (B) V-ATPase-dependent proton pumping activity, determined from the reduction of absorbance quenching of the ΔpH probe acridine orange, was not detectably different during early time points in protoplast vesicles of Δ*imp1* and WT liberated from vegetative mycelia grown in glucose-rich complete media (CM). However, differences in the rates of absorbance quenching emerged at later time points suggesting *IMP1* is required for maintaining the pH gradient.
(TIF)

S5 Fig *IMP1* is partially required for canonical vacuole functions. (A,B, D) Strains were grown for 10 days on defined glucose minimal media with the indicated treatments. NT = no treatment. (C) The strains were grown in 100 mm petri dishes filled half-full with 25 ml complete media (CM), per our normal protocol, or filled to the top with CM, leaving only a 2–5 mm space between the media surface and the lid, and sealed with parafilm to generate hypoxia stress. Plates were incubated for 12 days. (E) Spores were harvested from plates of the indicated pH at 12 days. Bars are the average of three independent replicates, error bars are s.d.
(TIF)

S6 Fig Concanamycin A treatment does not render *M*. *oryzae* rapamycin resistant. WT and Δ*imp1* were grown in CM supplemented with 50 nM ConA, 10 μM rapamycin or both for 12 days. NT = no treatment.
(TIF)

S7 Fig Effector genes are expressed in Δ*imp1* during growth *in planta*. *BAS4* and *PWL2* gene expression was detected in cDNA libraries generated from Δ*imp1* and WT infected leaf sheaths by real-time quantitative PCR (qPCR). Bars are the mean fold differences in effector gene expression in Δ*imp1* infected leaf sheath cDNAs compared to WT infected leaf sheath cDNAs after normalization against *M*. *oryzae* actin gene expression. Error bars are s.d. Values were calculated from three biological replicates with three technical replicates each.
(TIF)

S8 Fig The biotrophic interface is maintained in WT until 72 hpi. WT or Δ*imp1* strains expressing the fluorescently labeled apoplastic effector Bas4^GFP^ and the fluorescent BIC-accumulating cytoplasmic effector Pwl2^mCherry:NLS^ were inoculated onto leaf sheaths of CO-39 seedlings and viewed at 72 hpi by confocal microscopy. White arrows indicate appressoria on the leaf surface. Scale bars = 10 μm.
(TIF)

S9 Fig Plant innate immune responses are not elicited in cells infected with Δ*imp1* compared to WT at early infection stages. (A) *PBZ1* and *PR1A* defense gene expression was detected by qPCR in cDNA libraries generated from Δ*imp1* and WT infected leaf sheaths sampled at 24, 36 and 44 hpi. Bars are the average transcript abundances relative to rice actin expression determined from two biological replicates with three technical replicates each. Error bars are s.d. (*Student’s t test* *p ≤ 0.01, no star indicates no difference). (B) Infected cells were stained with 3,3′-diaminobenzidine (DAB). 100 cells were counted for DAB staining and experiments were repeated in triplicate. Scale bar = 10 um. Bars are s.d. Bars with different letters indicate significant difference (α ≤ 0.05, LSD).
(TIF)

S10 Fig Δ*imp1* mutant strains are resistant to rapamycin treatment *in planta*. Leaf sheaths infected with the indicated strains were treated with 10 μM rapamycin (Rap) at 24 hpi and viewed at 44 hpi. Stars indicate emerging IH in adjacent cells. Arrows indicate appressoria on the leaf sheath surface. Scale bars = 10 μm. NT = no treatment. Proportion of infected rice cells represented by these images are shown in **S1 Table.**
(TIF)

S11 Fig Treating infected leaf sheaths with amiodarone hydrochloride and 3-methyladenine affects the incidences of emerging IH in rice cells adjacent to primary infected cells. Treatment with the autophagy stimulator amiodarone hydrochloride (AM) at 36 hpi significantly increased the number of emerging WT and Δ*imp1* IH in cells adjacent to first infected cells by 44 hpi. Treatment with the autophagy inhibitor 3-methyladenine (3-MA) at 36 hpi significantly reduced the incidences of WT IH in adjacent cells compared to the no treatment (NT) control by 44 hpi. Data represent mean values ± s.d. of the number of emerging IH from 50 primary infected cells, repeated with three different leaf sheaths per strain (*Student’s t test* ***p ≤ 0.0001, no star indicates no difference).
(TIF)

S12 Fig Imp1^GFP^ localization is not affected by V-ATPase inhibition. Leaf sheaths infected with the Δ*imp1 IMP1*^*GFP*^ complementation strain expressing Imp1^GFP^ were treated with 10μM concanamycin A (ConA) or 1 μM bafilomycin A1 (BafA1) at 36 hpi and viewed at 44 hpi. White arrows indicate appressoria on the leaf sheath surface. Proportion of infected rice cells represented by these images are shown in **S2 Table.**
(TIF)

S1 Table Percentage of infected rice cells represented by the images in S10 Fig and Figs 9 and 10 when viewed at 44 hpi following the indicated treatments. (DOCX)

S2 Table Percentage of infected rice cells represented by the images in S12 Fig and Fig 11A when viewed at 44 hpi following the indicated treatments. (DOCX)

S3 Table Strains used for this study. (DOCX)

S4 Table Primers used in this study. (DOCX)
