# Correction: TOR-autophagy branch signaling via Imp1 dictates plant-microbe biotrophic interface longevity

**DOI:** 10.1371/journal.pgen.1008016

**Published:** 2019-02-28

**Authors:** Guangchao Sun, Christian Elowsky, Gang Li, Richard A. Wilson

There are typographical errors in the last sentence of the second paragraph of the Results section. The correct sentence is: This allele is annotated as encoding an uncharacterized 493 amino acid, 53.5 kDa integral membrane protein with five transmembrane domains which we name Imp1 (Fig 1A).
