# Supplementary figures and images for: TOR-autophagy branch signaling via Imp1 dictates plant-microbe biotrophic interface longevity
Source: PLoS Genet. 2018 Nov 21;14(11):e1007814. doi: 10.1371/journal.pgen.1007814 (PMC6281275; doi:10.1371/journal.pgen.1007814)

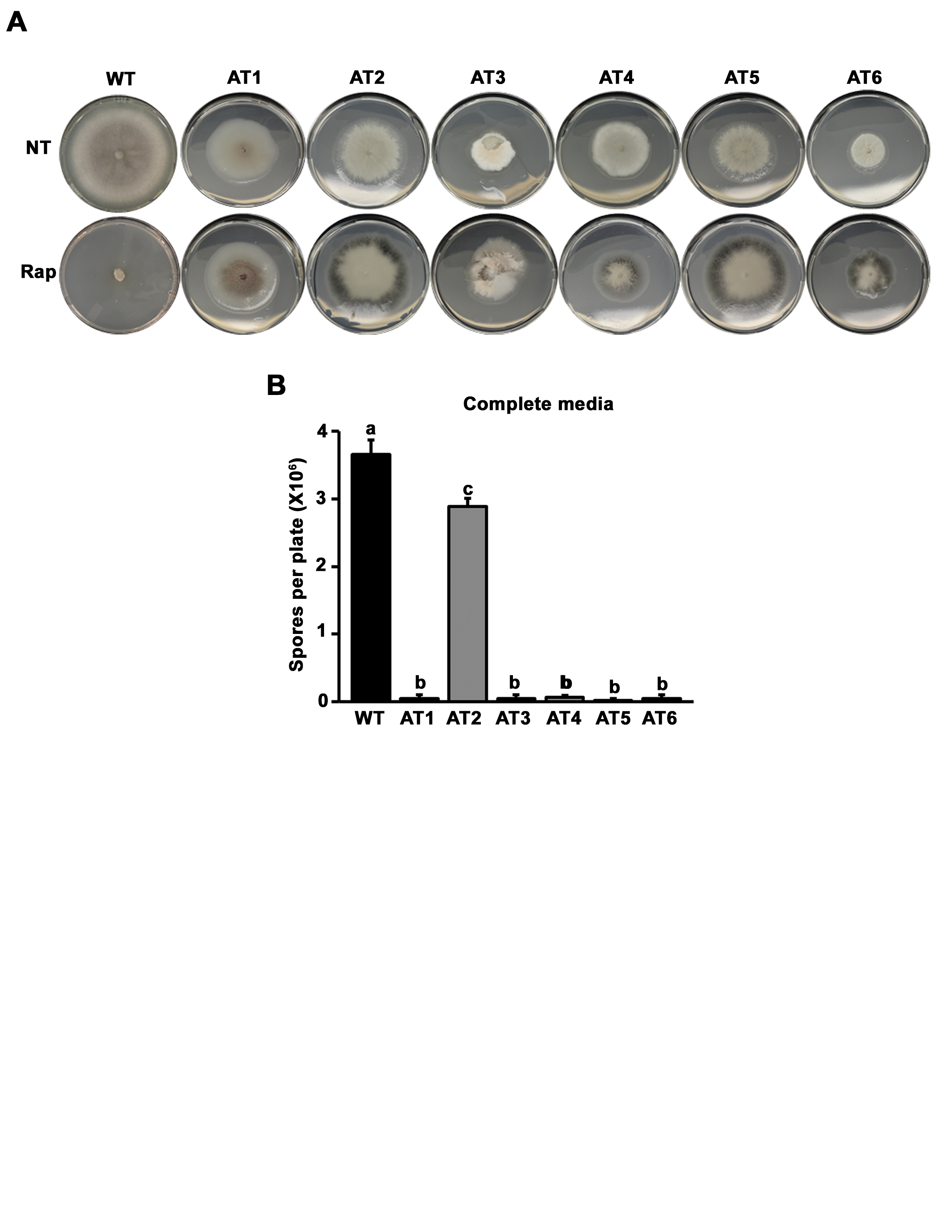

Supplement: S1 Fig — (A) Strains are shown after growing for 10 days on minimal media (MM) with 1% (w/v) glucose as the sole carbon source and 10 mM NH4+ as the sole nitrogen resource. Rap is 10 μM rapamycin. NT is no treatment. (B) Of the six Rap resistant mutant strains generated by ATMT, only AT2 sporulated at similar rates to WT. Bars are the mean number of spores harvested from three 10-day-old plates. Error bars are s.d. Bars with different letters indicate significant difference (α ≤ 0.05, Least significant difference (LSD)). (TIF) [file pgen.1007814.s001.tif]

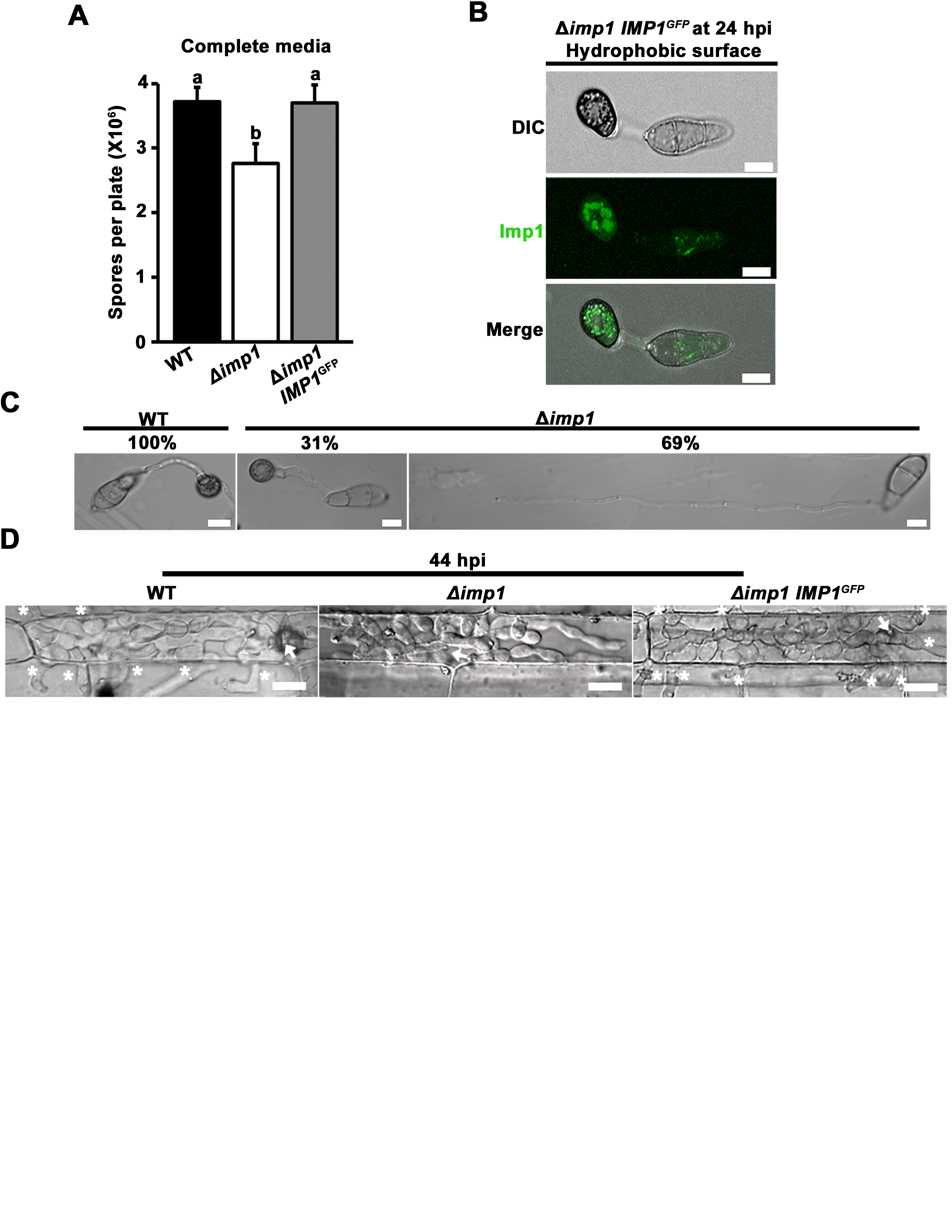

Supplement: S2 Fig — (A) The clean knockout strain of IMP1, Δimp1, sporulated at marginally reduced rates compared to WT and the Δimp1 IMP1GFP complementation strain. Bars are the mean number of spores harvested from three 10-day-old plates. Error bars are s.d. Bars with different letters indicate significant difference (α ≤ 0.05, LSD). (B) Following spore germination on artificial hydrophobic surfaces, Imp1GFP had, by 24 hpi, localized to compartments in the appressorium. (C) Appressorium formation on artificial hydrophobic surfaces in Δimp1 compared to WT. Images are representative of the observed phenotypes. % is the proportion of germinating spores displaying the indicated morphology by 24 hpi. (D) Biotrophic growth was impaired in Δimp1. Stars indicate emerging invasive hyphae (IH) in adjacent cells. Arrows indicate appressoria on the leaf sheath surface. Scale bars = 10 μm. (TIF) [file pgen.1007814.s002.tif]

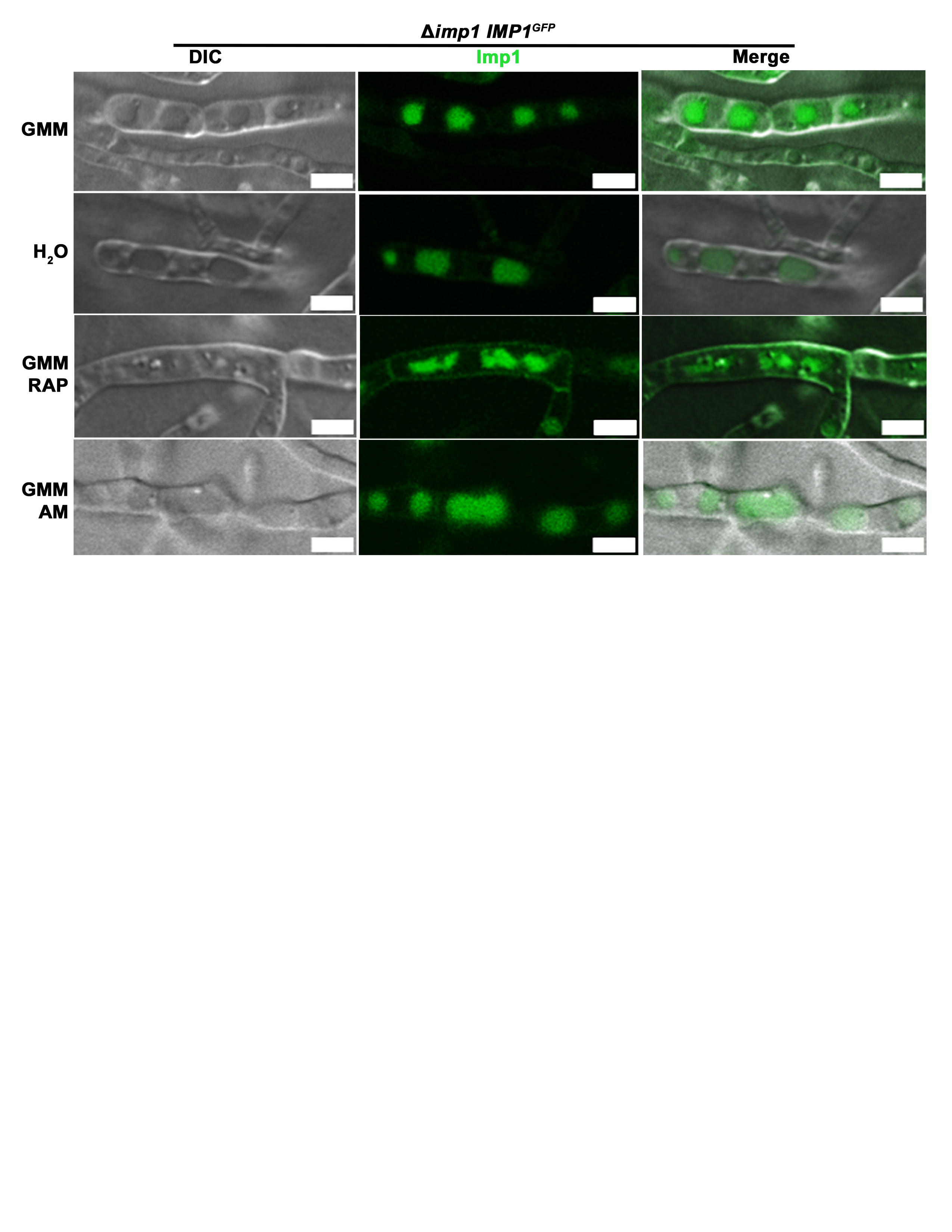

Supplement: S3 Fig — Vegetative mycelia were grown in the indicated treatments for 3 h. GMM = glucose minimal media. Rap = 1 μM rapamycin. AM = 1 μM amiodarone hydrochloride, a TOR-independent autophagy stimulator. Scale bar = 5 μm. (TIF) [file pgen.1007814.s003.tif]

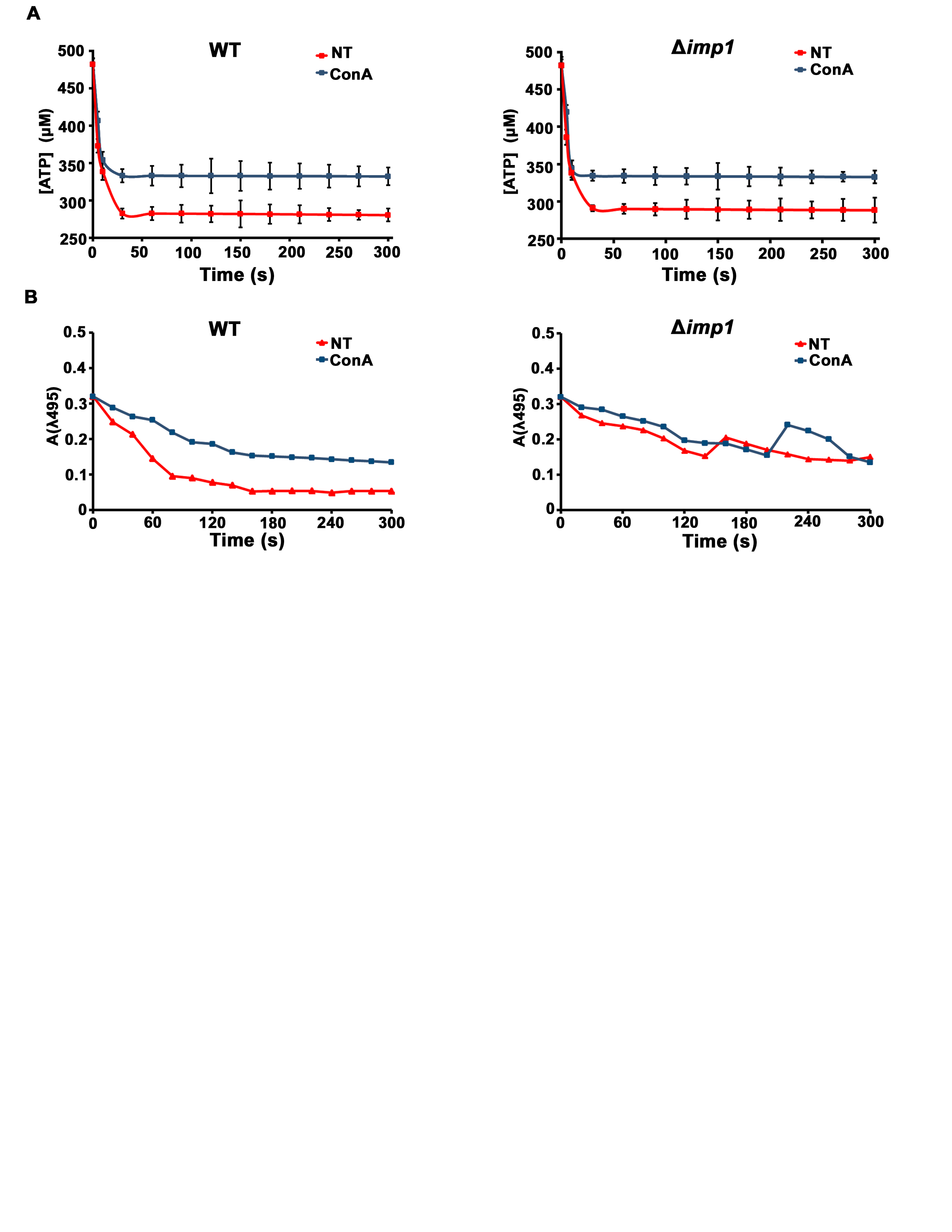

Supplement: S4 Fig — (A) V-ATPase-dependent ATP hydrolysis activity was not different in protoplast vesicles of Δimp1 and WT liberated from vegetative mycelia grown in glucose-rich complete media (CM). V-ATPase activity was determined as the reduction in the amount of ATP hydrolysed following treatment with 200 nM of the V-ATPase inhibitor concanamycin A (ConA) compared to the amount of ATP hydrolyzed by untreated samples (NT). (B) V-ATPase-dependent proton pumping activity, determined from the reduction of absorbance quenching of the ΔpH probe acridine orange, was not detectably different during early time points in protoplast vesicles of Δimp1 and WT liberated from vegetative mycelia grown in glucose-rich complete media (CM). However, differences in the rates of absorbance quenching emerged at later time points suggesting IMP1 is required for maintaining the pH gradient. (TIF) [file pgen.1007814.s004.tif]

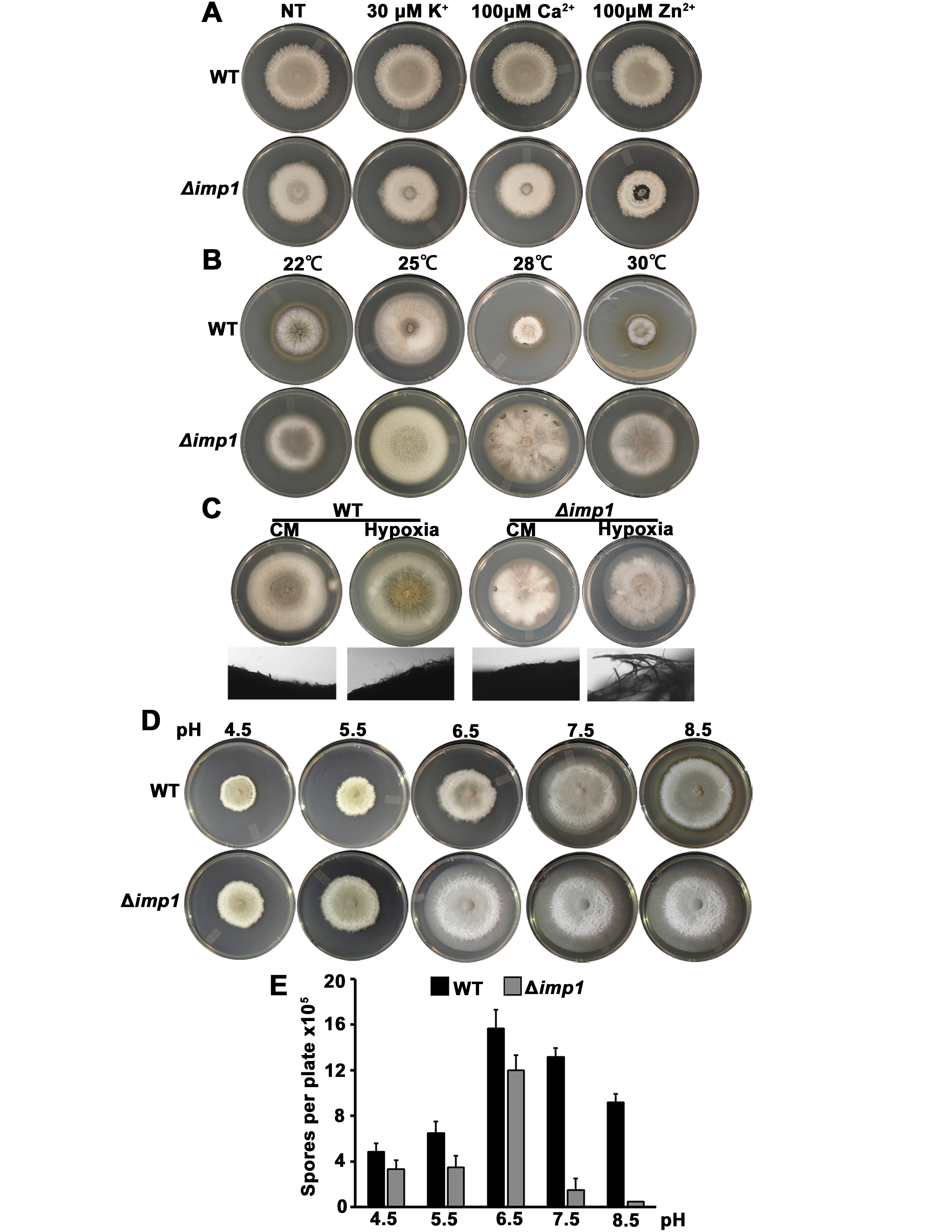

Supplement: S5 Fig — (A,B, D) Strains were grown for 10 days on defined glucose minimal media with the indicated treatments. NT = no treatment. (C) The strains were grown in 100 mm petri dishes filled half-full with 25 ml complete media (CM), per our normal protocol, or filled to the top with CM, leaving only a 2–5 mm space between the media surface and the lid, and sealed with parafilm to generate hypoxia stress. Plates were incubated for 12 days. (E) Spores were harvested from plates of the indicated pH at 12 days. Bars are the average of three independent replicates, error bars are s.d. (TIF) [file pgen.1007814.s005.tif]

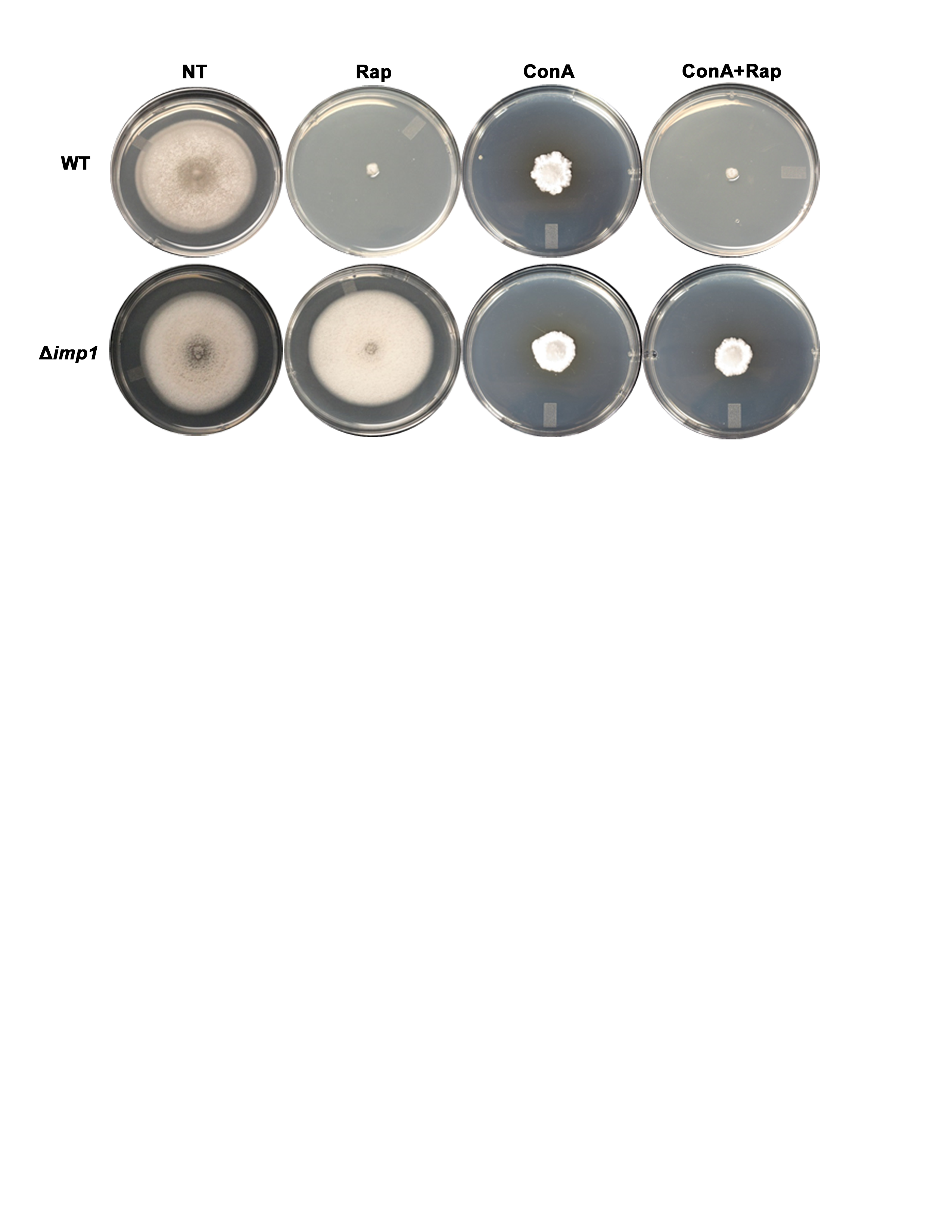

Supplement: S6 Fig — WT and Δimp1 were grown in CM supplemented with 50 nM ConA, 10 μM rapamycin or both for 12 days. NT = no treatment. (TIF) [file pgen.1007814.s006.tif]

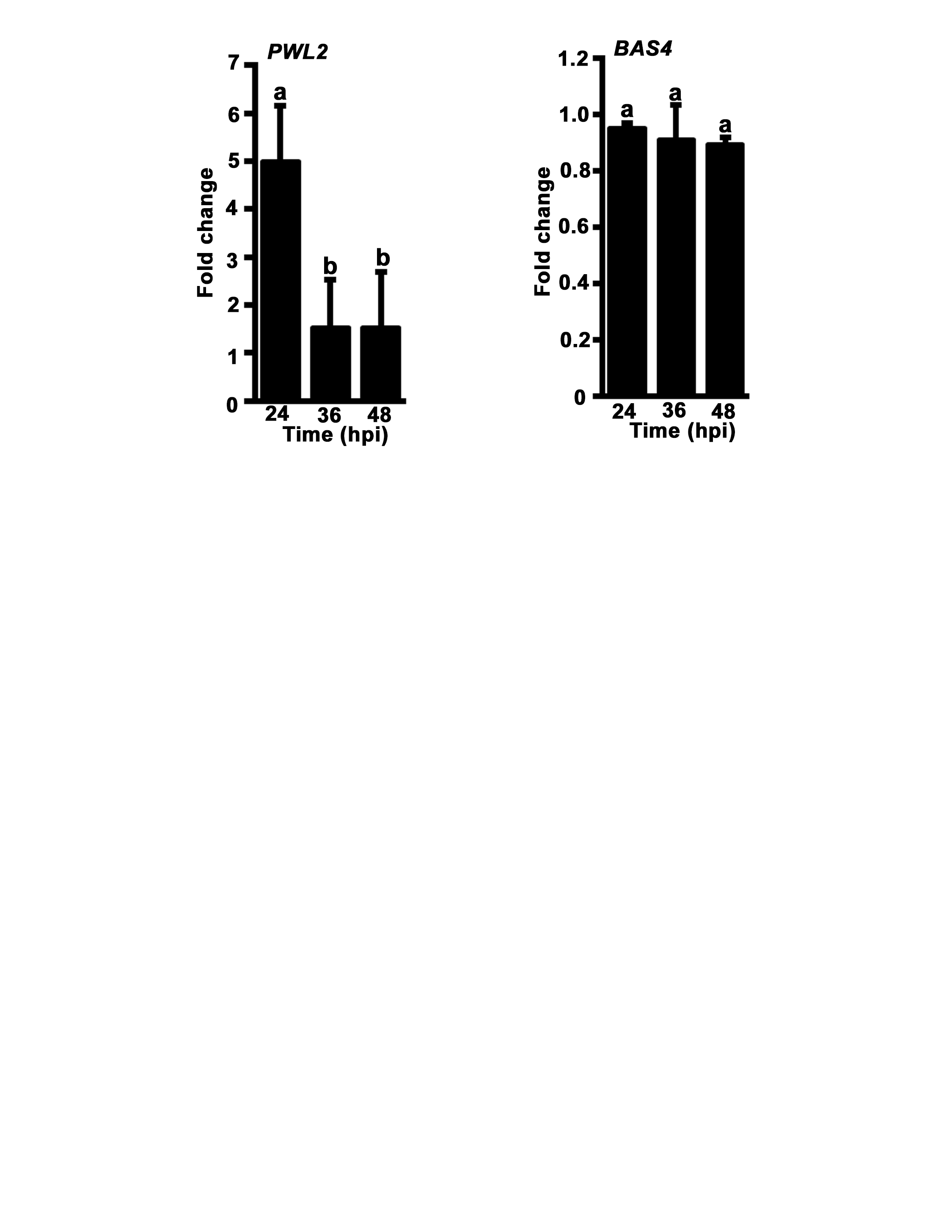

Supplement: S7 Fig — BAS4 and PWL2 gene expression was detected in cDNA libraries generated from Δimp1 and WT infected leaf sheaths by real-time quantitative PCR (qPCR). Bars are the mean fold differences in effector gene expression in Δimp1 infected leaf sheath cDNAs compared to WT infected leaf sheath cDNAs after normalization against M. oryzae actin gene expression. Error bars are s.d. Values were calculated from three biological replicates with three technical replicates each. (TIF) [file pgen.1007814.s007.tif]

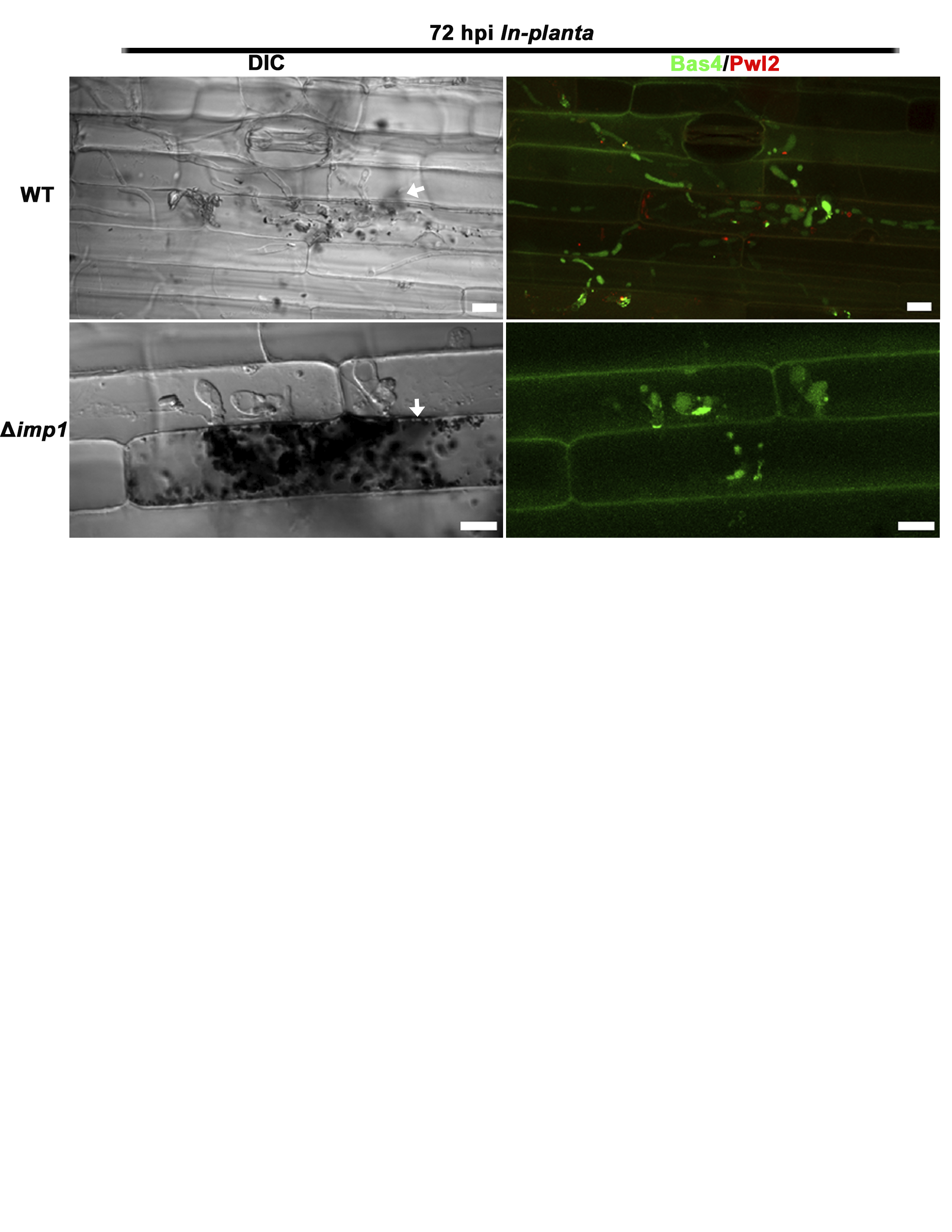

Supplement: S8 Fig — WT or Δimp1 strains expressing the fluorescently labeled apoplastic effector Bas4GFP and the fluorescent BIC-accumulating cytoplasmic effector Pwl2mCherry:NLS were inoculated onto leaf sheaths of CO-39 seedlings and viewed at 72 hpi by confocal microscopy. White arrows indicate appressoria on the leaf surface. Scale bars = 10 μm. (TIF) [file pgen.1007814.s008.tif]

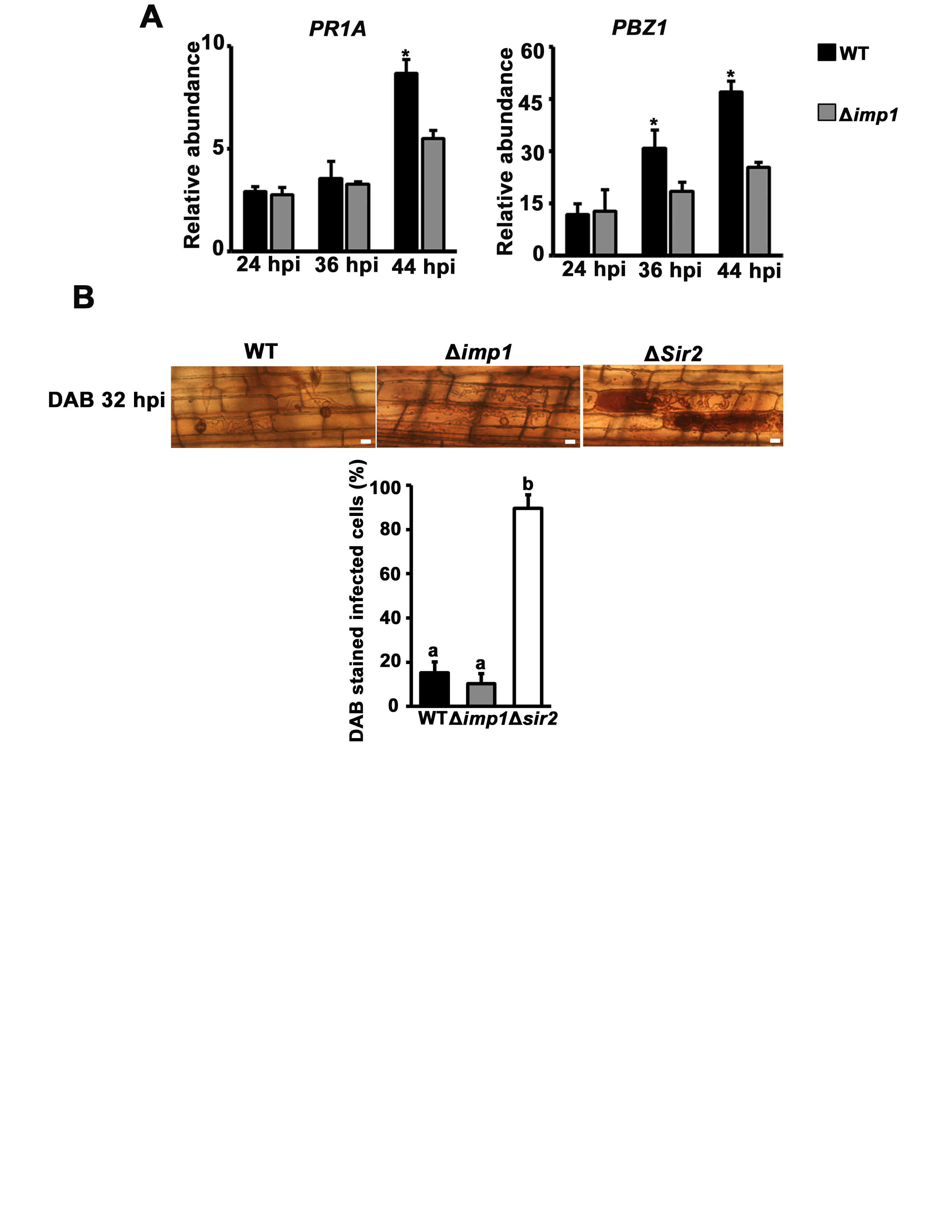

Supplement: S9 Fig — (A) PBZ1 and PR1A defense gene expression was detected by qPCR in cDNA libraries generated from Δimp1 and WT infected leaf sheaths sampled at 24, 36 and 44 hpi. Bars are the average transcript abundances relative to rice actin expression determined from two biological replicates with three technical replicates each. Error bars are s.d. (Student’s t test *p ≤ 0.01, no star indicates no difference). (B) Infected cells were stained with 3,3′-diaminobenzidine (DAB). 100 cells were counted for DAB staining and experiments were repeated in triplicate. Scale bar = 10 um. Bars are s.d. Bars with different letters indicate significant difference (α ≤ 0.05, LSD). (TIF) [file pgen.1007814.s009.tif]

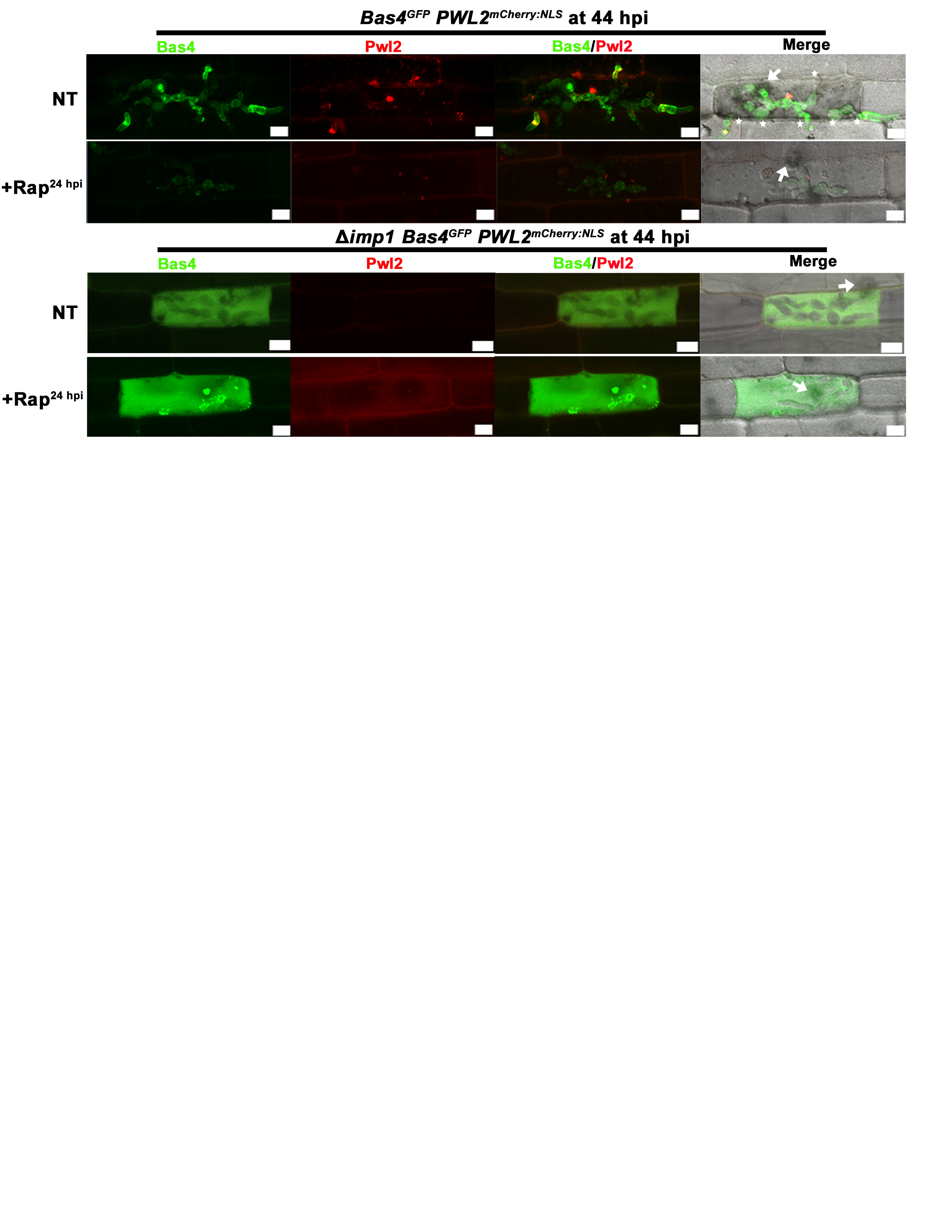

Supplement: S10 Fig — Leaf sheaths infected with the indicated strains were treated with 10 μM rapamycin (Rap) at 24 hpi and viewed at 44 hpi. Stars indicate emerging IH in adjacent cells. Arrows indicate appressoria on the leaf sheath surface. Scale bars = 10 μm. NT = no treatment. Proportion of infected rice cells represented by these images are shown in S1 Table. (TIF) [file pgen.1007814.s010.tif]

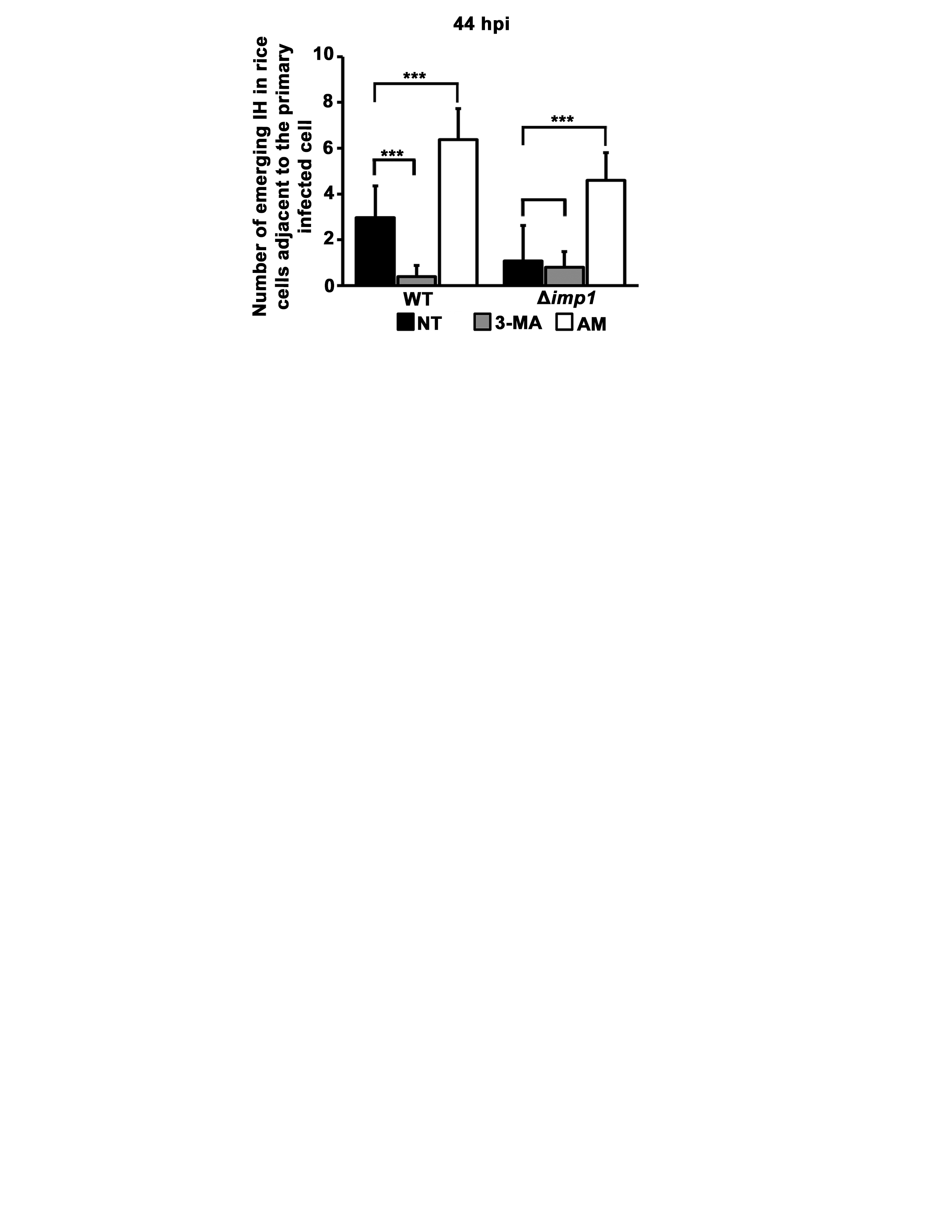

Supplement: S11 Fig — Treatment with the autophagy stimulator amiodarone hydrochloride (AM) at 36 hpi significantly increased the number of emerging WT and Δimp1 IH in cells adjacent to first infected cells by 44 hpi. Treatment with the autophagy inhibitor 3-methyladenine (3-MA) at 36 hpi significantly reduced the incidences of WT IH in adjacent cells compared to the no treatment (NT) control by 44 hpi. Data represent mean values ± s.d. of the number of emerging IH from 50 primary infected cells, repeated with three different leaf sheaths per strain (Student’s t test ***p ≤ 0.0001, no star indicates no difference). (TIF) [file pgen.1007814.s011.tif]

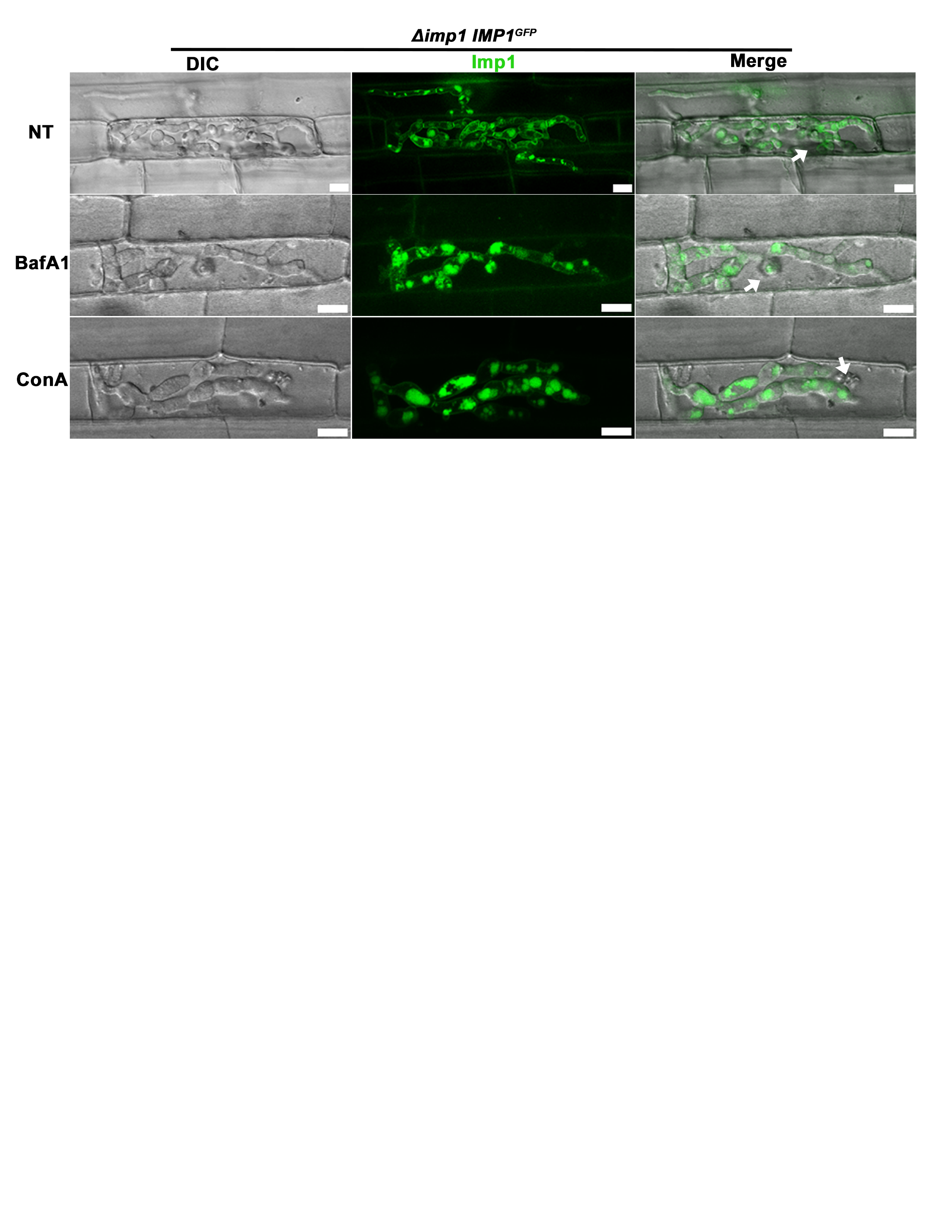

Supplement: S12 Fig — Leaf sheaths infected with the Δimp1 IMP1GFP complementation strain expressing Imp1GFP were treated with 10μM concanamycin A (ConA) or 1 μM bafilomycin A1 (BafA1) at 36 hpi and viewed at 44 hpi. White arrows indicate appressoria on the leaf sheath surface. Proportion of infected rice cells represented by these images are shown in S2 Table. (TIF) [file pgen.1007814.s012.tif]
