# Supplementary material for: TOR-autophagy branch signaling via Imp1 dictates plant-microbe biotrophic interface longevity
Source: PLoS Genet. 2018 Nov 21;14(11):e1007814. doi: 10.1371/journal.pgen.1007814 (PMC6281275; doi:10.1371/journal.pgen.1007814)
Supplement: S1 Table — (DOCX) [file pgen.1007814.s013.docx]

Table S1. Percentage of infected rice cells represented by the images in S10 Fig and Figs 9 and 10 when viewed at 44 hpi following the indicated treatments.

| Strain | Treatment added at | NT^a^ | | 10 μM Rap^d^ | |
| --- | --- | --- | --- | --- | --- |
|  |  | Mean^b^ (%) | S.D^c^ | Mean^b^ (%) | S.D^c^ |
| WT *BAS4^GFP^ PWL2^mCherry:NLS^* | 24 hpi | 93 | 2.6 | 82.0 | 4.0 |
| *∆imp1 BAS4^GFP^ PWL2^mCherry:NLS^* | 24 hpi | 100^e^ | 0 | 88.5 | 4.0 |

| Strain | Treatment added at | NT^a^ | | 2 μM AM^f^ | | 5 mM 3-MA^g^ | |
| --- | --- | --- | --- | --- | --- | --- | --- |
|  |  | Mean^b^ (%) | S.D^c^ | Mean^b^  (%) | S.D^c^ | Mean^b^  (%) | S.D^c^ |
| WT *BAS4^GFP^ PWL2^mCherry:NLS^* | 36 hpi | 92 | 3.0 | 89.1 | 4.0 | 86.7 | 5.0 |
| *∆imp1 BAS4^GFP^ PWL2^mCherry:NLS^* | 36 hpi | 100^e^ | 0 | 73.5 | 2.0 | 100 | 0 |

| Strain | Treatment added at | 10 μM ConA^h^ | | 10 μM BafA1^i^ | |
| --- | --- | --- | --- | --- | --- |
|  |  | Mean^b^  (%) | S.D^c^ | Mean^b^  (%) | S.D^c^ |
| WT *BAS4^GFP^ PWL2^mCherry:NLS^* | 36 hpi | 98 | 1.63 | 88.7 | 7.6 |
| *∆imp1 BAS4^GFP^ PWL2^mCherry:NLS^* | 36 hpi | 86 | 3.3 | 82.7 | 7.0 |

^a^NT: not treated, the original solvent for spore suspension was replaced by ddiH_2_O which was used to dissolve the treatments.

^b^Mean: values correspond to the average of 50 infected cells from each of three independent replicates.

^c^S.D: Standard deviation

^d^Rap: Rapamycin

^e^Represents the total percentage of all the phenotypes shown in Fig 10B.

^f^AM: Amiodarone hydrochloride

^g^3-MA: 3-Methyladenine

^h^ConA: Concanamycin A

^i^BafA1: Bafilomycin A1
