# Supplementary material for: TOR-autophagy branch signaling via Imp1 dictates plant-microbe biotrophic interface longevity
Source: PLoS Genet. 2018 Nov 21;14(11):e1007814. doi: 10.1371/journal.pgen.1007814 (PMC6281275; doi:10.1371/journal.pgen.1007814)
Supplement: S2 Table — (DOCX) [file pgen.1007814.s014.docx]

Table S2. Percentage of infected rice cells represented by the images in S12 Fig and Fig 11A when viewed at 44 hpi following the indicated treatments.

| Strain | Treatment added at | NT^a^ | | 10 μM BafA1^d^ | | 10 μM ConA^e^ | |
| --- | --- | --- | --- | --- | --- | --- | --- |
|  |  | Mean^b^ (%) | S.D^c^ | Mean^b^  (%) | S.D^c^ | Mean^b^  (%) | S.D^c^ |
| *∆imp1*:  *IMP1*^GFP^ | 36 hpi | 98 | 1.6 | 88.7 | 5.0 | 89.1 | 5.0 |

| Strain | Treatment added at | NT^a^ | | 5 mM 3-MA^g^ | |
| --- | --- | --- | --- | --- | --- |
|  |  | Mean^b^ (%) | S.D^c^ | Mean^b^  (%) | S.D^c^ |
| *∆imp1*:*IMP1*^GFP^ | 36 hpi | 100 | 0 | 89.3 | 2.5 |

^a^NT: not treated, the original solvent for spore suspension was replaced by ddiH_2_O which was used to dissolve the treatments.

^b^Mean: values correspond to the average of 50 infected cells from each of three independent replicates.

^c^S.D: Standard deviation

^d^BafA1: Bafilomycin A1

^e^ConA: Concanamycin A

^f^ AM: Amiodarone hydrochloride

^g^3-MA: 3-Methyladenine
