# Supplementary material for: TOR-autophagy branch signaling via Imp1 dictates plant-microbe biotrophic interface longevity
Source: PLoS Genet. 2018 Nov 21;14(11):e1007814. doi: 10.1371/journal.pgen.1007814 (PMC6281275; doi:10.1371/journal.pgen.1007814)
Supplement: S3 Table — (DOCX) [file pgen.1007814.s015.docx]

S3 Table. Strains used for this study.

| **Strains** | **Genotype** | **Reference** |
| --- | --- | --- |
| Guy11 | Wild type (WT) strain | [1] |
| AT1 to AT6 | Rapamycin insensitive suppressor mutants generated from WT by ATMT. | This study |
| ∆*imp1* | Deletant of Integral membrane protein 1- encoding gene *IMP1* (MGG_08120); made by replacing *IMP1* in WT with *ILV1* conferring sulphonyl urea resistance. | This study |
| ∆*fpr1* | Deletant of FKBP12 encoding gene (MGG_06035) made by replacing *FPR1* in WT with ILV1 conferring sulphonyl urea resistance. | [5] |
| ∆*imp1 IMP1^GFP^* | Complementation strain of ∆*imp1* generated by transforming the ∆*imp1* mutant with the pDL2 vector [2] carrying the full length *IMP1* gene fused to the GFP coding sequence and under its native promoter. | This study |
| ∆*imp1 OEIMP1^GFP^* | Complementation strain of ∆*imp1* generated by transforming the ∆*imp1* mutant with the full length *IMP1* gene fused to the GFP coding sequence, under the constitutive RP27 promoter, using the pDL2 vector [2]. | This study |
| WT *VMA2*^GFP^ | WT transformed with pDL2 carrying the *VMA2* gene (MGG_03244 encoding the putative V-ATPase V1 domain subunit B protein) fused to the GFP coding sequence and under the RP27 promoter. | This study |
| ∆*imp1* *VMA2*^GFP^ | ∆*imp1* transformed with pDL2 carrying the *VMA2* gene - encoding the V-ATPase V1 domain subunit B protein - fused to the GFP coding sequence and under the RP27 promoter. | This study |
| WT *BAS4^GFP^ PWL2^mCherry:NLS^* | WT strain carrying pBV591 [3] encoding Bas4^GFP^ and Pwl2^mCherry:NLS^. | [4] |
| ∆*imp1* *BAS4^GFP^ PWL2^mCherry:NLS^* #1 | ∆*imp1* strain carrying pBV591 generated from transforming plasmid pBV591 into ∆*imp1* background. | This study |
| ∆*imp1 BAS4^GFP^ PWL2^mCherry:NLS^* #5 | ∆*imp1* strain carrying pBV591 generated from transforming plasmid pBV591 into ∆*imp1* background. | This study |
| ∆*imp1 BAS4^GFP^ PWL2^mCherry:NLS^* # 2-1 | *IMP1* deletion in the WT *BAS4^GFP^ PWL2^mCherry:NLS^* background strain. | This study |
| ∆*sir2* | Deletant of *MoSIR2* encoding gene (MGG_10267); made by replacing *SIR2* in WT with *ILV1* conferring sulphonyl urea resistance. | [6] |

1. Talbot, NJ. On the Trail of a Cereal Killer: Exploring the Biology of *Magnaporthe grisea*. Ann. Rev. Micro. 2003; 57: 177 – 202.
2. Zhou X, Li G, Xu JR. Efficient approaches for generating GFP fusion and epitope-tagging constructs in filamentous fungi. Methods Mol. Biol. 2011; 722: 199-212.
3. Giraldo MC, Dagdas YF, Gupta YK, Mentlak TA, Yi M, Martinez-Rocha A et al. Two distinct secretion systems facilitate tissue invasion by the rice blast fungus *Magnaporthe oryzae*. Nat. Comm. 2013; 4: 1996.
4. Marroquin-Guzman M, Sun G, Wilson RA. Glucose-ABL1-TOR Signaling Modulates Cell Cycle Tuning to Control Terminal Appressorial Cell Differentiation. PLoS Genet*.* 2017; 13: e1006557.
5. Marroquin-Guzman M, Wilson RA. GATA-dependent glutaminolysis drives appressorium formation in *Magnaporthe oryzae* by suppressing TOR inhibition of cAMP/PKA signaling. PLoS Pathog. 2015; 11: e1004851.
6. Fernandez J, Marroquin-Guzman M, Nandakuma, R, Shijo S, Cornwell K M, Li G, et al. Plant defence suppression is mediated by a fungal sirtuin during rice infection by *Magnaporthe oryzae*. Molecular microbiology. 2014; 94: 70-88.
