# Supplementary material for: TOR-autophagy branch signaling via Imp1 dictates plant-microbe biotrophic interface longevity
Source: PLoS Genet. 2018 Nov 21;14(11):e1007814. doi: 10.1371/journal.pgen.1007814 (PMC6281275; doi:10.1371/journal.pgen.1007814)
Supplement: S4 Table — (DOCX) [file pgen.1007814.s016.docx]

S4 Table. Primers used in this study.

| Primer Name | DNA Sequence (5`-3`) |
| --- | --- |
| RB-1 | GGCACTGGCCGTCGTTTTACAAC |
| RB-2 | AACGTCGTGACTGGGAAAACCCT |
| RB-3 | CCCTTCCCAACAGTTGCGCAG |
| LB-1 | GGGTTCCTATAGGGTTTCGCTCATG |
| LB-2 | CATGTGTTGAGCATATAAGAAACCCT |
| LB-3 | GAATTAATTCGGCGTTAATTCAGT |
| AD-1 | NGTCGASWGANAWGAA |
| AD-2 | GTNCGASWCANAWGTT |
| AD-3 | WAGTGNAGWANCANAGA |
| *IMP1*-LF5’ | CTGGGTAGTAAGGTAAGGACGCA |
| *IMP1*-LF3’ | GTCGTGACTGGGAAAACCCTGGCGCGGAAAGACAAAGACATAGAAGTGG |
| *IMP1*-RF5’ | TCCTGTGTGAAATTGTTATCCGCTGGCTACTACAACGGCACACTCAT |
| *IMP1*-RF3’ | AGGAGCAGGAAGATGACCAGCG |
| *IMP1*-NesF | CAGGTCGTTGTGCTCTTTTCG |
| *IMP1*-NesR | CAAGGACCAGACCGAGGAGAA |
| *ILV1* -M13F:IL | CGCCAGGGGTTTTCCCAGTCACGACGTCGACGTGCCAACGCCACAG |
| *IL*Split | AAGCATGTGCAGTGCCTTC |
| M13R:*LV1* | AGCGGATAACAATTTCACACAGGAGTCGACGTGAGAGCATGCTAA |
| *LV1*Split | CGCCCGGCCGACATCC |
| P(RP27)-*IMP1*-GFP-FW: | TTTCGTAGGAACCCAATCTTCAAAATGCCTTCCAGGTTGGCATCA |
| P(IMP1)-*IMP1*-GFP-FW: | TATAGGGCGAATTGGGTACTCAAATTGGTTCTCATTCCTGTGTGC |
| *IMP1*-GFP-RE: | CCCGGTGAACAGCTCCTCGCCCTTGCTCACAATCATCTCCCTGGGATT |
| P(RP27)-*VMA2*-GFP-FW: | TTTCGTAGGAACCCAATCTTCAAAATGGCGGACCCTCGAGAGT |
| *VMA2*-GFP-RE: | CCCGGTGAACAGCTCCTCGCCCTTGCTCACGGCATCGATCAAGTTTTCCTC |
| q*PWL2*-Fw | ATGAAATGCAACAACATCATC |
| q*PWL2*-Re | CCGTGCCTGTTATAATATTG |
| q*BAS4*-Fw | ATGCAGCTCTCATTCTCAGC |
| q*BAS4*-Re | GGTTAGGGCATTTCCTAACG |
| qActin-U1 | TCGACGTCCGAAAGGATCTGT |
| qActin-L1 | ACTCCTGCTTCGAGATCCACATC |
| q*RS2*-Fw | GGTTGCCTCGCCCGCTG |
| q*RS2*-Re | CGCTTGCCGTCCCTGAGG |
| q*RS3*-Fw | TCGTTCAGCCCGTCAGCCAA |
| q*RS3*-Re | CTCCTGCTCCTCACCCTCACCC |
| q*ATG8*-Fw | CAGGTCGCCGAAGGTGTTCTC |
| q*ATG8*-Re | GCCACCATCGACAAGAAGAAGTACC |
| β-Tubulin-Fw | CGCGGCCTCAAGATGTCGT |
| β-Tubulin-Re | GCCTCCTCCTCGTACTCCTCTTCC |
| *OsACT2-U1* | CTGAAGAGCATCCTGTATTG |
| *OsACT2-L1* | GAACCTTTCTGCTCCGATGG |
| *HPH-*Fw | GAAAAAGCCTGAACTCACCGCG |
| *HPH*-Re | GAGTGCTGGGGCGTCGGT |
| q*NIA1* Fw | TGGCAACCGAACAGAGGAAGAC |
| q*NIA1* Re | TCAGAAAAACAACAAATCATCATCCTTCC |
| qRice_*PBZ1*-Fw | CTACTATGGCATGCTCAAGAT |
| qRice_*PBZ1*-Re | ATAGAAAGGCACATAAACACAA |
| qRice_*PR1a*-Fw | TCTTCATCACCTGCAACTACTC |
| qRice_*PR1a*-Re | ATTCATCGGATTTATTCTCACC |
